# Identification of A Novel Gene Signature Combining Ferroptosis- and Immunity-Related Genes for Prognostic Prediction, Immunotherapy and Potential Therapeutic Targets in Gastric Cancer

**DOI:** 10.7150/jca.87223

**Published:** 2023-10-16

**Authors:** Liwei Wang, Zhuozhuo Li, Zi Li, Yuanyuan Ren, Lu Qian, Yi Yu, Wenzhen Shi, Yuyan Xiong

**Affiliations:** 1 Xi'an Key Laboratory of Cardiovascular and Cerebrovascular Diseases, Xi'an No.3 Hospital, the Affiliated Hospital of Northwest University, Xi'an, Shaanxi, P.R. China.; 2 Key Laboratory of Resource Biology and Biotechnology in Western China, Ministry of Education, School of Medicine, Northwest University, Xi'an, Shaanxi, P.R. China.; 3 Department of Endocrinology, Xi'an No.3 Hospital, the Affiliated Hospital of Northwest University, Xi'an, Shaanxi, P.R. China.; 4 Medical Research Center, Xi'an No.3 Hospital, the Affiliated Hospital of Northwest University, Xi'an, Shaanxi, P.R. China.

**Keywords:** Gastric cancer, ferroptosis, immunity, prognosis, risk model

## Abstract

Gastric cancer (GC) is one of the most prevalent cancers worldwide. Ferroptosis and the immune status of tumor tissue play vital roles in the initiation and progression of GC. However, the role and functional mechanisms of ferroptosis- and immunity-related genes (FIRGs) in GC pathogenesis and their correlations with GC prognosis have not been elucidated. We aim to establish a prognostic prediction model based on the FIRGs signature for GC patients. Differentially expressed genes were screened from the Cancer Genome Atlas (TCGA) GC cohorts. The least absolute shrinkage and selection operator (LASSO) regression was performed to establish a FIRGs-based risk model. This gene signature with 7 FIRGs was identified as an independent prognostic factor. A nomogram incorporating clinical parameters and the FIRG signature was constructed to individualize outcome predictions. Finally, we provided *in vivo* and *in vitro* evidence to verify the reliability of FIRG signature for GC prognosis, and validate the expression and function of FIRGs contributing to the development and progression of GC. Herein, our work represents great therapeutic and prognostic potentials for GC.

## Introduction

Gastric cancer (GC) is one of the most common cancers worldwide with a poor prognosis 1, 2. Around 1.1 million new cases and 769,000 deaths of GC were estimated by the World Health Organization (WHO) in 2020. Traditional prognostic approaches, such as histopathological diagnosis and tumor staging systems, cannot accurately predict the prognosis of GC patients. We still need to explore alternative approaches to predict the prognosis of patients with GC, and guide clinical management for GC treatment. Thus, developing a method for accurately stratifying risk and new biomarkers for GC patients is essential.

Ferroptosis, a newly discovered iron-dependent form of programmed but non-apoptotic cell death, is caused by excessive iron accumulation, lipid peroxidation, and the destruction of intracellular redox balance 3. More recently, a growing body of research has demonstrated that ferroptosis plays a critical role in a wide range of illnesses, particularly cancer 4, 5. Ferroptosis as a vital feature of tumor biology may offer novel strategies for cancer diagnosis and therapy 6. Moreover, ferroptosis is acknowledged as a type of immunogenic cell death (ICD), suggesting that it may cause an innate or adaptive immunological response 7. Recently, researchers revealed the direct crosstalk between the immune system and ferroptosis 7. Clinically, ferroptosis is considered to be a promising target for cancer immunotherapy. Wang et al. reported that immunotherapy-activated CD8^+^ T cells increase tumor cell lipid peroxidation and sensitize tumors to ferroptosis 8. Recently, it has been revealed that anti-PD-1/PD-L1 therapy resistance is attributed to the suppression of innate immunity and tumoral ferroptosis 9. Therefore, investigating the roles of ferroptosis- and immunity-related genes (FIRGs) in GC may open up an avenue for the discovery of novel therapeutic strategies of GC resistance.

In this study, we identified prognosis-related ferroptosis and immune genes from the Cancer Genome Atlas (TCGA) stomach adenocarcinoma (STAD) cohorts, one of the most common histological subtypes of primary GC. A FIRG signature was established, and its prognostic value was validated in GC patients. We also constructed a nomogram based on the integration of the FIRG signature to predict individual overall survival (OS). Furthermore, we analyzed and discussed the immune status of subgroups with different scores identified by this model. Herein, this study suggests that the FIRG is a reliable gene signature for predicting prognosis in patients with GC and may benefit the understanding of GC pathogenesis and the exploration of novel targets for GC therapy.

## Materials and methods

### Acquisition of related genes

Ferroptosis-related and immunity-related genes were collected from the GeneCards (https://www.genecards.org/) database 10, which provides comprehensive information on human genes. The term “ferroptosis” was used as the keyword for the search, and genes with relevance scores >1 were taken. In the same way, immunity-related genes were acquired.

### Collection of datasets

The RNA-seq data and clinical characteristics of the TCGA STAD cohorts were obtained from the TCGA website (https://portal.gdc.cancer.gov/) for training. The large-scale genome sequencing was performed before treatment in those patients, as TCGA focuses on untreated primary cancers 11. Participants without detailed expression and clinical data or with 0-day follow-up duration were excluded 12. According to these criteria, 348 STAD and 32 normal samples were obtained and selected for the training cohorts. For the validation datasets, we downloaded the expression matrices and platforms of the GSE84437 (GPL6947), GSE62254 (GPL570), and GSE15459 (GPL570) from the GEO website (https://www.ncbi.nlm.nih.gov/geo/). Log2 transformation and normalization were employed for the expression profiles. The average expression level was retained for duplicate genes. The ComBat function of the sva package (https://bioconductor.org/packages/release/bioc/html/sva.html) in R software 4.2.1 (https://www.r-project.org/) was applied to remove the batch effects. The detailed clinic parameters were listed in Supplementary Table 1.

### Differential expression analysis

Differential expression analysis of genes between TCGA STAD and normal stomach tissue was conducted by the edgeR package (https://bioconductor.org/packages/release/bioc/html/edgeR.html) and visualized as volcano plots. P value (p) <0.05 and |fold change (FC)|>1 would be considered statistically significant for identifying differentially expressed genes (DEGs) 13. The intersection of the DEGs, ferroptosis-related genes, and immune-related genes, as visualized in a Venn diagram, were selected for further analysis.

### Construction and validation of the prognostic gene signature

Univariate Cox proportional hazards regression analysis was performed on each FIRG to screen genes significantly associated with OS in the TCGA training set 14, 15. Next, the LASSO Cox regression method was applied to those identified genes via the R software package glmnet 16, 17. Based on the optimal lambda value, the candidate genes were selected to construct the model and a risk formula. A prognostic risk score was calculated for each patient using the following equation: risk score = expression level of gene1*y1 + expression level of gene2*y2 + ... + expression level of genex*yx, where y represents the corresponding regression coefficient. The median risk score was considered the cutoff value to categorize TCGA STAD patients into high-risk and low-risk groups. The same formula and cutoff value were applied to three GEO validation datasets for the evaluation of model effectiveness.

Univariate and multivariate Cox proportional hazards regression analyses were performed to test whether the FIRG-based prognostic model was an independent prognostic factor when combined with clinical variables. A Kaplan-Meier (K-M) survival curve was constructed, and the log-rank test was used to assess the survival differences between groups. The sensitivity and specificity of the prognostic performance were examined by ROC curve analysis and visualized via the R package survivalROC 18. The AUC values indicated discrimination.

### Construction and validation of the nomogram

A prognostic nomogram was established to evaluate the survival probability for STAD patients in 1, 3, or 5 years via the rms R package. Age, gender, pathological stage, pathological T stage, pathological N stage, pathological M stage, and risk score were independent parameters. The C-index and calibration curves were used to calculate the discrimination and calibration between the nomogram-predicted value and the true survival.

### Gene set enrichment analysis (GSEA)

GSEA was performed using the R package clusterProfiler to determine the related pathways and molecular mechanisms of the high-risk and low-risk groups in the TCGA STAD cohorts 19. The whole genome of RNA-seq data in TCGA STAD cohorts was gene list and the high-risk and low-risk groups were used as the phenotype labels. The metric for ranking genes parameter was Signal2Noise. The reference gene set was “c2.cp.kegg.v7.5.1.entrez.gmt.” Significant pathway enrichment was identified by the normalized enrichment score (|NES|>1), p<0.05, and false discovery rate (FDR) <0.25 20.

### Functional enrichment analysis

Gene functional analysis is crucial in converting molecular results from high-throughput technologies into biological significance 21, 22. The clusterProfiler package in R software was used to perform statistical analysis and to visualize the functional profiles of the FIRGs, including Gene Ontology (GO) and Kyoto Encyclopedia of Genes and Genomes (KEGG) pathway enrichment analysis 19. Adjusted p-value (adj. P) <0.05 was considered the cutoff value for significance.

### Assessment of immune cell infiltration and immune microenvironment

ESTIMATE is a method that determines the fractions of stromal and immune cells based on gene expression signatures in tumor samples. It was applied to evaluate the tumor microenvironment (TME) of each patient with STAD, along with stromal score (substrate cells in the tumor tissue), immune score (extent of immune cell infiltration), ESTIMATE score (the summation of stromal and immune scores from individual cases), and tumor purity by the estimate R package 23. CIBERSORT is an analysis tool to estimate the abundances of member cell types in a mixed cell population based on the expression profiles. This deconvolution algorithm was used to calculate the proportion of 22 immune cells in each patient with STAD 24. The sum of the 22 immune cell type fractions in each sample was 1. By applying the single-sample gene set enrichment analysis (ssGSEA) method from the R package GSVA 25, we calculated the extent of infiltration of 28 immune cell types according to the expression levels of genes in 28 published gene sets for immune cells 26.

### Cell culture

Human gastric cancer cell lines, MGC803 cells and MKN45 cells, were bought from Procell (Wuhan, China), and were cultured in DMEM medium (DMEM; Sigma, USA) supplemented with 10% fetal bovine serum (Gemini, USA), 100 µg/mL streptomycin, and 100 U/mL penicillin at 37 ^o^C in a humidified atmosphere containing 5% CO_2_. The cell lines used in the study were authenticated by short tandem repeat DNA profiling.

### Production and transmission of lentiviruses

To produce lentivirus particles, HEK293T cells were transfected with the empty vector pLKO.1 with targeted shRNA sequences for SPARC, NOX4, GPX3 knockdown, or pLKO.1 with helper plasmid pMD 2.G. GP-Transfer-Mate was utilized as a transfection reagent for low-scale preps at a ratio of 4:3 GP-Transfer-Mate/DNA. Additionally, the ratio of the lentiviral backbone constructs pSPAX2 and pMD2.G was 4:3:1. To eliminate the cells, the viral supernatant was collected 24 and 48 hours after transfection, spun at 1500 rpm for 5 minutes, flash frozen, and stored at 80°C. The MGC803 cells and MKN45 cells were transduced with lentivirus when they were about 80% confluent. MGC803 cells and MKN45 cells were placed in 1.5 ml of media with 250 μL of lentivirus for 24 hours.

### Cell proliferation

The experiment was conducted according to the Cell Counting Kit-8 Reagent Kit (Beyotime). Usually, 100 µl of 2000 cells per well for cell proliferation assays and 100 µl of 5000 cells per well for cytotoxicity assays. Add 10 µl of CCK-8 solution per well. Cell proliferation was examined after keeping the cells in a 5% CO2/37°C humidified incubator for 24 h, 48 h, 72 h, and 96 h. The absorbance at 450 nm was measured using a microplate reader.

### Transwell assay

For the cell migration assay, the chambers were not coated. The chambers were coated with Matrigel (BD Biosciences) for the cell invasion assay. First, 20,000 cells in 100 μL of medium without serum were added to the upper chamber, while medium containing serum but no cells were added to the lower chamber. The cells were incubated at 37°C for 24 hours. Cells that did not cross the membrane were gently removed with cotton balls, and those that crossed the membrane were fixed with 4% paraformaldehyde and stained with crystal violet for 15 minutes. Under a microscope, stained cells were counted in five randomly selected areas, and the mean value was calculated.

### Wound healing migration assay

Cells were cultured in 6-well culture dishes in a complete medium. When the cells reach 70% to 90% confluence, the wound is scraped along the length of the culture dish with a pipette tip (200 μl). Cells were photographed through a microscope, and the size of the wound was measured (s0). After changing the medium to serum-free medium, the cells were cultured in the incubator for 24 h. After 24 h, the size of the wound (s24) was measured and compared with s0.

### Statistical analysis

DEGs were screened using the Wilcoxon test. Univariate Cox analysis was performed to screen relevant genes with prognostic values. K-M survival curves were generated and compared between the two groups using the log-rank test. All statistical analyses were performed using R version 4.2.1 (https://www.r-project.org/) and its adequate packages. Statistical significance was set at *p* < 0.05.

## Results

### Identification of differentially expressed FIRGs and functional analysis

**Figure 1:**
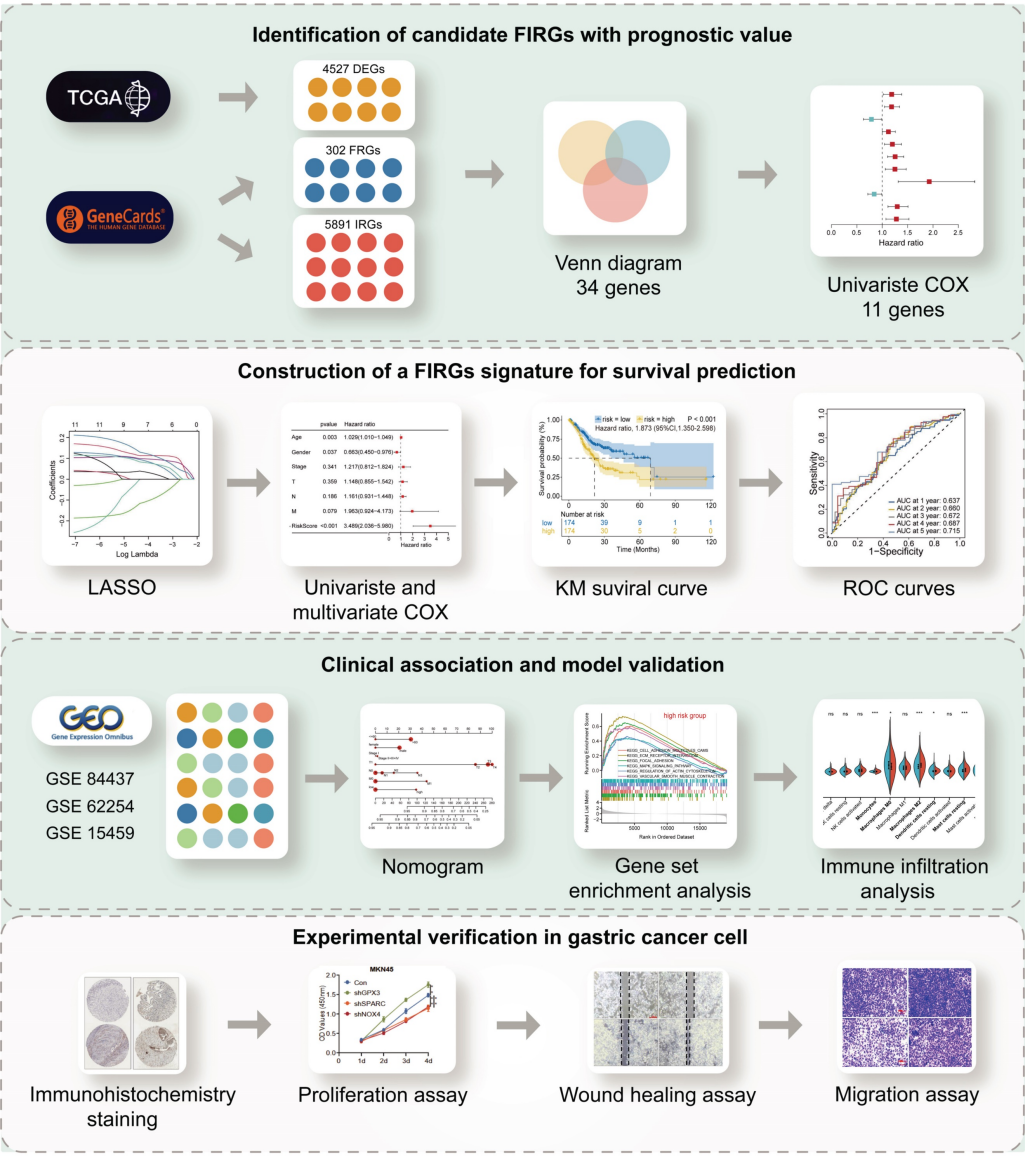
The flow chart summarizes the scheme performed to construct prognostic gene signatures of stomach adenocarcinoma (STAD)

**Figure 2:**
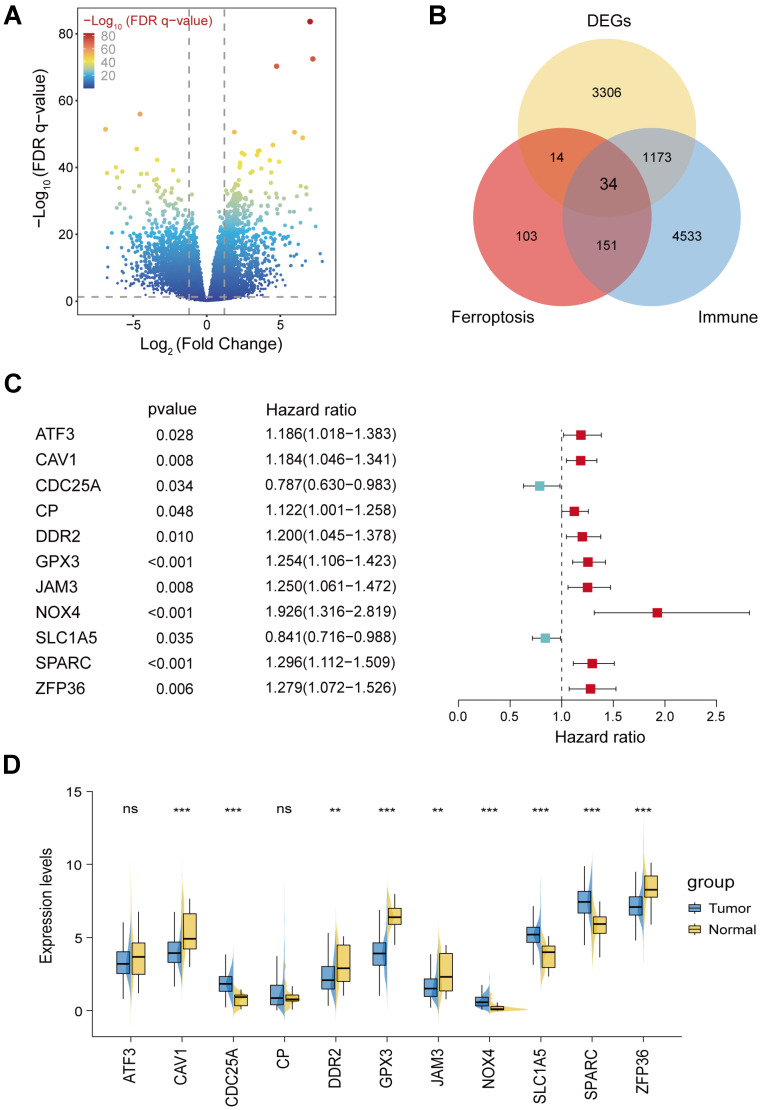
** Identification of differentially expressed ferroptosis- and immunity-related genes (FIRGs) and selection of the FIRGs associated with the survival of TCGA STAD patients.** (A and B) Volcano plot (A) of the 4527 DEGs and Venn diagram (B) of the 34 differentially expressed FIRGs in the STAD cohorts of the TCGA database. (C) Forest plot of the univariate Cox regression analysis with the FIRGs. (D) Expression levels of survival-related genes in tumor and normal tissues. For all, ns: not significant, *p<0.05, **p<0.01 and ***p<0.001.

The flowchart of this study was illustrated in Figure 1. STAD cohort data consisting of 348 STAD patients with detailed clinic parameters were retrieved from TCGA (Supplementary Table 1). The K-M survival curves and log-rank test for clinicopathological parameters, including overall stage, tumor (T), node (N), and metastasis (M) were shown in Figure S1A-D. 4527 STAD differentially expressed genes (DEGs) were identified in the TCGA cohorts, of which 2192 genes were upregulated, and 2335 genes were downregulated, as shown in the volcano plot (Figure 2A). To identify the gene set involved in the process of ferroptosis and immune response in *Homo sapiens*, a total of 302 ferroptosis-related and 5891 immune-related genes with a relevance score of >1, were screened from the GeneCards. The intersection of STAD DEGs, ferroptosis-related genes, and immune-related genes, containing 34 differentially expressed FIRGs, was visualized in a Venn diagram (Figure 2B). These 34 FIRGs were subjected to functional analysis, including GO and KEGG analysis. The KEGG pathway analysis demonstrated that the FIRGs were mainly enriched in signaling pathways of neurodegeneration-multiple diseases, ferroptosis, and advanced glycation end product (AGE)-receptor for AGE (RAGE) signaling pathway in diabetic complications (Figure S2A). As revealed by Gene Ontology, these FIRGs were mainly enriched in the biological processes (BPs) of tissue remodeling, regulation of extracellular matrix disassembly, and tissue homeostasis (Figure S2B), in the cellular component categories (CCs) of early endosome, focal adhesion, and cell-substrate junction (Figure S2C), and in the molecular functions (MFs) of RNA polymerase II-specific DNA-binding transcription factor binding, chaperone binding, and copper ion binding (Figure S2D). The univariate Cox regression analysis was performed to evaluate the prognosis significance of these FIRGs, which indicated that 11 FIRGs were remarkably associated with OS (Figure 2C). Among them, CDC25A and SLC1A5 displayed protective effects against STAD, while the rest genes (ATF3, CAV1, CP, DDR2, GPX3, JAM3, ZFP36, NOX4, and SPARC) were prognostic risk genes for STAD. Furthermore, we analyzed the expression levels of these genes in normal subjects and STAD patients. As shown in Figure 2D, significant downregulation of ATF3, CAV1, DDR2, GPX3, JAM3 and ZFP36, and prominent upregulation of CDC25A, NOX4, SLC1A5, and SPARC were observed in STAD patients.

### Construction and validation of the FIRGs prognostic risk evaluation model in the TCGA training and GEO cohorts

**Figure 3:**
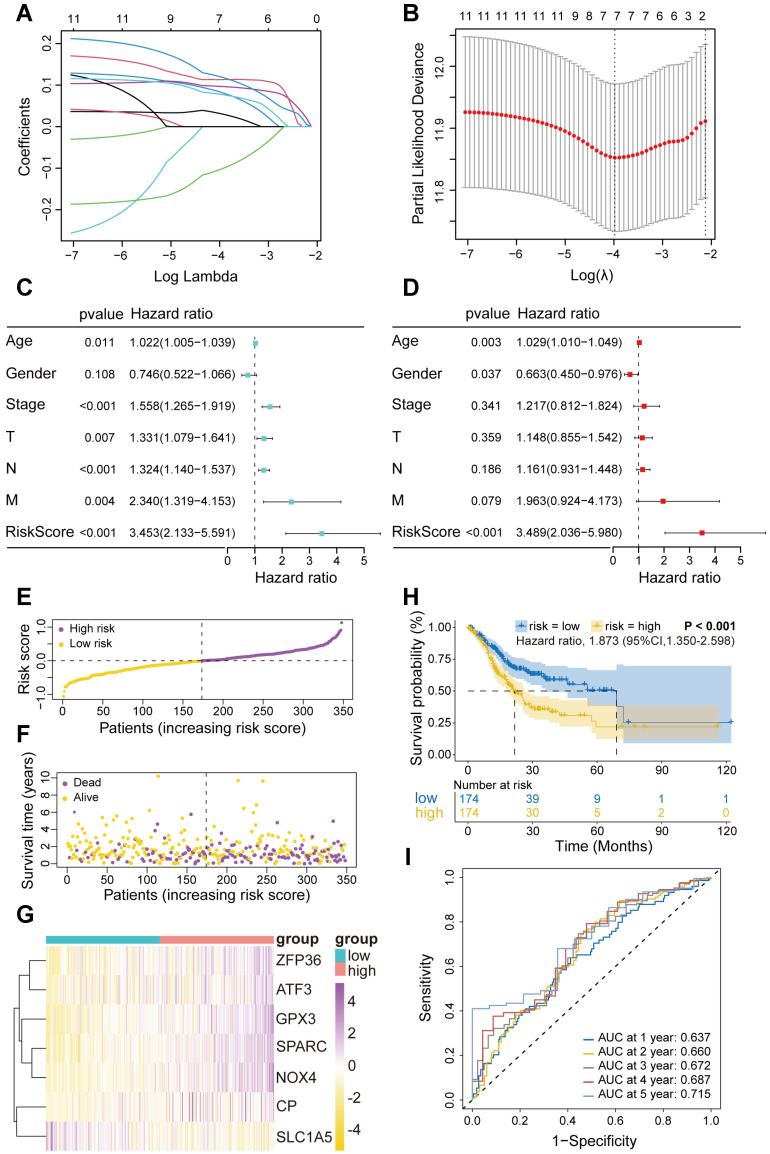
** Construction and validation of the FIRGs prognostic risk evaluation model in the TCGA training cohort and the evaluation of its independent prognostic value.** (A) LASSO coefficient profiles of the 11 survival-related genes. (B) Cross-validation for tuning parameter (lambda) screening in the LASSO regression model. (C and D) Forest plots of the (C) univariate and (D) multivariate Cox regression analysis in TCGA STAD cohorts. (E and F) The distributions of the risk score, survival time, and status of patients in TCGA STAD training cohorts. (G) Heatmap of the gene-expression profiles of the FIRGs signatures in TCGA STAD training cohorts. (H) Kaplan-Meier curves of the gene signature in TCGA STAD training cohorts. (I) The time-dependent ROC curves of the prognostic gene signature in TCGA STAD training cohorts.

Next, these 11 FIRGs were subjected to LASSO Cox regression analysis to construct a prognostic risk evaluation model in the TCGA training cohort. Coefficients of independent variables in LASSO regression were shown in Figure 3A. Based on the optimal log value of lambda (λ=7), 7 genes (SPARC, NOX4, SLC1A5, GPX3, CP, ZFP36, and ATF3) were identified (Figure 3B). The features of these 7 FIRGs, including biological processes, functions, and corresponding coefficients are described in Supplementary Table 2. GO analysis indicated that they were involved in glucose/energy metabolism (SLC1A5, CP, ATF3), extracellular matrix binding (SPARC), nucleotide binding (NOX4), RNA binding (ZFP36), and transcription factor binding (GPX3), respectively (Supplementary Table 2). Next, based on their mRNA expression levels and the coefficients from LASSO Cox regression analysis, each STAD patient in the TCGA database was assigned a risk score using the following formula: Risk Score = SPARC*0.119965 + NOX4*0.113284 + SLC1A5*(-0.102689) + GPX3*0.096876 + CP*0.083136 + ZFP36*0.077765 + ATF3*0.031323. To evaluate the independent predictive potential of the risk score, we performed univariate and multivariate Cox regression analysis. Univariate Cox regression analysis revealed that the risk score (p<0.001, hazard ratio [HR]=3.453, 95% confidence interval [CI]=2.133-5.591) and clinicopathological parameters, including age (p=0.011, HR=1.022, 95% CI=1.005-1.039), T stage (p=0.007, HR=1.331, 95% CI=1.079-1.641), N stage (p<0.001, HR=1.324, 95% CI=1.140-1.537), M stage (p=0.004, HR=2.340, 95% CI=1.319-4.153), and overall stage (p<0.001, HR=1.558, 95% CI=1.265-1.919), were significantly associated with OS in the TCGA STAD cohorts (Figure 3C). Furthermore, multivariate Cox regression analysis confirmed that the risk score (p<0.001, HR=3.489, 95% CI=2.036-5.980) and age were reliable independent prognostic factors (p<0.05) for predicting the OS of STAD patients in the TCGA database (Figure 3D). According to the median value of risk scores, we subsequently evaluated the prognostic value of this 7-FIRG model. Then, the TCGA training cohort patients were divided into low-risk (174 patients) and high-risk (174 patients) groups. Consistently, high-risk patients showed higher risk scores (Figure 3E) and shorter survival time (Figure 3F), as compared to low-risk individuals. The gene-expression profiles of the prognostic risk genes showed that ZFP36, ATF3, GPX3, SPARC, NOX4, and CP were highly expressed in the high-risk group (Figure 3G). In comparison with the high-risk group, K-M survival analysis revealed a higher survival probability in the low-risk group (p<0.001, HR=1.873, 95% CI=1.350-2.598) (Figure 3H). The receiver operating characteristic (ROC) analysis demonstrated that the area under the ROC curve (AUC) values for the survival probability at 1, 2, 3, 4, and 5 years were 0.637, 0.660, 0.672, 0.687, and 0.715, respectively (Figure 3I). Furthermore, this model was also validated in three different GEO cohorts (GSE84437, GSE62254 and GSE15459), which showed consistent results with the TCGA cohort (Figure S3A-O).

Several prognostic models aimed at predicting survival in STAD patients have been reported in previous studies 27-31. We compared the predictive performance of the 7-FIRG model obtained in this study with five reported models 27-31. To ensure uniformity, gene expression levels involved in each model were extracted from the original matrix of the TCGA STAD dataset. Risk scores for STAD patients were calculated based on the corresponding coefficients for each model. Subsequently, we performed a comparative analysis of the ROC curves, our model based on 7 FIRGs exhibited the optimal AUC value (AUC=0.656) when compared to the other models (Figure S4A). Also, decision curve analyses demonstrated that our model achieved greater net benefits for OS probabilities (Figure S4B). Together, these results demonstrate that the risk model based on these 7 FIRGs presents a reliable accuracy for predicting the OS of GC patients.

### Construction and validation of the nomogram for OS prediction

**Figure 4:**
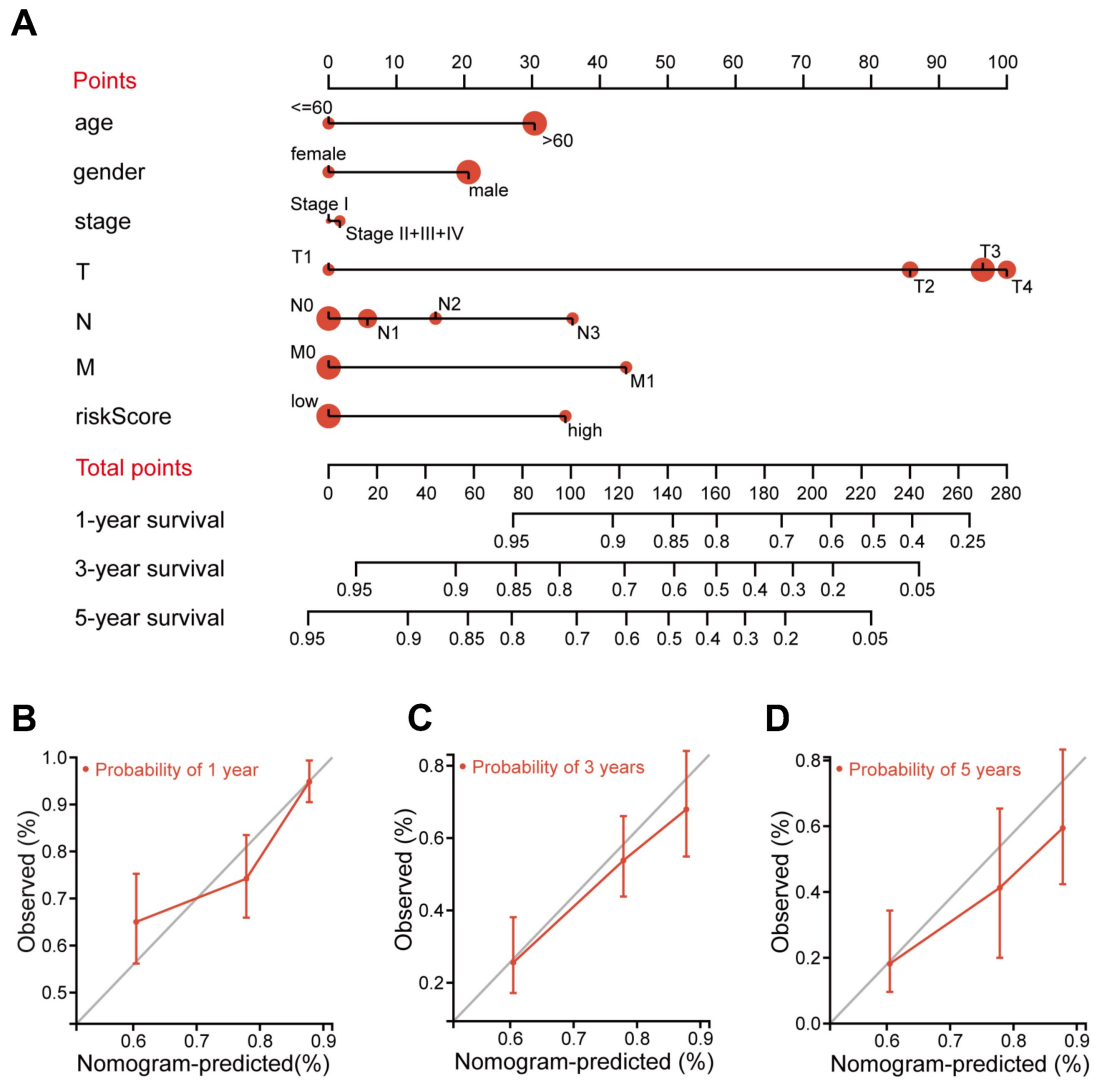
** Construction and validation of the nomogram for OS prediction in the TCGA training cohort.** (A) The nomogram was constructed based on seven independent prognostic factors. (B, C, and D) The calibration plots for the internal validation of the nomogram predicting 1-year (B), 3-year (C), and 5-year (D) OS. The x-axis represents the nomogram predicted survival and the y-axis represents the actual survival.

A nomogram is an efficient tool that integrates multiple risk factors for predicting the OS of cancer patients. Here, we established a nomogram for the prediction of 1-year, 3-year, and 5-year OS in the TCGA STAD cohorts (Figure 4A). Seven independent risk factors, including age, gender, stage, T stage, N stage, M stage and the FIRG signature, were included in this model (Figure 4A). The total points of risk factors indicate their corresponding contribution to the survival probability. The concordance index of our nomogram was 0.682 (p<0.0001, 95% CI=0.636-0.729). We observed that the nomogram-predicted OS matched with the actual observed OS at 1-year, 3-year, and 5-year, as shown by the calibration curves (Figure 4B-D), suggesting that this nomograph is accurate and reliable for the prediction of the OS of STAD patients.

### Exploration of molecular functions and signaling pathways related to FIRGs by GSEA, GO, and KEGG analyses

**Figure 5:**
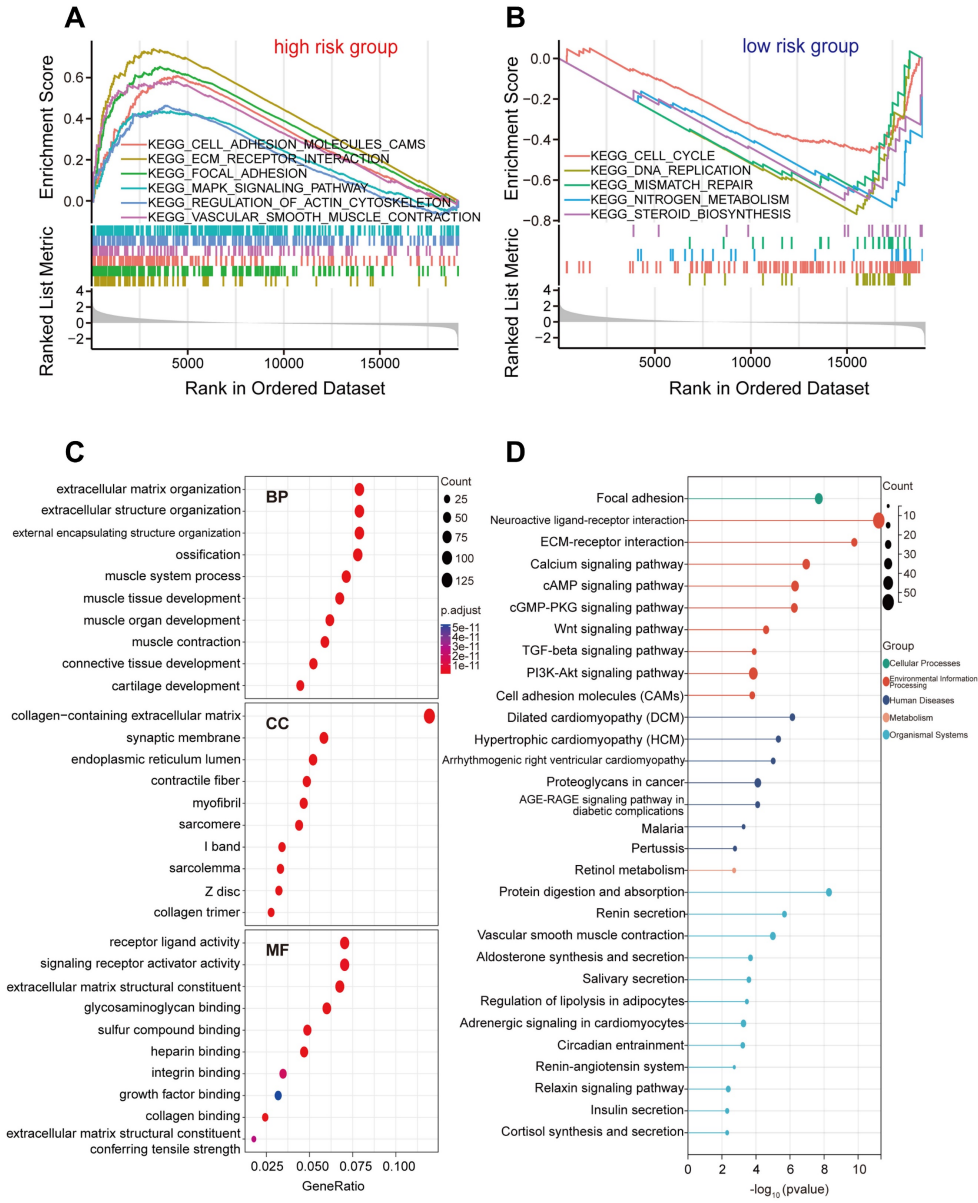
** Biological function and pathway enrichment analysis of high-risk group and low-risk group based on the FIRGs signature.** (A) GSEA of the significantly enriched KEGG terms in the high-risk group. (B) GSEA of the significantly enriched KEGG terms in the low-risk group. (C) Go analysis of the DEGs of the two groups. (D) KEGG pathway analysis of the DEGs of the two groups.

To explore the underlying differences in biological functions related to FIRGs between the high-risk and low-risk groups, GSEA was performed. The details of GSEA results are listed in Supplementary Table 3. Further analysis showed that a total of 36 pathways were significantly enriched in the high-risk group, parts of which were selected and represented, including extracellular matrix (ECM)-receptor interaction (normalized enrichment score [NES]=2.34, p<0.001), focal adhesion (NES=2.20, p<0.001), cell adhesion molecules (CAMs) (NES=2.00, p<0.001), vascular smooth muscle contraction (VSMC) (NES=1.89, p<0.001), regulation of actin cytoskeleton (NES=1.57, p<0.001), mitogen-activated protein kinase (MAPK) signaling pathway (NES=1.49, p<0.001) (Figure 5A). In contrast, 21 pathways were significantly enriched in the low-risk group such as DNA replication (NES=-2.83, p<0.001), nitrogen metabolism (NES=-2.46, p<0.001), cell cycle (NES=-2.20, p<0.001), mismatch repair (NES=-2.18 p<0.001), steroid biosynthesis (NES=-2.15, p<0.001) (Figure 5B). Next, we investigated the differences in biological processes and pathways between the two risk groups based on the FIRG signature. DEGs between the high-risk group and the low-risk group were determined by the cut-off of log_2_|FC|>1 and FDR<0.05. Then, the annotation GO enrichment analysis and KEGG pathway analysis were performed (p<0.05). The GO enrichment analysis obtained 959 BPs, 134 CCs, and 108 MFs (Supplementary Table 4). The top 10 enriched BPs, CCs, and MFs such as extracellular matrix organization (BPs), collagen-containing extracellular matrix (CCs), and receptor ligand activity (MFs) were presented in Figure 5C. The KEGG pathway analysis obtained 61 enriched pathways (Supplementary Table 5), showing that the DEGs were significantly enriched in the signaling pathways of neuroactive ligand-receptor interaction, ECM-receptor interaction, calcium signaling, cyclic adenosine 3', 5'-monophosphate (cAMP), cyclic guanosine 3', 5'-monophosphate (cGMP)-protein kinase G (cGMP-PKG), transforming growth factor (TGF)-beta (TGF-β), phosphatidylinositol-3-kinase (PI3K)-Akt, and CAMs (Figure 5D).

### Analysis of immune status for STAD patients combined with the prognostic signature

**Figure 6:**
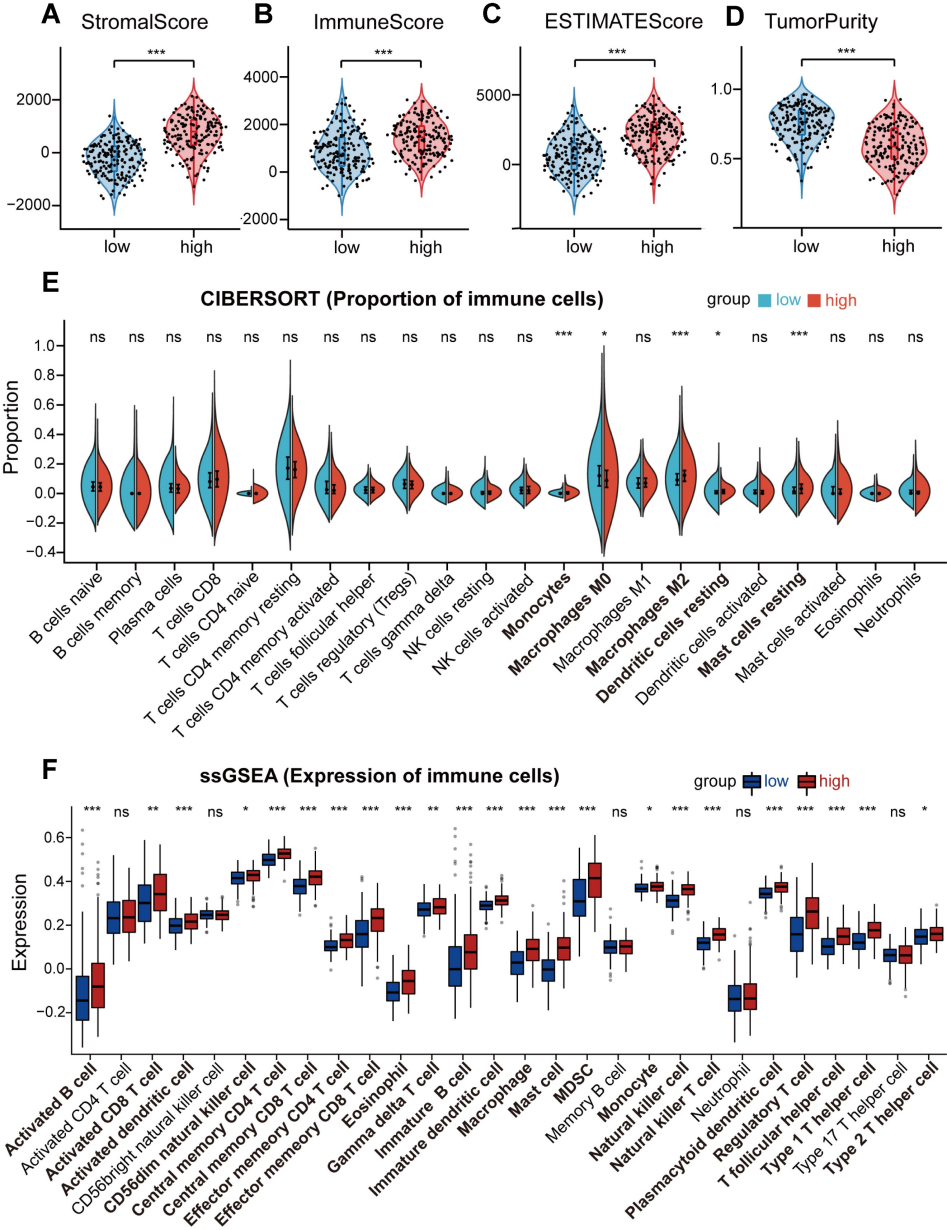
** Analysis of immune status for STAD patients combined with the prognostic signature.** (A, B, C, and D) Comparison of the stromal score (A), immune score (B), ESTIMATE score (C), and tumor purity (D) between the high-risk group and the low-risk group in TCGA STAD cohorts. (E and F) The boxplots for the comparison of the proportion of the 22 immune cells (E) and the expression of the 28 immune cells (F) between the high-risk and low-risk groups. For all, ns: no significant, *p<0.05, **p<0.01, and ***p<0.001.

To investigate the relationship between the risk level in STAD patients and immune cell infiltration, ESTIMATE, CIBERSORT, and ssGSEA analysis were employed in low-risk and high-risk groups. ESTIMATE analysis found that the stromal score, immune score, and estimate score were markedly elevated in the high-risk group (Figures 6A-C), while the tumor purity was remarkably decreased (p<0.001) (Figure 6D). Furthermore, we evaluated the proportion of 22 immune cells in the low-risk and high-risk groups using CIBERSORT analysis, which showed a significant increase in infiltration levels of monocytes (p<0.001), macrophages M2 (p<0.001), resting dendritic cells (p<0.05) as well as resting mast cells (p<0.001) in the high-risk group (Figure 6E), suggesting that risk score was positively correlated with the infiltration levels of monocytes, macrophages M2, resting dendritic cells, and resting mast cells in the high-risk group. In addition, ssGSEA analysis demonstrated that the gene expression levels of 23 immune cell subtypes were significantly upregulated in the high-risk group as compared to the low-risk group (Figure 6F). These results indicate that the high-risk group tends to have a stronger immune infiltration than the low-risk group. Next, to figure out whether immune checkpoints were altered between high-risk and low-risk groups, the expression levels of 33 immune checkpoint molecules were investigated. We found that ADORA2A, BTLA, CD200, CD200R1, CD274, CD276, CD28, CD40, CD44, CD48, CD80, CD86, CTLA4, HAVCR2, IDO1, KIR3DL1, LAG3, LAIR1, NRP1, PDCD1, PDCD1LG2, TIGIT, TNFRSF18, TNFSF14, TNFSF18, and TNFSF4 were significantly elevated in high-risk groups, while LGALS3, PVR, and TNFRSF25 were remarkably decreased in high-risk group (Figure S5A-T and S6A-M).

### Mutations of the prognostic FIRGs in cancer and predictions of the transcription factors (TFs) for the gene signature

**Figure 7:**
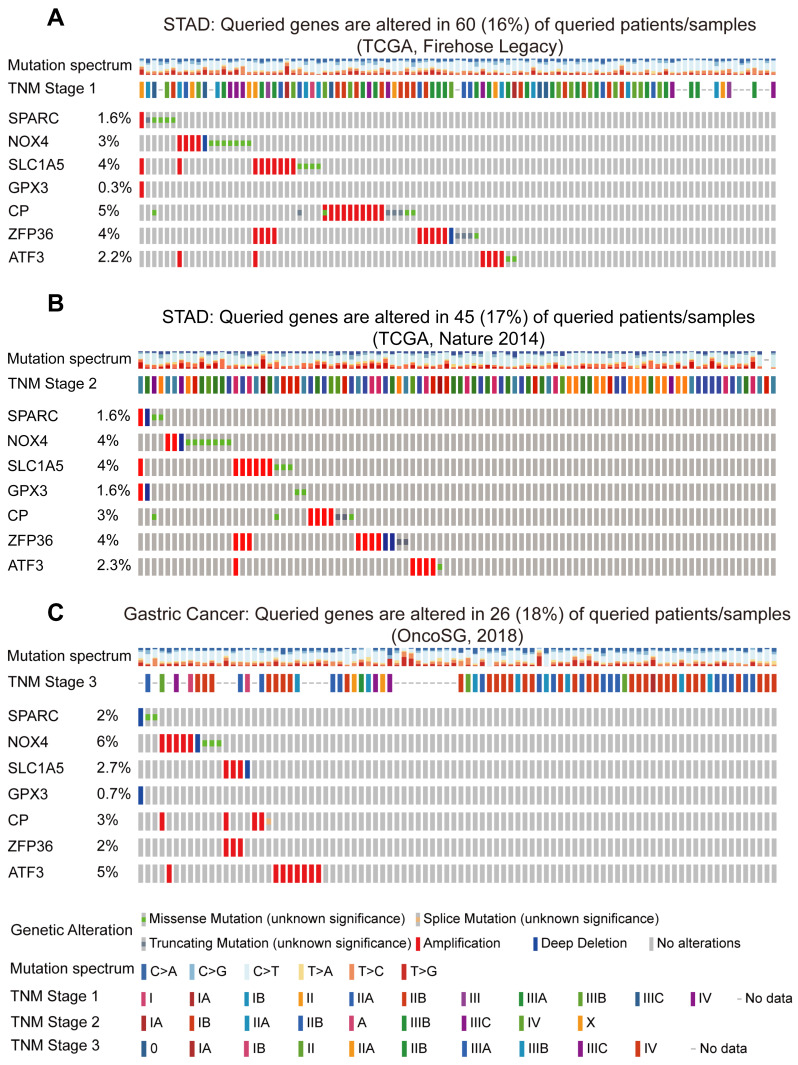
** Mutations of the prognostic FIRGs in multiple groups of gastric cancer.** Genetic alteration of 7 FIRGs in the Gastric Cancer cohorts (TCGA, Firehose Legacy) (A), (TCGA, Nature 2014) (B), (OncoSG, 2018) (C).

To further validate the strong correlation between the prognostic FIRGs and STAD in diverse cohorts, we examined the genetic alterations of STAD patients in the cBioPortal Cancer Genomics (https://www.cbioportal.org/) database. The Firehose Legacy dataset showed that SPARC, NOX4, SLC1A5, GPX3, CP, ZFP36 and ATF3 are mutated in 60 (16%) of the 369 queried patients (Figure 7A). In the dataset of Nature 2014 for STAD, including 258 patients, FIRGs were altered in 45 (17%) patients (Figure 7B). Consistently, in the OncoSG dataset, 26 (18%) of the 147 queried patients displayed mutations of FIRGs (Figure 7C). Moreover, we also investigated the genetic alterations of these prognostic genes in other four different cancer types, including lung adenocarcinoma (LUAD), liver hepatocellular carcinoma (LIHC), breast invasive carcinoma (BRCA), cervical squamous cell carcinoma and endocervical adenocarcinoma (CESC). Intriguingly, frequent mutations were also observed in LUAD (16%), LIHC (13%), BRCA (15%) patients and CESC (18%) patients in the TCGA cohort (Figure S7A-D). These results demonstrate that the mutations of these FIRGs were strongly associated with the initiation and development of cancer. Additionally, we predicted the transcription factors (TFs) of these FIRGs signatures via the ChEA3 website (https://maayanlab.cloud/chea3/). The top 15 TFs were listed in Supplementary Table 6, of which cysteine and serine-rich nuclear protein 1 (*CSRNP1*), FosB proto-oncogene, AP-1 transcription factor subunit (*FOSB*), atonal BHLH transcription factor 8 (*ATOH8*), fos proto-oncogene, and AP-1 transcription factor subunit (*FOS*) presented the significant potential as TFs to modulate FIRGs expression. The protein-protein interaction (PPI) network was also constructed by STRING (https://cn.string-db.org/), demonstrating that there are 22 nodes and 40 edges in the PPI network of FIRGs and TFs (Figure S8A-C).

### *In vivo* validation of prognostic genes expression in gastric cancer

**Figure 8:**
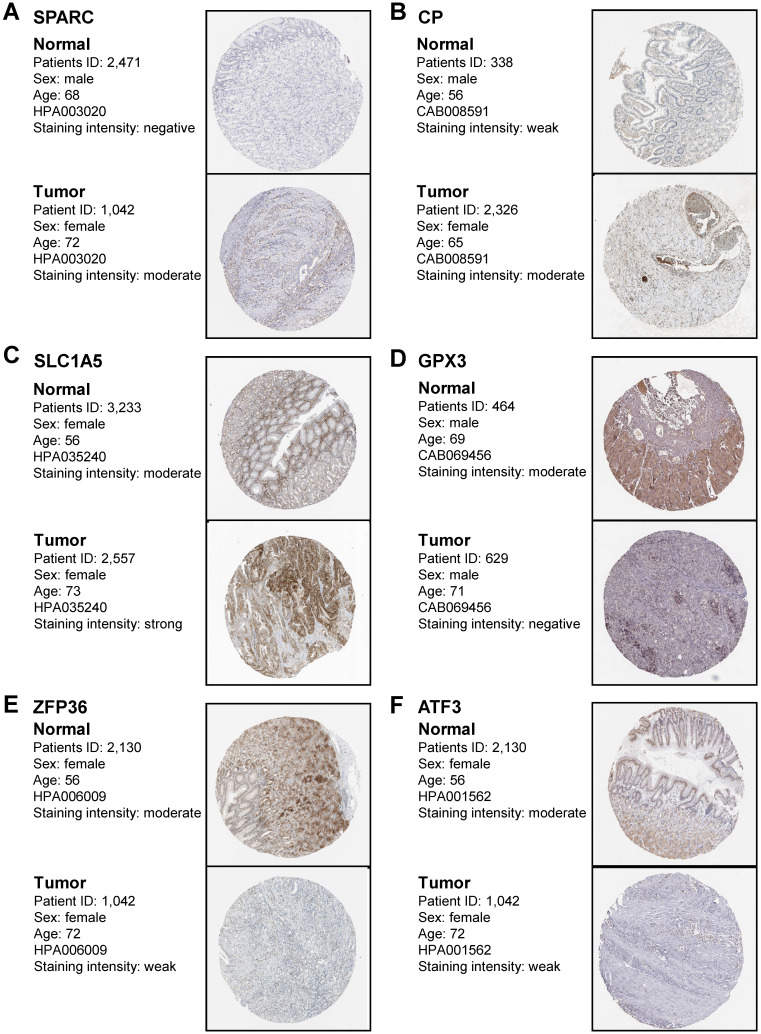
** Immunohistochemistry staining of the prognostic genes in gastric cancer biopsies.** Immunohistochemistry images were obtained from the HPA. The representative IHC image of SPARC protein (A), CP protein (B), SLC1A5 protein (C), GPX3 protein (D), ZFP36 protein (E), and ATF3 protein (F) in normal tissues and gastric cancer tissues. The expression frequency and extent of proteins were defined by staining intensity based on the quantification of the percentage of positively stained cells (none cell indicated as “negative”; <25% positive cells indicated as “weak”; 25%-75% positive cells indicated as “moderate”; >75% positive cells indicated as “strong”).

To confirm that the protein expression levels of FIRGs are closely correlated with STAD occurrence, we performed the immunohistochemical analysis in healthy normal individuals and STAD patients. In accordance with the transcriptional levels, immunohistochemistry (IHC) staining obtained from the Human Protein Atlas (HPA) database indicated that the protein expression levels of SPARC, CP, and SLC1A5 were increased, while GPX3, ZFP36 and ATF3 were reduced in gastric tissue as compared to normal group (Figure 8A-F). These results further provided *in vivo* evidence to validate the reliability of the FIRGs signatures for the prediction of STAD.

### *In vitro* validation of the identified prognostic genes in gastric cancer cells

**Figure 9:**
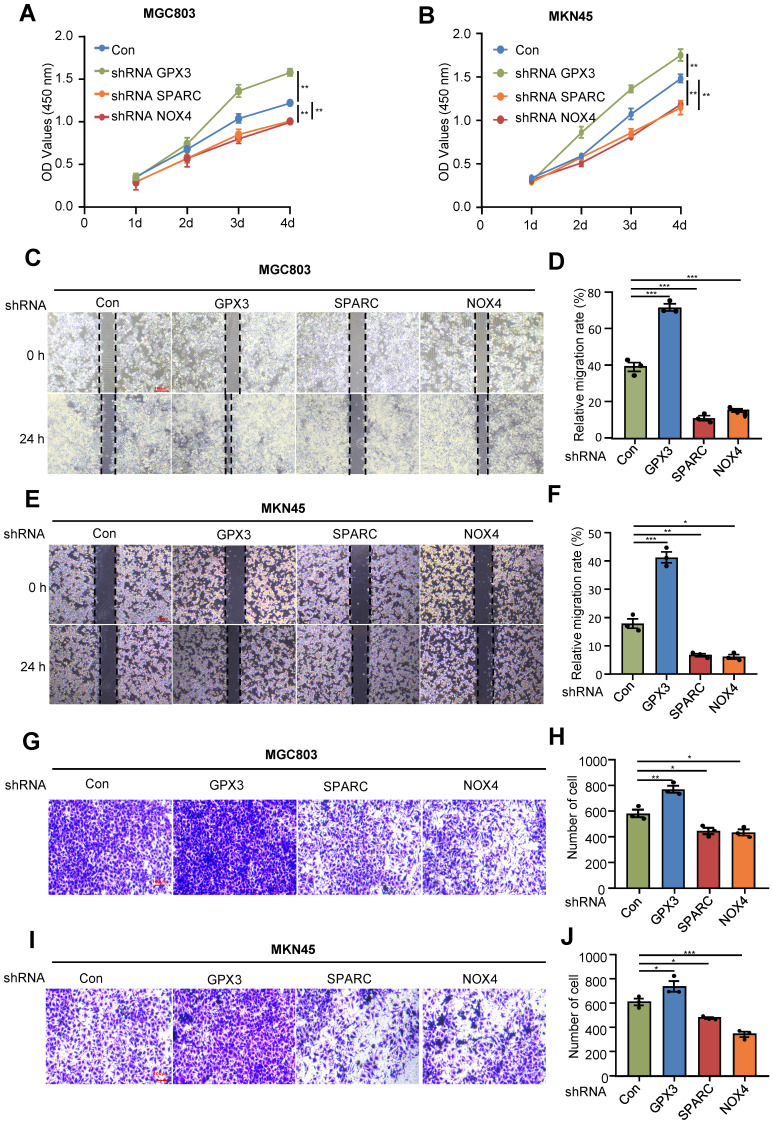
** Analysis of the effects of the prognostic genes in gastric cancer cell lines.** Human gastric cancer cell lines, MGC803 and MKN45 cells were transduced with pLKO.1-TRC shRNA as control (Con) or pLKO.1-GPX3, pLKO.1-SPARC, pLKO.1-NOX4 shRNA to silence GPX3, SPARC, NOX4. Proliferation assay (CCK-8) in MGC803 (A) and MKN45 cells (B). Wound healing assay in MGC803 (C) and MKN45 cells (E). (D)(F) Quantification of the signals in (C)(E). Migration assay in MGC803 (G) and MKN45 cells (I). (H)(J) Quantification of the signals in (G)(I). For all, *p<0.05, **p<0.01, ***p<0.001, Student's t-test. The error bars represent the mean ± SD.

Next, to further validate the roles of FIRGs in the development of gastric cancer, we silenced GPX3, SPARC and NOX4 in gastric cancer cell lines MGC803 and MKN45 to examine the effects on the cell viability, proliferation and migration/invasion. CCK8 and cell wound healing assays showed that GPX3 knockdown significantly enhanced the cell viability and proliferation, while SPARC and NOX4 depletion promoted cell death and reduced cell proliferation capability in MGC803 and MKN45 cell lines (Figure 9A-F). Furthermore, the transwell assay demonstrated that silencing GPX3 markedly induced cell migration/invasion, while SPARC and NOX4 knockdown suppressed the capability of cell migration/invasion in MGC803 and MKN45 cells (Figure 9G-J). These *in vitro* results confirmed that GPX3 served as a protective gene, and SPARC and NOX4 functioned as risk genes for the progression of STAD, which is in line with the *in vivo* findings and prognostic risk evaluation.

## Discussion

With the development of bioinformatics and next-generation sequencing technology, numerous aberrantly expressed oncogenes have been identified and could be employed as prognostic signatures in GC 32-35. Gene signatures based on oncogenes and cell processes, including cell death, metabolism, and immune response, present a variety of advantages in the prognosis prediction of various cancers 36. Ferroptosis, as a newly discovered cell death type, has been demonstrated to have great potential for overcoming the drug resistance mechanism of traditional cancer treatment 37, 38. Indeed, ferroptosis plays a pivotal role in tumor suppression via the inhibition of cell proliferation and migration in STAD cells 37, 39. The immune microenvironment is closely related to the development and progression of STAD 40. Immunotherapy is a novel treatment for STAD that can re-activate the human cellular immune response against tumors and has achieved multiple satisfactory results 41, 42. To our knowledge, the prognostic gene signatures based on FIRGs have not yet been investigated in STAD. The bioinformatics analysis of ferroptosis-related genes (FRGs) in the prognosis of GC has been previously investigated 12, 27, 30, 43, 44, whereas our analysis and model with different approaches based on FIRGs present novel findings.

We also performed somatic mutation and transcription factors prediction to validate the reliability of FIRGs signature for the prediction of GC. Additionally, the expression levels of FIRGs were verified by IHC from the HPA database and the roles of FIRGs in the development of GC were further validated by *in vitro* experiments. In this study, we identified 34 FIRGs in the TCGA STAD training cohorts, 7 of which were significantly associated with the survival probability of STAD patients via Cox univariate analysis and LASSO regression analysis. A new nomogram that integrates multiple risk factors for predicting the OS of STAD patients stratified clinical outcomes in the TCGA cohorts. Immune status analysis indicates that the high-risk STAD group exhibits a stronger immune infiltration than the low-risk group. In this study, we provided *in vivo* and *in vitro* evidence to validate the reliability of the FIRG signature for the prediction of STAD. Taken together, this study suggests that the FIRG signature turned out to be a convincible biomarker for prognosis and might be used in the future for survival risk stratification and personalized management in STAD.

Here, 7 FIRGs, including SPARC, NOX4, SLC1A5, GPX3, CP, ZFP36, and ATF3, were identified to construct a prognostic risk evaluation model via the LASSO Cox regression analysis, which demonstrates a novel gene signature as compared to previous studies 12, 27, 30, 43, 44. Based on the mRNA expression levels of FIRGs and the coefficients of LASSO Cox regression analysis, the risk score for each STAD patient was calculated. Subsequently, we established an evaluation model to validate the prognostic value of these FIRGs. Intriguingly, in the TCGA cohort, high-risk patients showed higher risk scores and shorter survival days as compared to low-risk individuals. K-M survival analysis indicated a higher survival probability and longer survival days in the low-risk group in comparison with the high-risk group. Furthermore, this model showed consistent results with the TCGA cohort in three independent GEO cohorts. Moreover, the genetic alterations of FIRGs in STAD patients from the Firehose Legacy dataset, Nature 2014 dataset, and OncoSG dataset further validate the strong correlation between the prognostic FIRGs and STAD occurrence in diverse cohorts. In addition, a nomogram that integrates age, gender, stage, T stage, N stage, M stage and the FIRG signature for the prediction of 1-year, 3-year and 5-year OS in the TCGA STAD cohorts was also established. The calibration curves of the nomogram showed the predicted OS kept in line with the actual observed OS at 1-year, 3-year, and 5-year, suggesting that this nomograph is accurate and reliable for the prediction of the OS of STAD patients. These results demonstrate that the risk model based on these 7 FIRGs presents a reliable accuracy for predicting the OS of GC patients. Consistently, Song et al. demonstrated that a risk model based on ferroptosis-related genes (FRGs) was also able to accurately predict the prognosis in GC 43. A ceRNA network based on FRGs in the prognostic model also showed excellent potential in predicting GC prognosis 29. The identification of the FIRG signature shows clinical implications, as it is significantly related to the outcomes of STAD patients. Gene-targeted therapy is a novel treatment that is effective in some gastric cancer patients with gene mutations. Compared to traditional treatments, patients at high risk might benefit from innovative therapies, such as DNA- and RNA-based therapeutics, whereas those with low-risk gene signatures could temporarily postpone undergoing those methods. The prognostic model may help with patient categorization, enable specific treatment strategies for STAD patients in clinical settings, and eventually be beneficial in lowering mortality.

To elucidate the potential mechanism of FIRGs modulating the pathogenesis of GC, the biological functions and signaling pathways of FIRGs were performed in the high-risk and low-risk groups. GSEA and GO enrichment analysis demonstrated that the FIRGs were involved in the mediation of ECM, CAMs, actin cytoskeleton, and MAPK in the high-risk group. Whereas, in the low-risk group, FIRGs modulated the cell cycle, DNA replication, mismatch repair, nitrogen metabolism, and steroid biosynthesis. These results suggest that FIRGs exert different functions in the normal and neoplastic gastric tissues. KEGG enrichment analysis showed that DEGs were mainly enriched in the signaling pathways of ECM-receptor interaction, calcium signaling, CAMs, cGMP-PKG, TGF-β, and PI3K-Akt. ECM, a dynamic and organized tissue structure, has been revealed to be an accomplice in promoting the development and progression of GC via the regulation of ferroptosis 45, 46. CAMs also have been implicated in the development and progression of GC 47. It's well-accepted that cell adhesion molecules, including receptors of the immunoglobulin superfamily and integrins, trigger tumor growth and metastases via mediating the immune cell-mediated inflammation, immune cell infiltration, immune responses as well as tumor immune microenvironment 48. Actin cytoskeleton also contributes to the development and metastasis of GC through actin cytoskeleton dynamic rearrangement 49. Studies of actin-related primary immunodeficiencies have revealed that the actin cytoskeleton plays a crucial role in the regulation of immune system function, including immune cells proliferation, differentiation, recruitment, migration, intracellular signaling transduction, and activation of both innate and adaptive immune responses 50. However, whether cell adhesion molecules and actin cytoskeleton contribute to GC development still requires further investigation. Concerning MAPK, a large body of studies has demonstrated that it is involved in GC proliferation, invasion, migration, and metastasis by mediating ferroptosis processes and the immune system through the complex signaling pathways 51-53. Also, cGMP-PKG, TGF-β and PI3K-Akt have been implicated with the modulation of ferroptosis and immunity, which contributes to the development of GC 54-57. Mechanistically, these results disclose that FIRGs may contribute to the pathogenesis of GC by regulating ferroptosis and the immune system through enriched pathways. Additionally, we predicted the TFs of the FIRG signature via the ChEA3, and found that CSRNP1, FOSB, ATOH8 and FOS were TF candidates to modulate FIRGs expression. The PPI network was also constructed by STRING, demonstrating that there are 22 edges and 40 nodes in the PPI network of FIRGs and TFs.

Immunotherapy is revolutionizing cancer therapy, and emerging evidence has clarified that targeting our immune system presents great efficacy for protecting against cancer 58. The TME comprises various types of cells, such as immune cells, fibroblasts, myofibroblasts, neuroendocrine cells, and stromal cells 59. Immune cells infiltrating into TME to interact with other immune cells remarkably contribute to the development, progression and malignancy of GC 60. To explore the roles of immune cell infiltration and ferroptosis in GC, ESTIMATE, CIBERSORT, and ssGSEA analysis were employed in low-risk and high-risk groups. Compared with the low-risk group, GC patients in the high-risk group had higher ESTIMATE score, stromal score and immune score, while the tumor purity was significantly reduced. The increase of infiltrated immune cells accompanies stroma activation, which could prevent the entry of T cells from the tumor parenchyma to the peritumoral stroma 61, thereby causing a poor prognosis in the high-risk group 62. Furthermore, we found a significant increase in infiltration levels of monocytes, macrophages M2, resting dendritic cells as well as resting mast cells in the high-risk group evaluated by CIBERSORT analysis. Tumor-associated macrophages of the M2 phenotype have been found to promote tumor proliferation and metastasis and to be associated with a poor prognosis in GC patients 63, 64. Besides, ssGSEA analysis demonstrated that the gene expression levels of immune cells were significantly upregulated in the high-risk group as compared to the low-risk group. These results prompt us to speculate that the elevated infiltrated immune cells in TEM accelerate the GC progression, in turn causing the poor prognosis in the high-risk group. As abnormal TME induces immunosuppression to compromise cancer immunotherapy, identifying the immune cells in the TME may be beneficial to predict immunotherapy responses and improve antitumor activity 65. The immune checkpoints expressed on tumor cells protect cancer cells from damage by local immune responses 66. In GC, it is still unclear how many immune checkpoints are expressed and whether they are useful for predicting the prognosis of GC patients. In comparison with previous investigations 12, 27, 30, 43, 44, for the first time, we found that the expression levels of 26 immune checkpoint molecules (ADORA2A, BTLA, CD200, CD200R1, CD274, CD276, CD28, CD40, CD44, CD48, CD80, CD86, CTLA4, HAVCR2, IDO1, KIR3DL1, LAG3, LAIR1, NRP1, PDCD1, PDCD1LG2, TIGIT, TNFRSF18, TNFSF14, TNFSF18, and TNFSF4) were significantly elevated in the high-risk groups. Among them, the roles of CD274, CD276, CD28, CD44, CD48, CD86, HAVCR2, KIR3DL1, LAIR1, TNFSF14, and TNFSF4 in modulating GC have not been elucidated, which still awaits further intensive investigations.

Finally, our experimental work revealed that the silence of SPARC and NOX4 inhibited GC migration and proliferation, whereas silencing GPX3 promoted GC migration and proliferation, which is in accordance with the previous studies. For instance, a higher expression level of the SPARC mRNA was observed in cancer tissue as compared to adjacent normal mucosa 67. The 3- and 5-year survival of patients with lower expression of SPARC was significantly better than those with a higher expression 67, 68. Therefore, SPARC is associated with GC progression and poor survival of patients, which could be useful markers to predict tumor progression 69. Whereas, the role of SPARC in the regulation of ferroptosis still remains elusive. Nicotinamide adenine dinucleotide phosphate (NADPH) oxidase 4 (NOX4), a subunit of the NOX complex, has been demonstrated to drive reactive oxygen species generation, in turn contributing to cell proliferation and apoptosis of gastric cancer cells via activation of the GLI1 pathway 70. A study showed that NOX4 expression strongly correlated with tumor size, lymphatic metastasis, vascular invasion and a poor prognosis in GC patients, and suppressed cancer-associated fibroblasts-mediated immunotherapy 71. In Alzheimer's disease, NOX4 promotes the ferroptosis of astrocytes by oxidative stress-induced lipid peroxidation via the impairment of mitochondrial metabolism 72. SLC1A5, a suppressor gene against ferroptosis, was found to be upregulated in GC cell lines 73. Knockdown of SLC1A5 in GCs suppressed cell proliferation, invasion as well as migration partly through the inactivated mTOR/p-70S6K1 signaling pathway *in vitro* 74. CP can induce human GC apoptosis via activation of the ERK1/2 signaling pathway 75. Furthermore, the inhibition of CP loop can promote ferroptosis and radiosensitivity by disrupting Cu-Fe homeostasis, demonstrating that CP may be a new target and treatment strategy for overcoming tumor radioresistance 76. GPX3 has been reported to inhibit GC migration and invasion by targeting NFкB/Wnt5a/JNK signaling 77. Accordingly, GPX3 is required for the tumor-polarized immunosuppressive function of AT2 cells 78. ATF3, a common stress sensor, can inhibit GC proliferation, colony formation, cell migration and invasion and tumorigenesis in a mouse xenograft model 79. Moreover, ATF3 can sensitize GC cells to cisplatin by induction of ferroptosis via blocking Nrf2/Keap1/xCT signaling, supporting a promising therapeutic approach for overcoming chemoresistance in GC 80. The gene ZFP36 also shows tumor-specific functions, but its biological roles in GC remain largely unknown. A study reported that ZFP36 is correlated with the impairment of erastin- or sorafenib-induced HSC ferroptosis 81. Moreover, ZFP36 RBPs play a critical role in restraining T cell expansion and effector functions, and suggest ZFP36 inhibition as a strategy to enhance immune-based therapies 82. Taken together, these results strongly support the notion that the identified FIRGs can predict the development and progression of GC.

## Conclusion

In summary, we identified a reliable prognostic FIRG signature based on the analysis of ferroptosis- and immunity-related genes in different training cohorts. The risk model based on FIRGs is reliable and accurate in predicting the prognosis of GC. This study may provide new insights into the molecular mechanism of how ferroptosis and immunity contribute to the pathogenesis and prognosis of GC, and may uncover novel prognostic strategies and therapeutical targets for GC therapy.

## Supplementary Material

Supplementary figures and tables.

## Abbreviations

GC: Gastric cancer
FIRGs: ferroptosis- and immunity-related genes
STAD: stomach adenocarcinoma
TCGA: The Cancer Genome Atlas
LASSO: least absolute shrinkage and selection operator
ICD: immunogenic cell death
OS: overall survival
GO: Gene Ontology
KEGG: Kyoto Encyclopedia of Genes and Genomes
TME: tumor microenvironment
ssGSEA: single-sample gene set enrichment analysis
K-M: Kaplan-Meier
DEGs: differentially expressed genes
AGE: advanced glycation end product
RAGE: receptor for AGE
BPs: biological processes
CCs: component categories
MFs: molecular functions
ROC: receiver operating characteristic
AUC: area under the ROC curve
ECM: extracellular matrix
CAMs: cell adhesion molecules
VSMC: vascular smooth muscle contraction
MAPK: mitogen-activated protein kinase
cAMP: cyclic adenosine 3', 5'-monophosphate
cGMP: cyclic guanosine 3', 5' -monophosphate
cGMP-PKG: cGMP-protein kinase G
TGF-β: transforming growth factor-beta
PI3K: phosphatidylinositol-3-kinase
CAMs: cell adhesion molecules
LUAD: lung adenocarcinoma
LIHC: liver hepatocellular carcinoma
BRCA: breast invasive carcinoma
CESC: endocervical adenocarcinoma
TFs: transcription factors
CSRNP1: cysteine and serine-rich nuclear protein 1
IHC: immunohistochemistry
HPA: Human Protein Atlas
FRGs: ferroptosis-related genes
