# Supplementary material for: Identification of A Novel Gene Signature Combining Ferroptosis- and Immunity-Related Genes for Prognostic Prediction, Immunotherapy and Potential Therapeutic Targets in Gastric Cancer
Source: J Cancer. 2023 Oct 16;14(18):3457–76. doi: 10.7150/jca.87223 (PMC10647194; doi:10.7150/jca.87223)
Supplement: Supplementary file 1 — Supplementary figures and tables. [file jcav14p3457s1.pdf]

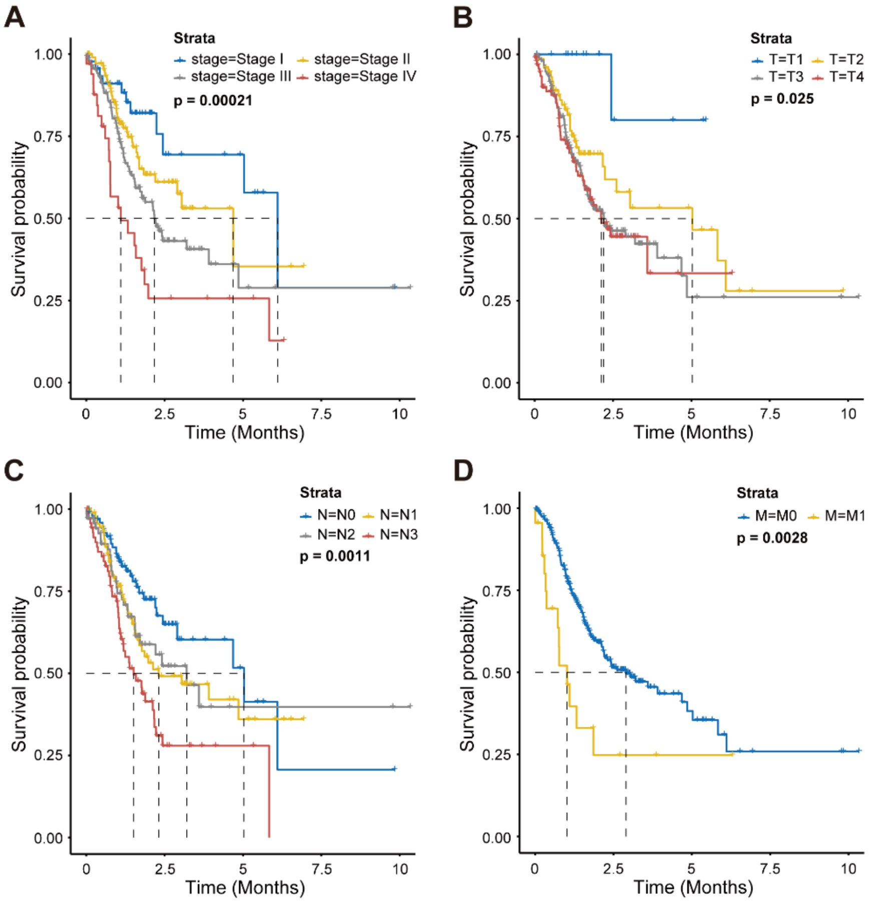

Figure S1. Kaplan-Meier survival curves regarding the clinical features including stage (A), T stage (B), N stage (C), and M stage (D) in the TCGA STAD cohort.

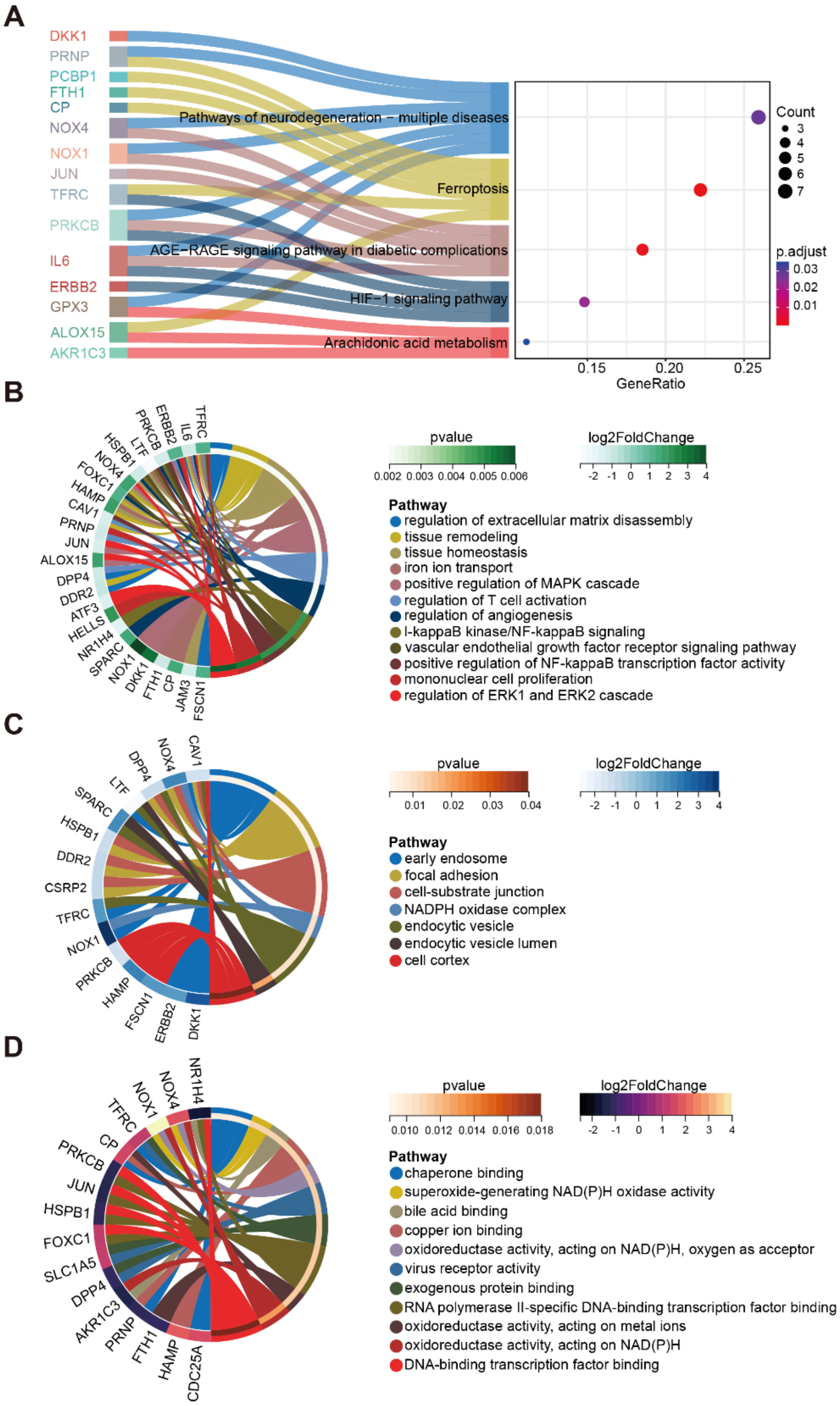

Figure S2. Functional analysis of the 34 OS-related FIRGs in TCGA cohort

(A) KEGG analysis of the FIRGs. (B-D) GO analysis of the FIRGs, including the biological processes (B), the component categories (C), and the molecular functions (D).

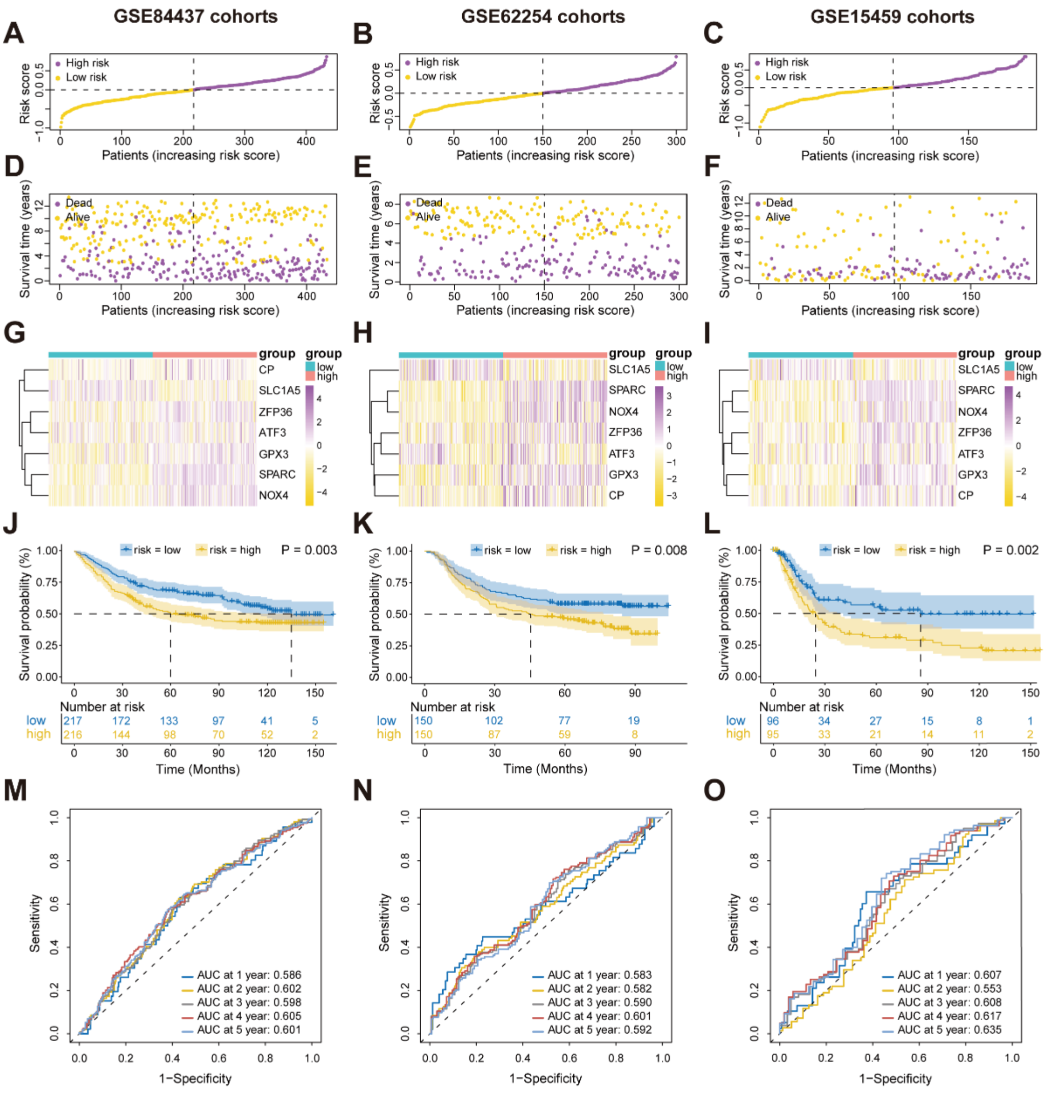

Figure S3. Validation of the FIRG signature model in the GEO cohorts

(A, B, C, D, E, and F) The distributions of the risk score, survival time, and status of patients in GEO84437 validation cohorts (A and D), GEO62254 validation cohorts (B and E), and GEO15459 validation cohorts (C and F). The dotted lines indicate the optimal cut-off value between the low- and high-risk groups. (G, H, and I) Heatmap of the gene-expression profiles of the FIRG signature in GEO84437 validation cohorts (G), GEO62254 validation cohorts (H), and GEO15459 validation cohorts (I). (J, K, and L) Kaplan-Meier curves of the gene signature in GEO84437 validation cohorts (J), GEO62254 validation cohorts(K), and GEO15459 validation cohorts (L). (M, N, and O) The time-dependent ROC curves of the prognostic gene signature in GEO84437 validation cohorts (M), GEO62254 validation cohorts(N), and GEO15459 validation cohorts(O).

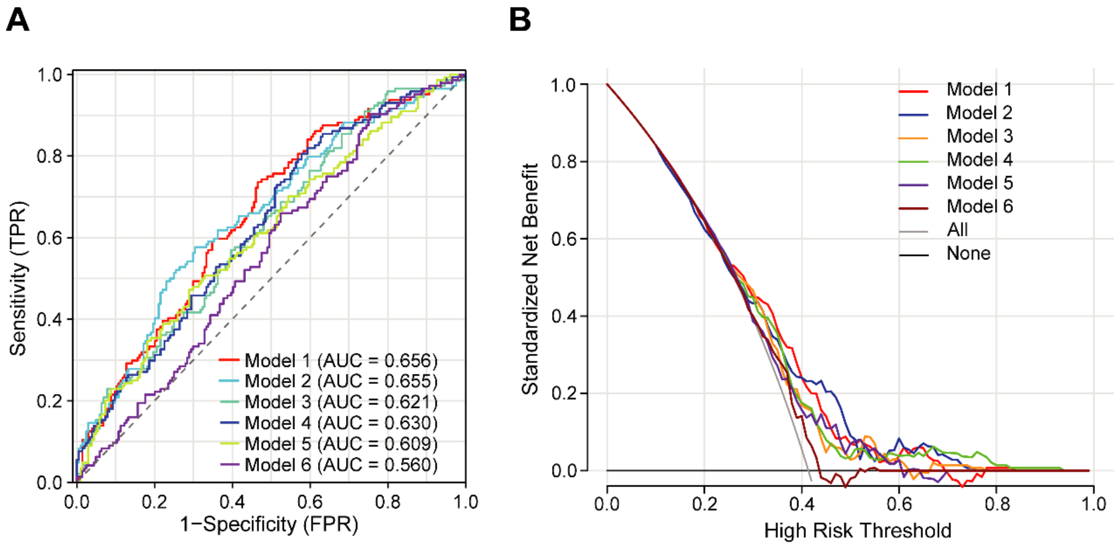

Figure S4. The statistical comparison of the FIRGs prognostic risk evaluation model with previous models.

ROC curves (A) and DCA profiles (B) compared the performance of the 7-FIRGs model to previous signatures in the TCGA STAD dataset. Model 1 (7-FIRGs model), Model 2 (Deng et al.) [27], Model 3 (Shao et al.) [30], Model 4 (Liu et al.) [29], Model 5 (Wang et al.) [31], and Model 6 (Liu et al.) [28].

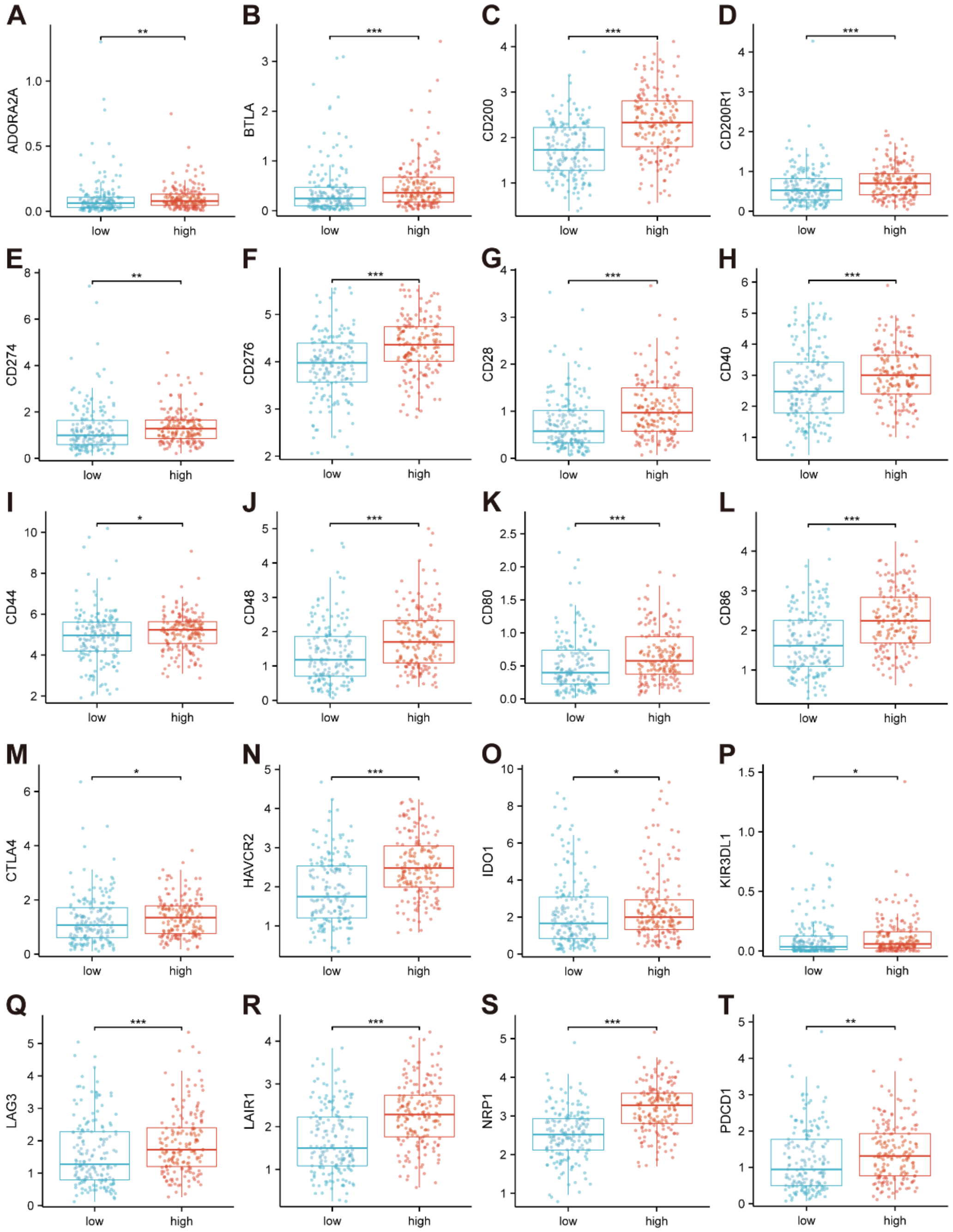

Figure S5. The expression of immune checkpoints between high-risk groups and low-risk groups.

Differential expression of ADORA2A (A), BTLA(B), CD200 (C), CD200R1 (D), CD271 (E), CD276 (F), CD28 (G), CD40 (H), CD44 (I), CD48 (J), CD80 (K), CD86 (L), CTLA4 (M), HAVCR2 (N), IDO1 (O), KIR3DL1 (P), LAG3 (Q), LAIR1 (R), NRP1 (S) and PDCD1 (T) in high-risk groups and low-risk groups. For all, ns: no significant, *p<0.05, **p<0.01 and ***p<0.001.

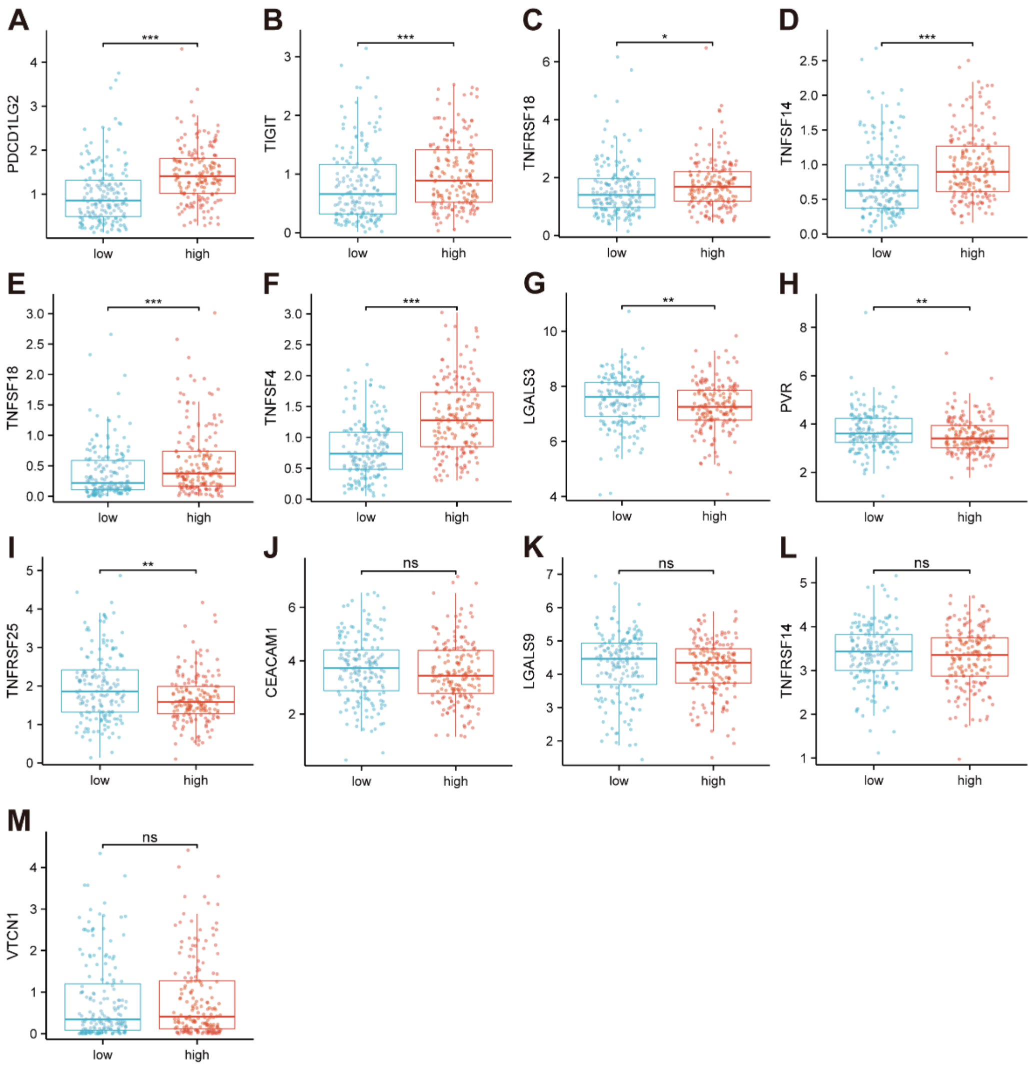

Figure S6. The expression of immune checkpoints between high-risk groups and low-risk groups.

Differential expression of PDCD1LG2 (A), TIGIT(B), TNFRSF18 (C), TNFSF14 (D), TNFSF18 (E), TNFSF4 (F), LGALS3 (G), PVR (H), TNFRSF25 (I), CEACAM1 (J), LGALS9 (K), TNFRSF14 (L) and VTCN1 (M) in high-risk groups and low-risk groups. For all, ns: no significant, *p<0.05, **p<0.01 and ***p<0.001.

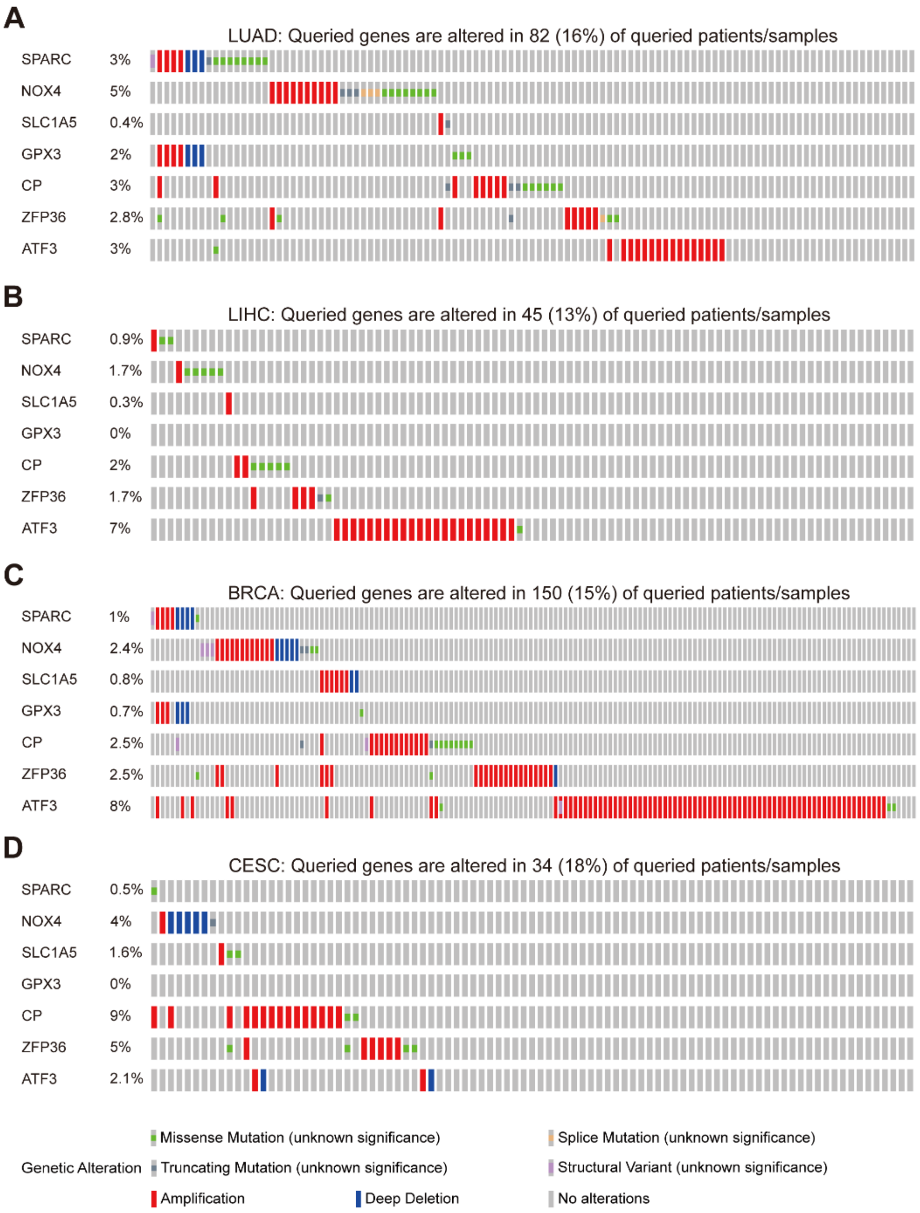

Figure S7. Genetic alteration profiles of the prognostic genes in the LUAD (A), LIHC (B), and BRCA (C) RNA-seq dataset cohorts (TCGA, PanCancer Atlas), and CESC (D) RNA-seq dataset cohorts (TCGA, Firehose Legacy).

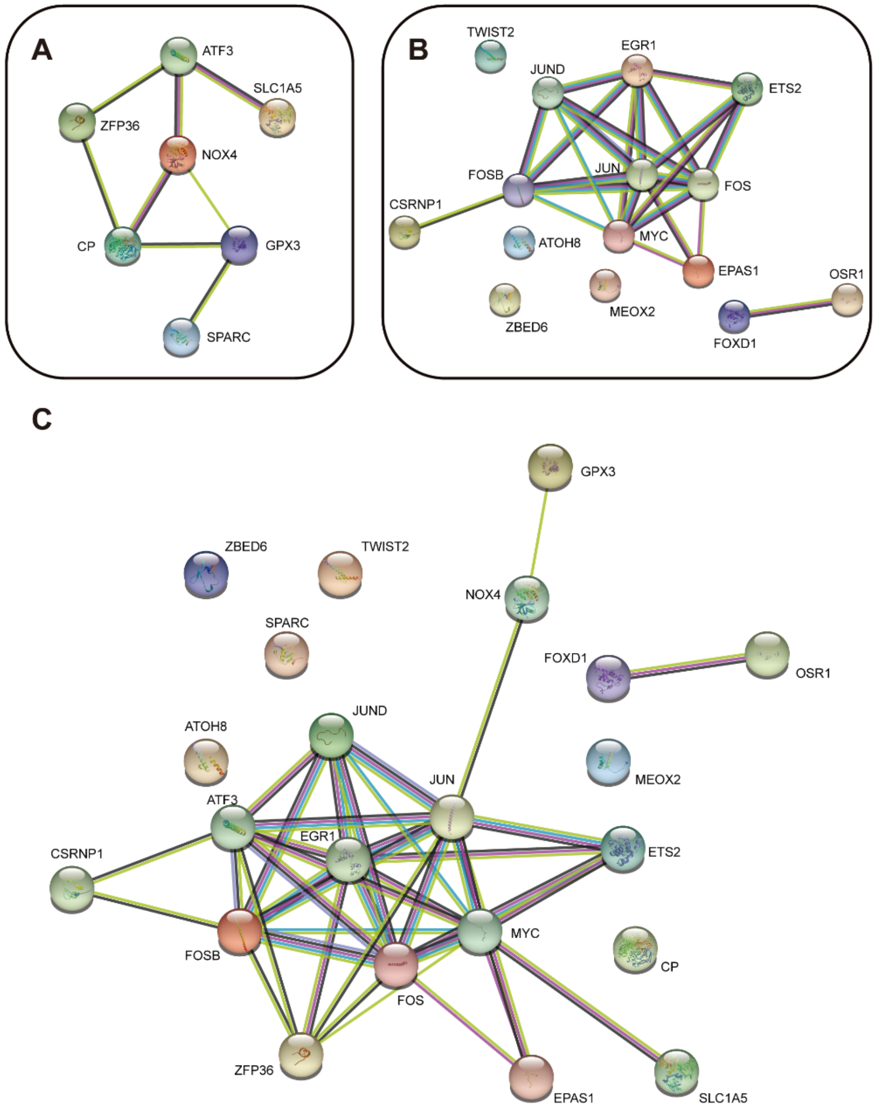

Figure S8. Construction of protein-protein interaction (PPI) network
(A) PPI network of 7 FIRGs. (B) PPI network of 15 transcription factors (TFs) predicted. (C) PPI network of both FIRGs and TFs.

Table S1. Characteristics of patients with stomach cancer in this study.

Variables	TCGA-STAD	GSE84437	GSE62254	GSE15459
Cases	n = 348	n = 433	n = 300	n = 191
Follow up status				
Alive	204	224	148	96
Dead	144	209	152	95
Age				
>60	232	239	183	-
≤60	116	194	117	-
Gender				
Male	225	296	199	124
Female	123	137	101	67
Stage				
Stage I	46	21	30	31
Stage II	110	138	96	29
Stage III	144	274	95	72
Stage IV	34	0	77	59
Unknown	14	0	2	0
T classification				
T1	16	11	0	-
T2	74	38	186	-
T3	159	92	91	-
T4	95	292	21	-
Unknown	4	0	2	-
N classification				
N0	103	80	38	-
N1	92	188	131	-
N2	71	132	80	-
N3	71	33	51	-
Unknown	11	0	0	-
M classification				
M0	311	433	273	-
M1	22	0	27	-
Unknown	15	0	0	-

Table S2. The function of the genes in the prognostic gene signature

Number	Gene Symbol	Full Name	Gene Ontology	Function	Risk Coefficient
1	SPARC	Secreted Protein Acidic and Cysteine Rich	calcium ion binding and extracellular matrix binding	Binding and Uptake of Ligands by Scavenger Receptors and Extracellular matrix organization	0.119965
2	NOX4	NADPH Oxidase 4	nucleotide binding and heme binding	Disease and Blood-Brain Barrier and Immune Cell Transmigration: VCAM-1/CD106 Signaling	0.113284
3	SLC1A5	Solute Carrier Family 1 Member 5	signaling receptor activity and amino acid transmembrane transporter activity	Glucose / Energy Metabolism and RAC1 GTPase cycle	-0.102689
4	GPX3	Glutathione Peroxidase 3	transcription factor binding and selenium binding	Detoxification of Reactive Oxygen Species and Nuclear receptors meta-pathway	0.096876
5	CP	Ceruloplasmin	oxidoreductase activity and copper ion binding	Glucose / Energy Metabolism and Disease	0.083136
6	ZFP36	ZFP36 Ring Finger Protein	RNA binding and enzyme binding	Regulation of activated PAK-2p34 by proteasome mediated degradation and PDGFR-beta signaling pathway	0.077765
7	ATF3	Activating Transcription Factor 3	DNA-binding transcription factor activity and sequence-specific DNA binding	Glucose / Energy Metabolism and Tacrolimus/Cyclosporine Pathway, Pharmacodynamics	0.031323

Table S3. The list of KEGG pathways enriched in GSEA

	Description	setSize	enrichmentScore	NES	p-value	p.adjust	qvalue	rank	core_enrichment
1	KEGG_ECM_RECEPTOR_INTERACTION	83	0.739132846	2.340931557	1.00E-10	3.72E-09	2.32E-09	3216	tags=65%, list=17%, signal=54%
2	KEGG_RENIN_ANGIOTENSIN_SYSTEM	17	0.688551634	1.689132473	0.00380948	0.015075816	0.009385058	2272	tags=41%, list=12%, signal=36%
3	KEGG_HYPERTROPHIC_CARDIOMYOPATHY_HCM	83	0.675628516	2.139804937	1.00E-10	3.72E-09	2.32E-09	3343	tags=47%, list=18%, signal=39%
4	KEGG_GRAFT_VERSUS_HOST_DISEASE	37	0.656670817	1.885007923	0.00019648	0.001142037	0.000710946	4833	tags=65%, list=25%, signal=49%
5	KEGG_DILATED_CARDIOMYOPATHY	90	0.654791568	2.103339758	8.23E-10	2.19E-08	1.36E-08	4181	tags=56%, list=22%, signal=44%
6	KEGG_FOCAL_ADHESION	199	0.652864653	2.199713744	1.00E-10	3.72E-09	2.32E-09	3553	tags=46%, list=19%, signal=38%
7	KEGG_HEMATOPOIETIC_CELL_LINEAGE	84	0.640871243	2.035803709	1.86E-08	2.88E-07	1.79E-07	4791	tags=67%, list=25%, signal=50%
8	KEGG_ASTHMA	28	0.635690503	1.74076179	0.005416093	0.020147866	0.012542531	4380	tags=68%, list=23%, signal=52%

9	KEGG_ARRHYTHMOGENIC_RIGHT_VENTRICULAR_CARDIOMYOPATHY_ARVC	74	0.627623928	1.963119744	3.90E-07	4.84E-06	3.01E-06	3343	tags=47%, list=18%, signal=39%
10	KEGG_COMPLEMENT_AND_COAGULATION_CASCADES	69	0.624614819	1.939059218	4.51E-06	4.57E-05	2.84E-05	3604	tags=55%, list=19%, signal=45%
11	KEGG_LEISHMANIA_INFECTION	69	0.624003022	1.937159948	4.67E-06	4.57E-05	2.84E-05	4380	tags=52%, list=23%, signal=40%
12	KEGG_CELL_ADHESION_MOLECULES_CAMS	130	0.607955672	1.995965835	6.92E-10	2.15E-08	1.34E-08	4474	tags=59%, list=23%, signal=46%
13	KEGG_ALLOGRAFT_REJECTION	35	0.605891947	1.714643876	0.002234522	0.009445935	0.005880322	4833	tags=60%, list=25%, signal=45%
14	KEGG_PRION_DISEASES	35	0.58692984	1.660982062	0.004491113	0.017403064	0.010833826	2721	tags=37%, list=14%, signal=32%
15	KEGG_VASCULAR_SMOOTH_MUSCLE_CONTRACTION	115	0.58285805	1.889667615	2.83E-07	3.76E-06	2.34E-06	4157	tags=41%, list=22%, signal=32%
16	KEGG_ADIPOCYTOKINE_SIGNALING_PATHWAY	66	0.579039811	1.787181744	8.93E-05	0.000692162	0.000430888	2672	tags=24%, list=14%, signal=21%

17	KEGG_TYPE_I_DIABETES_MELLITUS	41	0.561067151	1.624629448	0.005116967	0.019423588	0.012091651	4833	tags=59%, list=25%, signal=44%
18	KEGG_NEUROACTIVE_LIGAND_RECEPTOR_INTERACTION	272	0.551691384	1.878267569	1.00E-10	3.72E-09	2.32E-09	3370	tags=44%, list=18%, signal=37%
19	KEGG_GAP_JUNCTION	89	0.542472504	1.738440063	0.00015397	0.001101479	0.000685697	4248	tags=37%, list=22%, signal=29%
20	KEGG_CALCIUM_SIGNALING_PATHWAY	177	0.534213954	1.785786797	9.91E-08	1.42E-06	8.83E-07	2719	tags=34%, list=14%, signal=30%
21	KEGG_ALDOSTERONE_REGULATED_SODIUM_REABSORPTION	42	0.532342892	1.546963443	0.010748713	0.037023344	0.023047922	3669	tags=33%, list=19%, signal=27%
22	KEGG_CYTOKINE_CYTOKINE_RECEPTOR_INTERACTION	261	0.528453421	1.795749224	3.83E-09	7.13E-08	4.44E-08	4079	tags=49%, list=21%, signal=39%
23	KEGG_AXON_GUIDANCE	129	0.51956056	1.705228945	3.69E-05	0.000298663	0.000185925	3526	tags=33%, list=18%, signal=27%
24	KEGG_VIRAL_MYOCARDITIS	68	0.514707794	1.59566012	0.003564989	0.014414956	0.008973657	4406	tags=43%, list=23%, signal=33%

25	KEGG_CHEMOKINE_SIGNALING_PATHWAY	186	0.510895004	1.709277222	1.08E-06	1.26E-05	7.84E-06	4994	tags=49%, list=26%, signal=37%
26	KEGG_LONG_TERM_DEPRESSION	70	0.509290379	1.583291269	0.005897954	0.021510184	0.013390607	3441	tags=29%, list=18%, signal=24%
27	KEGG_TGF_BETA_SIGNALING_PATHWAY	85	0.506760918	1.612770347	0.001694057	0.007327781	0.00456172	3130	tags=29%, list=16%, signal=25%
28	KEGG_HEDGEHOG_SIGNALING_PATHWAY	56	0.502549883	1.521975676	0.011035268	0.037319269	0.023232143	2542	tags=30%, list=13%, signal=26%
29	KEGG_TOLL_LIKE_RECEPTOR_SIGNALING_PATHWAY	102	0.492645237	1.589281506	0.001304976	0.006068138	0.003777562	4407	tags=33%, list=23%, signal=26%
30	KEGG_PPAR_SIGNALING_PATHWAY	69	0.489262466	1.518870296	0.011401029	0.037867702	0.023573556	4221	tags=35%, list=22%, signal=27%
31	KEGG_LEUKOCYTE_TRANSENDOTHELIAL_MIGRATION	115	0.483247903	1.566724372	0.001383933	0.006128845	0.003815353	4812	tags=43%, list=25%, signal=32%
32	KEGG_REGULATION_OF_ACTIN_CYTOSKELETON	212	0.463967374	1.568160763	0.00011747	0.000873974	0.00054407	3845	tags=33%, list=20%, signal=27%

33	KEGG_MELANOGENESIS	101	0.45568649	1.470538473	0.009659865	0.033900658	0.021103975	4319	tags=37%, list=23%, signal=28%
34	KEGG_MAPK_SIGNALING_PATHWAY	267	0.436765149	1.485456888	0.000183064	0.001134996	0.000706562	4107	tags=28%, list=22%, signal=23%
35	KEGG_PATHWAYS_IN_CANCER	325	0.423330822	1.445477408	0.000294217	0.00160954	0.001001977	3859	tags=29%, list=20%, signal=24%
36	KEGG_JAK_STAT_SIGNALING_PATHWAY	155	0.417469773	1.384284172	0.01378763	0.044991214	0.028008113	4824	tags=37%, list=25%, signal=28%
37	KEGG_HUNTINGTONS_DISEASE	172	-0.310861606	-1.519810108	0.000588178	0.002956786	0.001840671	7141	tags=58%, list=37%, signal=36%
38	KEGG_PARKINSONS_DISEASE	113	-0.331419312	-1.593916299	0.001337857	0.006069304	0.003778288	7113	tags=67%, list=37%, signal=42%
39	KEGG_PYRIMIDINE_METABOLISM	97	-0.386347213	-1.767736566	0.000167526	0.001132629	0.000705089	6096	tags=60%, list=32%, signal=41%
40	KEGG_OXIDATIVE_PHOSPHORYLATION	116	-0.389805404	-1.866874972	1.96E-05	0.00018232	0.000113498	7092	tags=69%, list=37%, signal=44%
41	KEGG_PEROXISOME	78	-0.409019987	-1.773083191	0.000176593	0.001132629	0.000705089	8074	tags=74%, list=42%, signal=43%
42	KEGG_PYRUVATE_METABOLISM	40	-0.442585199	-1.672824017	0.007130888	0.025506638	0.015878495	5364	tags=55%, list=28%, signal=40%
43	KEGG_CELL_CYCLE	124	-0.465991873	-2.198897979	1.04E-08	1.76E-07	1.10E-07	2766	tags=40%, list=14%, signal=34%

44	KEGG_SPLICEOSOME	126	-0.479683096	-2.304230335	1.39E-09	3.23E-08	2.01E-08	6838	tags=73%, list=36%, signal=47%
45	KEGG_PROTEASOME	44	-0.484320831	-1.885200254	0.001017094	0.004850754	0.003019711	7278	tags=73%, list=38%, signal=45%
46	KEGG_RNA_DEGRADATION	57	-0.498130823	-2.033020708	3.05E-05	0.000257698	0.000160423	8042	tags=75%, list=42%, signal=44%
47	KEGG_NUCLEOTIDE_EXCISION_REPAIR	44	-0.504048834	-1.961990751	0.000335012	0.001780351	0.001108311	8448	tags=82%, list=44%, signal=46%
48	KEGG_RNA_POLYMERASE	29	-0.528958493	-1.868994026	0.002292495	0.009475648	0.005898819	7226	tags=72%, list=38%, signal=45%
49	KEGG_BASE_EXCISION_REPAIR	33	-0.540398954	-1.9718701	0.000605371	0.002963133	0.001844622	7389	tags=82%, list=39%, signal=50%
50	KEGG_RIBOSOME	87	-0.54539268	-2.416985209	1.68E-09	3.48E-08	2.17E-08	6934	tags=78%, list=36%, signal=50%
51	KEGG_AMINOACYL_TRNA_BIOSYNTHESIS	41	-0.577298693	-2.233019922	2.64E-05	0.000233388	0.000145289	7116	tags=83%, list=37%, signal=52%
52	KEGG_CITRATE_CYCLE_TCA_CYCLE	30	-0.587279863	-2.079298494	0.000172983	0.001132629	0.000705089	4920	tags=63%, list=26%, signal=47%
53	KEGG_HOMOLOGOUS_RECOMBINATION	28	-0.624582127	-2.165788137	0.000219905	0.001239465	0.000771597	2608	tags=46%, list=14%, signal=40%
54	KEGG_MISMATCH_REPAIR	23	-0.648854766	-2.176057671	0.000191935	0.001142037	0.000710946	3418	tags=74%, list=18%, signal=61%

55	KEGG_STEROID_BIOSYNTHESIS	17	-0.699062497	-2.154718348	0.000360246	0.00186127	0.001158685	4095	tags=76%, list=21%, signal=60%
56	KEGG_NITROGEN_METABOLISM	23	-0.734982169	-2.464902271	2.22E-06	2.43E-05	1.51E-05	1607	tags=43%, list=8%, signal=40%
57	KEGG_DNA_REPLICATION	36	-0.768238283	-2.828937728	1.00E-10	3.72E-09	2.32E-09	3466	tags=81%, list=18%, signal=66%

Table S4. The list of GO analysis results between the high-risk and low-risk groups

	Category	Description	p-value	p.adjust	GeneRatio
1	BP	extracellular matrix organization	1.25E-34	5.64E-31	83/1052
2	BP	extracellular structure organization	1.62E-34	5.64E-31	83/1052
3	BP	external encapsulating structure organization	2.72E-34	6.32E-31	83/1052
4	BP	ossification	9.31E-24	1.63E-20	82/1052
5	BP	muscle organ development	5.69E-19	7.94E-16	65/1052
6	BP	muscle tissue development	5.31E-18	6.09E-15	71/1052
7	BP	connective tissue development	6.24E-18	6.09E-15	55/1052
8	BP	cartilage development	6.97E-18	6.09E-15	47/1052
9	BP	muscle system process	1.01E-17	7.81E-15	75/1052
10	BP	muscle contraction	3.77E-16	2.52E-13	62/1052
11	BP	axon development	3.97E-16	2.52E-13	75/1052
12	BP	collagen fibril organization	7.58E-16	4.41E-13	25/1052
13	BP	osteoblast differentiation	2.16E-15	1.16E-12	49/1052
14	BP	muscle cell differentiation	1.56E-14	7.77E-12	63/1052
15	BP	axonogenesis	2.00E-14	9.08E-12	67/1052
16	BP	cell junction assembly	2.08E-14	9.08E-12	66/1052
17	BP	synapse organization	6.13E-14	2.52E-11	65/1052
18	BP	negative regulation of Wnt signaling pathway	2.95E-13	1.14E-10	38/1052
19	BP	cell-substrate adhesion	4.51E-13	1.66E-10	58/1052
20	BP	negative regulation of canonical Wnt signaling pathway	1.74E-12	6.08E-10	33/1052
21	BP	regulation of osteoblast differentiation	2.64E-12	8.78E-10	32/1052
22	BP	transmembrane receptor protein serine/threonine kinase signaling pathway	7.70E-12	2.44E-09	56/1052
23	BP	cell-cell adhesion via plasma-membrane adhesion molecules	1.05E-11	3.19E-09	47/1052
24	BP	striated muscle cell differentiation	1.36E-11	3.83E-09	47/1052

25	BP	mesenchyme development	1.37E-11	3.83E-09	49/1052
26	BP	regulation of metal ion transport	3.27E-11	8.60E-09	58/1052
27	BP	regulation of membrane potential	3.32E-11	8.60E-09	60/1052
28	BP	Wnt signaling pathway	5.29E-11	1.32E-08	62/1052
29	BP	cell-cell signaling by wnt	6.37E-11	1.53E-08	62/1052
30	BP	regulation of Wnt signaling pathway	7.37E-11	1.72E-08	51/1052
31	BP	synapse assembly	8.21E-11	1.85E-08	35/1052
32	BP	heart process	1.00E-10	2.14E-08	43/1052
33	BP	regulation of transmembrane receptor protein serine/threonine kinase signaling pathway	1.01E-10	2.14E-08	44/1052
34	BP	regulation of cell junction assembly	1.25E-10	2.56E-08	37/1052
35	BP	urogenital system development	1.32E-10	2.57E-08	52/1052
36	BP	muscle cell development	1.32E-10	2.57E-08	35/1052
37	BP	regulation of blood circulation	1.47E-10	2.77E-08	43/1052
38	BP	regulation of canonical Wnt signaling pathway	1.66E-10	3.06E-08	43/1052
39	BP	canonical Wnt signaling pathway	1.87E-10	3.35E-08	48/1052
40	BP	regulation of cellular response to growth factor stimulus	2.09E-10	3.58E-08	48/1052
41	BP	sensory system development	2.10E-10	3.58E-08	55/1052
42	BP	neuron projection guidance	2.65E-10	4.41E-08	40/1052
43	BP	heart contraction	3.50E-10	5.69E-08	41/1052
44	BP	striated muscle tissue development	3.98E-10	6.31E-08	41/1052
45	BP	neuron migration	4.81E-10	7.18E-08	32/1052
46	BP	multicellular organismal signaling	4.81E-10	7.18E-08	32/1052
47	BP	bone mineralization	4.83E-10	7.18E-08	27/1052
48	BP	endoderm formation	5.02E-10	7.31E-08	18/1052
49	BP	regulation of heart contraction	5.24E-10	7.46E-08	37/1052
50	BP	second-messenger-mediated signaling	6.28E-10	8.77E-08	46/1052

51	BP	negative regulation of cellular response to growth factor stimulus	7.47E-10	1.02E-07	26/1052
52	BP	learning or memory	8.22E-10	1.07E-07	42/1052
53	BP	axon guidance	8.35E-10	1.07E-07	39/1052
54	BP	cardiac muscle tissue development	8.35E-10	1.07E-07	39/1052
55	BP	ovulation cycle	8.45E-10	1.07E-07	20/1052
56	BP	homophilic cell adhesion via plasma membrane adhesion molecules	9.10E-10	1.13E-07	32/1052
57	BP	visual system development	9.86E-10	1.21E-07	53/1052
58	BP	renal system development	1.65E-09	1.99E-07	46/1052
59	BP	eye development	1.92E-09	2.24E-07	52/1052
60	BP	response to BMP	1.96E-09	2.24E-07	32/1052
61	BP	cellular response to BMP stimulus	1.96E-09	2.24E-07	32/1052
62	BP	regulation of animal organ morphogenesis	2.18E-09	2.46E-07	27/1052
63	BP	vascular process in circulatory system	2.42E-09	2.68E-07	41/1052
64	BP	mesenchymal cell differentiation	2.88E-09	3.14E-07	39/1052
65	BP	regulation of ion transmembrane transport	3.08E-09	3.31E-07	60/1052
66	BP	cell-matrix adhesion	3.29E-09	3.48E-07	38/1052
67	BP	regulation of cation transmembrane transport	3.48E-09	3.63E-07	49/1052
68	BP	morphogenesis of a branching structure	5.24E-09	5.38E-07	34/1052
69	BP	regulation of cartilage development	5.74E-09	5.81E-07	19/1052
70	BP	kidney development	6.01E-09	5.99E-07	44/1052
71	BP	modulation of chemical synaptic transmission	7.76E-09	7.63E-07	55/1052
72	BP	regulation of trans-synaptic signaling	8.43E-09	8.12E-07	55/1052
73	BP	skeletal muscle tissue development	8.50E-09	8.12E-07	29/1052
74	BP	muscle cell proliferation	8.74E-09	8.25E-07	37/1052
75	BP	regulation of synapse assembly	9.12E-09	8.49E-07	23/1052
76	BP	cellular component assembly involved in morphogenesis	1.04E-08	9.52E-07	24/1052

77	BP	skeletal muscle organ development	1.26E-08	1.14E-06	30/1052
78	BP	elastic fiber assembly	1.33E-08	1.17E-06	8/1052
79	BP	myofibril assembly	1.33E-08	1.17E-06	18/1052
80	BP	endodermal cell differentiation	1.49E-08	1.28E-06	15/1052
81	BP	extracellular matrix assembly	1.49E-08	1.28E-06	15/1052
82	BP	formation of primary germ layer	1.60E-08	1.36E-06	25/1052
83	BP	striated muscle cell development	1.72E-08	1.45E-06	18/1052
84	BP	BMP signaling pathway	2.39E-08	1.97E-06	29/1052
85	BP	cognition	2.40E-08	1.97E-06	43/1052
86	BP	potassium ion transport	2.75E-08	2.23E-06	37/1052
87	BP	response to steroid hormone	2.86E-08	2.29E-06	45/1052
88	BP	negative regulation of cartilage development	3.00E-08	2.38E-06	12/1052
89	BP	adenylate cyclase-modulating G protein-coupled receptor signaling pathway	3.07E-08	2.40E-06	37/1052
90	BP	collagen metabolic process	3.09E-08	2.40E-06	22/1052
91	BP	chondrocyte differentiation	3.41E-08	2.62E-06	23/1052
92	BP	bone development	3.75E-08	2.85E-06	35/1052
93	BP	ovulation cycle process	3.91E-08	2.91E-06	15/1052
94	BP	morphogenesis of a branching epithelium	3.93E-08	2.91E-06	31/1052
95	BP	endoderm development	3.96E-08	2.91E-06	19/1052
96	BP	smooth muscle contraction	4.86E-08	3.52E-06	23/1052
97	BP	regulation of cell-substrate adhesion	4.89E-08	3.52E-06	34/1052
98	BP	glial cell differentiation	5.49E-08	3.91E-06	34/1052
99	BP	regulation of muscle system process	5.86E-08	4.13E-06	37/1052
100	BP	epithelial cell proliferation	5.98E-08	4.13E-06	54/1052
101	BP	negative regulation of cell motility	5.98E-08	4.13E-06	47/1052
102	BP	regulation of synapse organization	6.35E-08	4.35E-06	33/1052

103	BP	actomyosin structure organization	6.48E-08	4.36E-06	32/1052
104	BP	regulation of BMP signaling pathway	6.49E-08	4.36E-06	22/1052
105	BP	regulation of morphogenesis of an epithelium	6.62E-08	4.40E-06	17/1052
106	BP	gland morphogenesis	7.02E-08	4.63E-06	24/1052
107	BP	negative regulation of osteoblast differentiation	7.12E-08	4.63E-06	15/1052
108	BP	biomineral tissue development	7.17E-08	4.63E-06	29/1052
109	BP	camera-type eye development	7.90E-08	5.06E-06	44/1052
110	BP	neuron cell-cell adhesion	8.41E-08	5.34E-06	9/1052
111	BP	cardiac conduction	8.55E-08	5.38E-06	21/1052
112	BP	calcium ion transport	8.63E-08	5.38E-06	52/1052
113	BP	heart morphogenesis	8.90E-08	5.50E-06	37/1052
114	BP	biomineralization	9.32E-08	5.71E-06	29/1052
115	BP	pattern specification process	1.08E-07	6.54E-06	55/1052
116	BP	negative regulation of transmembrane receptor protein serine/threonine kinase signaling pathway	1.10E-07	6.63E-06	25/1052
117	BP	negative regulation of cellular component movement	1.17E-07	6.96E-06	47/1052
118	BP	negative regulation of cell migration	1.18E-07	6.96E-06	45/1052
119	BP	wound healing	1.26E-07	7.36E-06	52/1052
120	BP	regulation of synapse structure or activity	1.27E-07	7.36E-06	33/1052
121	BP	gliogenesis	1.35E-07	7.77E-06	40/1052
122	BP	proximal/distal pattern formation	1.54E-07	8.82E-06	12/1052
123	BP	regulation of vasculature development	1.79E-07	1.02E-05	45/1052
124	BP	potassium ion transmembrane transport	1.97E-07	1.11E-05	33/1052
125	BP	positive regulation of ion transport	1.99E-07	1.11E-05	38/1052
126	BP	postsynapse assembly	2.18E-07	1.21E-05	11/1052
127	BP	gastrulation	2.50E-07	1.37E-05	30/1052

128	BP	regulation of cytosolic calcium ion concentration	2.70E-07	1.47E-05	45/1052
129	BP	regulation of nervous system development	2.82E-07	1.52E-05	52/1052
130	BP	regulation of angiogenesis	2.84E-07	1.53E-05	44/1052
131	BP	cell fate commitment	2.92E-07	1.55E-05	37/1052
132	BP	sensory organ morphogenesis	2.92E-07	1.55E-05	37/1052
133	BP	female sex differentiation	3.42E-07	1.79E-05	23/1052
134	BP	action potential	3.48E-07	1.81E-05	25/1052
135	BP	regulation of neuron projection development	3.53E-07	1.82E-05	51/1052
136	BP	postsynapse organization	3.70E-07	1.89E-05	27/1052
137	BP	smooth muscle cell proliferation	3.71E-07	1.89E-05	28/1052
138	BP	leukocyte migration	3.82E-07	1.93E-05	47/1052
139	BP	negative regulation of locomotion	3.84E-07	1.93E-05	48/1052
140	BP	fat cell differentiation	4.24E-07	2.11E-05	34/1052
141	BP	negative regulation of BMP signaling pathway	4.59E-07	2.27E-05	15/1052
142	BP	embryonic organ morphogenesis	4.89E-07	2.40E-05	39/1052
143	BP	glomerulus development	4.99E-07	2.40E-05	16/1052
144	BP	regulation of chondrocyte differentiation	4.99E-07	2.40E-05	14/1052
145	BP	regulation of morphogenesis of a branching structure	4.99E-07	2.40E-05	14/1052
146	BP	skeletal system morphogenesis	5.09E-07	2.43E-05	33/1052
147	BP	regulation of leukocyte migration	5.48E-07	2.60E-05	32/1052
148	BP	regulation of potassium ion transport	6.68E-07	3.15E-05	21/1052
149	BP	prostate gland epithelium morphogenesis	6.73E-07	3.15E-05	10/1052
150	BP	lung development	7.57E-07	3.52E-05	28/1052
151	BP	calcium ion homeostasis	8.27E-07	3.82E-05	53/1052
152	BP	cellular calcium ion homeostasis	8.50E-07	3.91E-05	52/1052
153	BP	kidney epithelium development	8.60E-07	3.93E-05	24/1052

154	BP	regulation of smooth muscle cell proliferation	8.74E-07	3.96E-05	27/1052
155	BP	reproductive structure development	9.54E-07	4.30E-05	50/1052
156	BP	regulation of tube diameter	9.83E-07	4.37E-05	24/1052
157	BP	blood vessel diameter maintenance	9.83E-07	4.37E-05	24/1052
158	BP	regulation of neuron differentiation	1.02E-06	4.49E-05	29/1052
159	BP	respiratory system development	1.06E-06	4.67E-05	30/1052
160	BP	regulation of chemotaxis	1.12E-06	4.80E-05	32/1052
161	BP	appendage morphogenesis	1.12E-06	4.80E-05	24/1052
162	BP	limb morphogenesis	1.12E-06	4.80E-05	24/1052
163	BP	regulation of tube size	1.12E-06	4.80E-05	24/1052
164	BP	reproductive system development	1.17E-06	4.98E-05	50/1052
165	BP	positive regulation of nervous system development	1.18E-06	5.00E-05	36/1052
166	BP	respiratory tube development	1.19E-06	5.02E-05	28/1052
167	BP	embryonic organ development	1.22E-06	5.09E-05	51/1052
168	BP	forebrain development	1.25E-06	5.21E-05	45/1052
169	BP	regulation of cell development	1.29E-06	5.32E-05	55/1052
170	BP	tissue remodeling	1.39E-06	5.72E-05	27/1052
171	BP	regionalization	1.45E-06	5.94E-05	43/1052
172	BP	artery development	1.49E-06	6.03E-05	20/1052
173	BP	prostate gland morphogenesis	1.50E-06	6.05E-05	10/1052
174	BP	learning	1.65E-06	6.55E-05	24/1052
175	BP	nephron development	1.65E-06	6.55E-05	24/1052
176	BP	myeloid leukocyte migration	1.65E-06	6.55E-05	32/1052
177	BP	metanephros development	1.73E-06	6.84E-05	18/1052
178	BP	cellular response to transforming growth factor beta stimulus	1.78E-06	6.96E-05	34/1052
179	BP	ear development	1.81E-06	7.07E-05	31/1052

180	BP	cellular divalent inorganic cation homeostasis	1.94E-06	7.49E-05	54/1052
181	BP	prostate gland development	1.95E-06	7.49E-05	13/1052
182	BP	digestive tract morphogenesis	1.95E-06	7.49E-05	13/1052
183	BP	embryonic skeletal system development	2.03E-06	7.76E-05	22/1052
184	BP	collagen catabolic process	2.05E-06	7.78E-05	12/1052
185	BP	regulation of ossification	2.21E-06	8.32E-05	21/1052
186	BP	development of primary female sexual characteristics	2.34E-06	8.79E-05	20/1052
187	BP	positive regulation of cytosolic calcium ion concentration	2.42E-06	9.03E-05	40/1052
188	BP	presynapse assembly	2.50E-06	9.27E-05	13/1052
189	BP	embryonic limb morphogenesis	2.54E-06	9.27E-05	21/1052
190	BP	embryonic appendage morphogenesis	2.54E-06	9.27E-05	21/1052
191	BP	cytosolic calcium ion transport	2.55E-06	9.27E-05	28/1052
192	BP	response to ketone	2.55E-06	9.27E-05	28/1052
193	BP	positive regulation of osteoblast differentiation	2.57E-06	9.30E-05	16/1052
194	BP	response to vitamin	2.78E-06	0.000100207	17/1052
195	BP	response to alcohol	2.91E-06	0.000104051	32/1052
196	BP	response to transforming growth factor beta	3.03E-06	0.000107787	34/1052
197	BP	epithelial tube morphogenesis	3.04E-06	0.000107814	40/1052
198	BP	positive regulation of chemotaxis	3.08E-06	0.000108596	23/1052
199	BP	calcium ion transport into cytosol	3.20E-06	0.000112312	26/1052
200	BP	regulation of epithelial to mesenchymal transition	3.30E-06	0.000115142	19/1052
201	BP	positive regulation of leukocyte migration	3.49E-06	0.000121173	23/1052
202	BP	epithelial to mesenchymal transition	3.75E-06	0.000129588	25/1052
203	BP	memory	3.83E-06	0.000131814	21/1052
204	BP	presynapse organization	4.04E-06	0.000138087	13/1052
205	BP	regulation of transmembrane transporter activity	4.08E-06	0.000139066	35/1052

206	BP	cellular response to vitamin	4.35E-06	0.000147065	10/1052
207	BP	cardiocyte differentiation	4.36E-06	0.000147065	24/1052
208	BP	cell adhesion mediated by integrin	4.64E-06	0.000155121	17/1052
209	BP	regulation of ion transmembrane transporter activity	4.64E-06	0.000155121	34/1052
210	BP	synaptic membrane adhesion	4.70E-06	0.000156279	9/1052
211	BP	organ growth	4.95E-06	0.000163392	26/1052
212	BP	cell growth	4.96E-06	0.000163392	52/1052
213	BP	regulation of leukocyte chemotaxis	5.00E-06	0.00016394	21/1052
214	BP	female gonad development	5.15E-06	0.000168086	19/1052
215	BP	ovulation from ovarian follicle	5.23E-06	0.000169905	6/1052
216	BP	response to oxygen levels	5.52E-06	0.000178417	39/1052
217	BP	monocyte chemotaxis	5.92E-06	0.000190435	15/1052
218	BP	appendage development	6.11E-06	0.000194798	26/1052
219	BP	limb development	6.11E-06	0.000194798	26/1052
220	BP	extracellular matrix disassembly	6.31E-06	0.000200224	14/1052
221	BP	calcium ion transmembrane transport	6.40E-06	0.000202039	38/1052
222	BP	postsynaptic specialization assembly	6.44E-06	0.00020265	8/1052
223	BP	cardiac chamber development	6.52E-06	0.000204232	25/1052
224	BP	response to corticosteroid	6.88E-06	0.000213809	24/1052
225	BP	cell chemotaxis	6.89E-06	0.000213809	38/1052
226	BP	positive regulation of MAPK cascade	7.47E-06	0.000230862	52/1052
227	BP	regulation of transporter activity	7.98E-06	0.000245393	37/1052
228	BP	central nervous system neuron differentiation	8.08E-06	0.000247451	25/1052
229	BP	postsynaptic specialization organization	8.19E-06	0.000249591	10/1052
230	BP	positive regulation of transmembrane transport	8.39E-06	0.000254564	29/1052
231	BP	mesonephros development	8.46E-06	0.00025489	18/1052

232	BP	calcium-mediated signaling	8.47E-06	0.00025489	28/1052
233	BP	non-canonical Wnt signaling pathway	8.53E-06	0.000255715	15/1052
234	BP	leukocyte chemotaxis	8.72E-06	0.000260036	31/1052
235	BP	regulation of muscle contraction	8.98E-06	0.000266803	25/1052
236	BP	regulation of peptidase activity	9.22E-06	0.000272662	49/1052
237	BP	regulation of hormone levels	9.94E-06	0.000292849	52/1052
238	BP	muscle tissue morphogenesis	1.02E-05	0.000298971	15/1052
239	BP	positive regulation of neuron differentiation	1.03E-05	0.000299536	17/1052
240	BP	regulation of epithelial cell proliferation	1.03E-05	0.000299536	43/1052
241	BP	intramembranous ossification	1.04E-05	0.000299536	5/1052
242	BP	direct ossification	1.04E-05	0.000299536	5/1052
243	BP	digestive tract development	1.07E-05	0.000306992	21/1052
244	BP	rhythmic process	1.07E-05	0.000306992	36/1052
245	BP	regulation of presynapse organization	1.10E-05	0.000312724	10/1052
246	BP	regulation of presynapse assembly	1.10E-05	0.000312724	10/1052
247	BP	multi-organism reproductive process	1.12E-05	0.000317162	28/1052
248	BP	regulation of blood pressure	1.13E-05	0.000317162	26/1052
249	BP	regulation of calcium ion transport	1.17E-05	0.000328347	32/1052
250	BP	neuron recognition	1.19E-05	0.000332162	12/1052
251	BP	regulation of postsynaptic membrane potential	1.21E-05	0.000336086	21/1052
252	BP	cyclic-nucleotide-mediated signaling	1.23E-05	0.000340769	16/1052
253	BP	regulation of fat cell differentiation	1.32E-05	0.00036322	22/1052
254	BP	digestive system development	1.32E-05	0.00036322	22/1052
255	BP	sarcoplasmic reticulum calcium ion transport	1.38E-05	0.000375623	11/1052
256	BP	positive regulation of cell-substrate adhesion	1.38E-05	0.000375623	20/1052
257	BP	branching morphogenesis of an epithelial tube	1.41E-05	0.00038227	23/1052

258	BP	release of sequestered calcium ion into cytosol by sarcoplasmic reticulum	1.47E-05	0.000396624	10/1052
259	BP	calcium ion transmembrane import into cytosol	1.57E-05	0.000422743	23/1052
260	BP	protein localization to synapse	1.63E-05	0.00043895	14/1052
261	BP	regulation of cell growth	1.69E-05	0.00045136	45/1052
262	BP	muscle cell migration	1.71E-05	0.000456913	18/1052
263	BP	sarcomere organization	1.76E-05	0.000466029	11/1052
264	BP	regulation of potassium ion transmembrane transport	1.86E-05	0.00049166	17/1052
265	BP	release of sequestered calcium ion into cytosol by endoplasmic reticulum	1.93E-05	0.000508006	10/1052
266	BP	regulation of heart rate	1.96E-05	0.000513246	18/1052
267	BP	positive regulation of cell junction assembly	1.96E-05	0.000513246	18/1052
268	BP	ovulation	2.16E-05	0.000559928	8/1052
269	BP	negative regulation of chondrocyte differentiation	2.16E-05	0.000559928	8/1052
270	BP	adult behavior	2.17E-05	0.000561586	21/1052
271	BP	cardiac chamber morphogenesis	2.24E-05	0.000575989	20/1052
272	BP	cardiac ventricle development	2.24E-05	0.000575989	20/1052
273	BP	social behavior	2.28E-05	0.000580342	12/1052
274	BP	membrane repolarization	2.28E-05	0.000580342	12/1052
275	BP	multi-multicellular organism process	2.30E-05	0.000584882	28/1052
276	BP	regulation of cation channel activity	2.45E-05	0.000618925	25/1052
277	BP	urinary bladder smooth muscle contraction	2.64E-05	0.000662532	5/1052
278	BP	amacrine cell differentiation	2.64E-05	0.000662532	5/1052
279	BP	locomotory behavior	2.66E-05	0.000662532	26/1052
280	BP	inner ear development	2.66E-05	0.000662532	26/1052
281	BP	positive regulation of epithelial to mesenchymal transition	2.80E-05	0.0006904	12/1052
282	BP	Wnt signaling pathway, planar cell polarity pathway	2.80E-05	0.0006904	12/1052
283	BP	negative regulation of peptidase activity	2.80E-05	0.0006904	32/1052

284	BP	regulation of ERK1 and ERK2 cascade	2.94E-05	0.000722008	36/1052
285	BP	response to glucocorticoid	3.04E-05	0.000744307	21/1052
286	BP	muscle organ morphogenesis	3.22E-05	0.000786408	15/1052
287	BP	forelimb morphogenesis	3.24E-05	0.000787594	10/1052
288	BP	biological process involved in intraspecies interaction between organisms	3.41E-05	0.000826496	12/1052
289	BP	regulation of release of sequestered calcium ion into cytosol by sarcoplasmic reticulum	3.42E-05	0.000826496	9/1052
290	BP	odontogenesis	3.57E-05	0.000859644	20/1052
291	BP	positive regulation of synapse assembly	3.75E-05	0.000899968	13/1052
292	BP	smooth muscle cell migration	4.07E-05	0.000972661	16/1052
293	BP	release of sequestered calcium ion into cytosol	4.16E-05	0.000991499	19/1052
294	BP	regulation of muscle cell differentiation	4.32E-05	0.001026732	22/1052
295	BP	negative regulation of smooth muscle cell proliferation	4.52E-05	0.001067283	14/1052
296	BP	basement membrane organization	4.53E-05	0.001067283	9/1052
297	BP	response to cortisol	4.66E-05	0.001082067	4/1052
298	BP	mesenchyme migration	4.66E-05	0.001082067	4/1052
299	BP	cell-substrate junction assembly	4.67E-05	0.001082067	16/1052
300	BP	negative regulation of sequestering of calcium ion	4.67E-05	0.001082067	19/1052
301	BP	response to hyperoxia	4.68E-05	0.001082067	7/1052
302	BP	vocalization behavior	4.68E-05	0.001082067	7/1052
303	BP	eye morphogenesis	4.78E-05	0.001102049	22/1052
304	BP	receptor clustering	5.01E-05	0.001150696	12/1052
305	BP	regulation of smooth muscle cell migration	5.05E-05	0.001155382	15/1052
306	BP	regeneration	5.12E-05	0.001167176	25/1052
307	BP	positive regulation of epithelial cell proliferation	5.16E-05	0.001173269	27/1052
308	BP	positive regulation of Wnt signaling pathway	5.19E-05	0.001175504	21/1052
309	BP	response to estradiol	5.24E-05	0.001184757	19/1052

310	BP	cellular response to fibroblast growth factor stimulus	5.42E-05	0.001219498	18/1052
311	BP	regulation of calcium ion transport into cytosol	5.47E-05	0.001222821	17/1052
312	BP	actin-mediated cell contraction	5.47E-05	0.001222821	17/1052
313	BP	prostatic bud formation	5.67E-05	0.001263942	5/1052
314	BP	striated muscle contraction	5.74E-05	0.001275775	24/1052
315	BP	regulation of sequestering of calcium ion	5.87E-05	0.001301767	19/1052
316	BP	embryonic digestive tract development	5.92E-05	0.001306805	9/1052
317	BP	regulation of establishment of planar polarity	6.03E-05	0.001327373	12/1052
318	BP	ureteric bud development	6.12E-05	0.001340066	16/1052
319	BP	oligodendrocyte differentiation	6.12E-05	0.001340066	16/1052
320	BP	cardiac muscle cell contraction	6.17E-05	0.001345669	14/1052
321	BP	endocrine process	6.72E-05	0.001461544	15/1052
322	BP	type B pancreatic cell proliferation	6.85E-05	0.001486089	7/1052
323	BP	mesonephric epithelium development	6.99E-05	0.001505466	16/1052
324	BP	mesonephric tubule development	6.99E-05	0.001505466	16/1052
325	BP	ameboidal-type cell migration	7.13E-05	0.001532001	48/1052
326	BP	negative regulation of cell development	7.51E-05	0.001608624	24/1052
327	BP	positive regulation of cation transmembrane transport	7.79E-05	0.001662604	21/1052
328	BP	regulation of extracellular matrix organization	7.96E-05	0.001683031	11/1052
329	BP	regulation of striated muscle cell differentiation	7.96E-05	0.001683031	16/1052
330	BP	protein localization to cell junction	7.96E-05	0.001683031	16/1052
331	BP	nitric oxide mediated signal transduction	8.00E-05	0.001686299	8/1052
332	BP	cellular response to nutrient	8.22E-05	0.001727918	10/1052
333	BP	cardiac muscle contraction	8.48E-05	0.001776735	20/1052
334	BP	gland development	8.78E-05	0.001836102	44/1052
335	BP	response to chemokine	9.04E-05	0.001878315	16/1052

336	BP	cellular response to chemokine	9.04E-05	0.001878315	16/1052
337	BP	sequestering of calcium ion	9.12E-05	0.001888465	19/1052
338	BP	response to molecule of bacterial origin	9.26E-05	0.001911933	38/1052
339	BP	response to nutrient	9.48E-05	0.001952191	21/1052
340	BP	positive regulation of cell adhesion	9.66E-05	0.001982765	45/1052
341	BP	morphogenesis of an epithelial bud	9.78E-05	0.001992169	6/1052
342	BP	cell migration involved in heart development	9.79E-05	0.001992169	7/1052
343	BP	long-term memory	9.79E-05	0.001992169	9/1052
344	BP	neuropeptide signaling pathway	0.000100689	0.002043344	17/1052
345	BP	stem cell development	0.000101355	0.002050901	15/1052
346	BP	regulation of neuron migration	0.000101757	0.002053084	10/1052
347	BP	cardiac muscle tissue morphogenesis	0.000102139	0.002054843	12/1052
348	BP	cell-substrate junction organization	0.000102523	0.002056656	16/1052
349	BP	positive regulation of ERK1 and ERK2 cascade	0.000106052	0.002117725	27/1052
350	BP	regulation of osteoblast proliferation	0.000106478	0.002117725	8/1052
351	BP	regulation of monocyte chemotaxis	0.000106478	0.002117725	8/1052
352	BP	response to fibroblast growth factor	0.000108045	0.002137955	18/1052
353	BP	urinary tract smooth muscle contraction	0.000108107	0.002137955	5/1052
354	BP	sex differentiation	0.000108847	0.002146491	32/1052
355	BP	regulation of synaptic transmission, glutamatergic	0.000118275	0.002325849	13/1052
356	BP	response to lipopolysaccharide	0.000121551	0.002383557	36/1052
357	BP	response to peptide	0.000123522	0.002415423	48/1052
358	BP	cGMP-mediated signaling	0.000124081	0.002419585	9/1052
359	BP	regulation of release of sequestered calcium ion into cytosol	0.000127875	0.002486623	14/1052
360	BP	developmental maturation	0.000128659	0.00249491	33/1052
361	BP	apoptotic process involved in heart morphogenesis	0.000133532	0.002560957	4/1052

362	BP	regulation of synaptic transmission, cholinergic	0.000133532	0.002560957	4/1052
363	BP	aorta smooth muscle tissue morphogenesis	0.000133532	0.002560957	4/1052
364	BP	smooth muscle cell-matrix adhesion	0.000133532	0.002560957	4/1052
365	BP	cardiac muscle cell differentiation	0.000134386	0.00257027	18/1052
366	BP	ERK1 and ERK2 cascade	0.000137062	0.002614288	36/1052
367	BP	mesenchyme morphogenesis	0.000139176	0.002647377	11/1052
368	BP	aorta development	0.000142213	0.002684986	12/1052
369	BP	regulation of pathway-restricted SMAD protein phosphorylation	0.000142213	0.002684986	12/1052
370	BP	nephron epithelium development	0.000142307	0.002684986	17/1052
371	BP	regulation of bone mineralization	0.000146755	0.002754026	14/1052
372	BP	cardiac muscle cell development	0.000146755	0.002754026	14/1052
373	BP	metanephric glomerulus development	0.000149118	0.002783411	6/1052
374	BP	regulation of Wnt signaling pathway, planar cell polarity pathway	0.000149118	0.002783411	6/1052
375	BP	myotube differentiation	0.000149554	0.002784093	18/1052
376	BP	response to hypoxia	0.000151299	0.002809083	32/1052
377	BP	body morphogenesis	0.000152916	0.002830364	10/1052
378	BP	regulation of wound healing	0.000153256	0.002830364	19/1052
379	BP	response to decreased oxygen levels	0.000155728	0.002863933	33/1052
380	BP	dopaminergic neuron differentiation	0.000155894	0.002863933	9/1052
381	BP	cardiac ventricle morphogenesis	0.00015929	0.002911005	13/1052
382	BP	negative regulation of neuron differentiation	0.00015929	0.002911005	13/1052
383	BP	cardiac muscle cell action potential involved in contraction	0.000166047	0.003021495	11/1052
384	BP	ear morphogenesis	0.000166202	0.003021495	18/1052
385	BP	neuron fate commitment	0.000166848	0.003025367	12/1052
386	BP	spinal cord interneuron axon guidance	0.000174744	0.003146436	3/1052
387	BP	regulation of amacrine cell differentiation	0.000174744	0.003146436	3/1052

388	BP	negative regulation of endopeptidase activity	0.000174877	0.003146436	29/1052
389	BP	hemostasis	0.000179281	0.003217384	27/1052
390	BP	motor neuron axon guidance	0.00018145	0.003247952	8/1052
391	BP	multicellular organismal response to stress	0.00018402	0.003285539	13/1052
392	BP	morphogenesis of an epithelial fold	0.000186976	0.0033298	7/1052
393	BP	lateral sprouting from an epithelium	0.000189059	0.003349807	5/1052
394	BP	postsynaptic membrane assembly	0.000189059	0.003349807	5/1052
395	BP	focal adhesion assembly	0.000191842	0.00338708	14/1052
396	BP	kidney morphogenesis	0.000192162	0.00338708	15/1052
397	BP	regulation of actin filament-based process	0.000192619	0.00338708	40/1052
398	BP	regulation of systemic arterial blood pressure by hormone	0.000194226	0.003406761	9/1052
399	BP	regulation of anatomical structure size	0.000195267	0.003416444	47/1052
400	BP	regulation of heart growth	0.000211974	0.003699469	13/1052
401	BP	positive regulation of leukocyte chemotaxis	0.000217022	0.003768731	15/1052
402	BP	embryonic skeletal system morphogenesis	0.000217022	0.003768731	15/1052
403	BP	embryonic digestive tract morphogenesis	0.000219576	0.003803631	6/1052
404	BP	hormone metabolic process	0.000222532	0.003845284	27/1052
405	BP	regulation of smooth muscle contraction	0.000227188	0.003906407	12/1052
406	BP	animal organ formation	0.000227188	0.003906407	12/1052
407	BP	postsynaptic density organization	0.000232662	0.003990694	8/1052
408	BP	regulation of membrane repolarization	0.000240081	0.004097808	9/1052
409	BP	regulation of heart rate by cardiac conduction	0.000240081	0.004097808	9/1052
410	BP	neural crest cell differentiation	0.000244613	0.004164978	15/1052
411	BP	central nervous system myelination	0.000251292	0.004247628	7/1052
412	BP	axon ensheathment in central nervous system	0.000251292	0.004247628	7/1052
413	BP	regulation of insulin-like growth factor receptor signaling pathway	0.000251292	0.004247628	7/1052

414	BP	pathway-restricted SMAD protein phosphorylation	0.00026374	0.004447265	12/1052
415	BP	regulation of systemic arterial blood pressure mediated by a chemical signal	0.000269158	0.004527695	10/1052
416	BP	heart growth	0.000275178	0.004617833	15/1052
417	BP	female pregnancy	0.000276714	0.004632477	23/1052
418	BP	regulation of neurogenesis	0.000287747	0.004805645	37/1052
419	BP	developmental growth involved in morphogenesis	0.000294501	0.004890484	27/1052
420	BP	metanephric nephron development	0.000294562	0.004890484	9/1052
421	BP	aortic valve morphogenesis	0.000295131	0.004890484	8/1052
422	BP	hindgut morphogenesis	0.00029773	0.004890484	4/1052
423	BP	lung growth	0.00029773	0.004890484	4/1052
424	BP	response to heparin	0.00029773	0.004890484	4/1052
425	BP	negative regulation of monocyte chemotaxis	0.00029773	0.004890484	4/1052
426	BP	regulation of cardiac muscle tissue growth	0.000305167	0.004989156	12/1052
427	BP	regulation of potassium ion transmembrane transporter activity	0.000305167	0.004989156	12/1052
428	BP	positive regulation of ion transmembrane transport	0.000308201	0.005007873	21/1052
429	BP	regulation of chemokine production	0.000308978	0.005007873	15/1052
430	BP	luteinization	0.000309181	0.005007873	5/1052
431	BP	negative regulation of cell adhesion mediated by integrin	0.000309181	0.005007873	5/1052
432	BP	negative regulation of vascular permeability	0.000313833	0.005059749	6/1052
433	BP	negative regulation of bone remodeling	0.000313833	0.005059749	6/1052
434	BP	nephron morphogenesis	0.000318661	0.005091051	13/1052
435	BP	cardiac muscle cell action potential	0.000318661	0.005091051	13/1052
436	BP	cardiac cell development	0.000318692	0.005091051	14/1052
437	BP	cardiac muscle tissue growth	0.000318692	0.005091051	14/1052
438	BP	metal ion export	0.000321425	0.005106231	10/1052
439	BP	cell differentiation involved in kidney development	0.000321837	0.005106231	11/1052

440	BP	cell communication involved in cardiac conduction	0.000321837	0.005106231	11/1052
441	BP	mononuclear cell migration	0.000325878	0.005158627	24/1052
442	BP	excitatory synapse assembly	0.000332274	0.00524797	7/1052
443	BP	response to peptide hormone	0.000340416	0.005364427	40/1052
444	BP	chemokine production	0.000346289	0.005444697	15/1052
445	BP	mesenchymal cell proliferation	0.000358877	0.005604746	9/1052
446	BP	pituitary gland development	0.000358877	0.005604746	9/1052
447	BP	apoptotic process involved in development	0.000358877	0.005604746	9/1052
448	BP	mesenchymal cell development	0.00035981	0.005606769	14/1052
449	BP	response to retinoic acid	0.000362748	0.005620828	16/1052
450	BP	positive regulation of canonical Wnt signaling pathway	0.000362748	0.005620828	16/1052
451	BP	peripheral nervous system development	0.000363128	0.005620828	13/1052
452	BP	positive regulation of sodium ion transport	0.000370644	0.005711847	8/1052
453	BP	osteoblast proliferation	0.000370644	0.005711847	8/1052
454	BP	tissue migration	0.000375833	0.005779054	37/1052
455	BP	positive regulation of cyclase activity	0.000381893	0.005859326	10/1052
456	BP	sensory perception of pain	0.000387405	0.005930869	15/1052
457	BP	regulation of response to wounding	0.000395605	0.006031719	21/1052
458	BP	coagulation	0.000395721	0.006031719	26/1052
459	BP	protein localization to cell surface	0.000404726	0.006152059	12/1052
460	BP	chemokine-mediated signaling pathway	0.000405378	0.006152059	14/1052
461	BP	positive regulation of phosphatidylinositol 3-kinase signaling	0.000412762	0.006237001	13/1052
462	BP	artery morphogenesis	0.000412762	0.006237001	13/1052
463	BP	developmental cell growth	0.000423718	0.006388716	26/1052
464	BP	regulation of developmental growth	0.000428899	0.006452904	34/1052
465	BP	response to increased oxygen levels	0.000432898	0.006485106	7/1052

466	BP	regulation of non-canonical Wnt signaling pathway	0.000432898	0.006485106	7/1052
467	BP	heterophilic cell-cell adhesion via plasma membrane cell adhesion molecules	0.000451537	0.006706758	10/1052
468	BP	retinol metabolic process	0.000451537	0.006706758	10/1052
469	BP	positive regulation of potassium ion transport	0.000451537	0.006706758	10/1052
470	BP	positive regulation of lyase activity	0.000451537	0.006706758	10/1052
471	BP	regulation of sodium ion transport	0.000455781	0.006755435	14/1052
472	BP	outflow tract morphogenesis	0.000468039	0.006922416	13/1052
473	BP	maintenance of location	0.000478676	0.007059389	34/1052
474	BP	regulation of respiratory gaseous exchange by nervous system process	0.000479323	0.007059389	5/1052
475	BP	regulation of biomineral tissue development	0.000482317	0.007088538	15/1052
476	BP	positive regulation of developmental growth	0.000503898	0.007390157	21/1052
477	BP	calcium ion import	0.000511425	0.007469166	14/1052
478	BP	nephron tubule development	0.000511425	0.007469166	14/1052
479	BP	calcium-dependent cell-cell adhesion via plasma membrane cell adhesion molecules	0.000522378	0.007613197	9/1052
480	BP	nerve development	0.000529461	0.007698003	13/1052
481	BP	positive regulation of cation channel activity	0.000530402	0.007698003	12/1052
482	BP	peptidyl-tyrosine phosphorylation	0.000539361	0.007811777	37/1052
483	BP	acute inflammatory response	0.000546959	0.00790543	16/1052
484	BP	endothelial cell proliferation	0.000548298	0.0079084	23/1052
485	BP	development of primary sexual characteristics	0.000553888	0.007959881	26/1052
486	BP	glomerulus vasculature development	0.000556428	0.007959881	7/1052
487	BP	positive regulation of potassium ion transmembrane transporter activity	0.000556428	0.007959881	7/1052
488	BP	positive regulation of extracellular matrix organization	0.000556428	0.007959881	7/1052
489	BP	wound healing, spreading of cells	0.000568792	0.008025374	8/1052
490	BP	epiboly involved in wound healing	0.000568792	0.008025374	8/1052
491	BP	hepatic stellate cell activation	0.000569053	0.008025374	4/1052

492	BP	hindgut development	0.000569053	0.008025374	4/1052
493	BP	motor learning	0.000569053	0.008025374	4/1052
494	BP	postsynaptic density protein 95 clustering	0.000569053	0.008025374	4/1052
495	BP	regulation of collagen fibril organization	0.000569053	0.008025374	4/1052
496	BP	dorsal/ventral pattern formation	0.000572742	0.008044891	14/1052
497	BP	regulation of amine transport	0.000572742	0.008044891	14/1052
498	BP	positive regulation of angiogenesis	0.000592604	0.008290512	22/1052
499	BP	positive regulation of vasculature development	0.000592604	0.008290512	22/1052
500	BP	atrial cardiac muscle tissue development	0.000595016	0.008294209	6/1052
501	BP	segmentation	0.000596432	0.008294209	15/1052
502	BP	regulation of biomineralization	0.000596432	0.008294209	15/1052
503	BP	establishment of planar polarity	0.000604625	0.008374774	12/1052
504	BP	establishment of tissue polarity	0.000604625	0.008374774	12/1052
505	BP	lung alveolus development	0.000624523	0.008615174	9/1052
506	BP	positive regulation of potassium ion transmembrane transport	0.000624523	0.008615174	9/1052
507	BP	peptidyl-tyrosine modification	0.000626917	0.008615174	37/1052
508	BP	regulation of supramolecular fiber organization	0.000626917	0.008615174	37/1052
509	BP	positive regulation of cell development	0.000637554	0.008744139	31/1052
510	BP	blood coagulation	0.000651107	0.008912502	25/1052
511	BP	phospholipase C-activating G protein-coupled receptor signaling pathway	0.000661625	0.009008344	15/1052
512	BP	glial cell development	0.000665815	0.009008344	16/1052
513	BP	positive regulation of synaptic transmission, cholinergic	0.000669722	0.009008344	3/1052
514	BP	endothelial cell-cell adhesion	0.000669722	0.009008344	3/1052
515	BP	regulation of establishment of blood-brain barrier	0.000669722	0.009008344	3/1052
516	BP	Wnt signaling pathway involved in somitogenesis	0.000669722	0.009008344	3/1052
517	BP	interneuron axon guidance	0.000669722	0.009008344	3/1052

518	BP	regulation of smooth muscle cell-matrix adhesion	0.000669722	0.009008344	3/1052
519	BP	positive regulation of hepatic stellate cell activation	0.000669722	0.009008344	3/1052
520	BP	neural crest cell development	0.000672911	0.009016889	13/1052
521	BP	regulation of focal adhesion assembly	0.000674232	0.009016889	11/1052
522	BP	regulation of cell-substrate junction assembly	0.000674232	0.009016889	11/1052
523	BP	transmission of nerve impulse	0.000687367	0.009174966	12/1052
524	BP	regulation of nervous system process	0.000695418	0.009217736	18/1052
525	BP	postsynaptic membrane organization	0.000695853	0.009217736	8/1052
526	BP	membrane depolarization during action potential	0.000695853	0.009217736	8/1052
527	BP	epiboly	0.000695853	0.009217736	8/1052
528	BP	response to isoquinoline alkaloid	0.000706411	0.009304633	7/1052
529	BP	response to morphine	0.000706411	0.009304633	7/1052
530	BP	regulation of cardiac conduction	0.000706411	0.009304633	7/1052
531	BP	synaptic transmission, glutamatergic	0.00071424	0.009372388	14/1052
532	BP	renal tubule development	0.00071424	0.009372388	14/1052
533	BP	regulation of mononuclear cell migration	0.000733079	0.009601541	16/1052
534	BP	somite development	0.000756105	0.00988458	13/1052
535	BP	granulocyte chemotaxis	0.000787277	0.010272862	17/1052
536	BP	regulation of respiratory gaseous exchange	0.000793874	0.010297215	6/1052
537	BP	cell junction disassembly	0.000793874	0.010297215	6/1052
538	BP	regulation of systemic arterial blood pressure	0.00079541	0.010297215	14/1052
539	BP	excitatory postsynaptic potential	0.00079541	0.010297215	14/1052
540	BP	aging	0.000796519	0.010297215	20/1052
541	BP	neutrophil chemotaxis	0.00081033	0.010456402	15/1052
542	BP	positive regulation of cell projection organization	0.000812391	0.010463652	34/1052
543	BP	ensheathment of neurons	0.000825419	0.010592378	18/1052

544	BP	axon ensheathment	0.000825419	0.010592378	18/1052
545	BP	aortic valve development	0.000844807	0.010781711	8/1052
546	BP	positive regulation of adenylate cyclase activity	0.000844807	0.010781711	8/1052
547	BP	insulin-like growth factor receptor signaling pathway	0.000844807	0.010781711	8/1052
548	BP	fibroblast growth factor receptor signaling pathway	0.000847776	0.010799856	13/1052
549	BP	embryonic cranial skeleton morphogenesis	0.000877845	0.011073097	9/1052
550	BP	diencephalon development	0.000881453	0.011073097	12/1052
551	BP	regulation of glial cell differentiation	0.000881453	0.011073097	12/1052
552	BP	cell fate specification	0.00088423	0.011073097	14/1052
553	BP	somitogenesis	0.000885012	0.011073097	11/1052
554	BP	skeletal muscle cell differentiation	0.000885012	0.011073097	11/1052
555	BP	muscle adaptation	0.000885107	0.011073097	16/1052
556	BP	regulation of animal organ formation	0.000886673	0.011073097	7/1052
557	BP	response to pain	0.000886673	0.011073097	7/1052
558	BP	renal system vasculature development	0.000886673	0.011073097	7/1052
559	BP	kidney vasculature development	0.000886673	0.011073097	7/1052
560	BP	gonad development	0.000902069	0.01122521	25/1052
561	BP	response to carbohydrate	0.000902069	0.01122521	25/1052
562	BP	anterior/posterior pattern specification	0.000933817	0.011599601	24/1052
563	BP	regulation of vascular associated smooth muscle cell proliferation	0.00094859	0.011754916	13/1052
564	BP	negative regulation of hydrolase activity	0.000949688	0.011754916	36/1052
565	BP	regulation of endopeptidase activity	0.000966447	0.011929859	40/1052
566	BP	response to ethanol	0.000970658	0.011929859	16/1052
567	BP	positive regulation of protein kinase B signaling	0.000970658	0.011929859	16/1052
568	BP	positive regulation of calcium ion transport	0.000970658	0.011929859	16/1052
569	BP	skeletal myofibril assembly	0.00097896	0.011989679	4/1052

570	BP	establishment of blood-brain barrier	0.00097896	0.011989679	4/1052
571	BP	positive regulation of peptidyl-tyrosine phosphorylation	0.000982858	0.012016345	22/1052
572	BP	regulation of endothelial cell proliferation	0.000997114	0.01216932	21/1052
573	BP	endothelial cell migration	0.001001851	0.012205795	29/1052
574	BP	negative regulation of cell-substrate adhesion	0.001009266	0.012274718	11/1052
575	BP	cardiac left ventricle morphogenesis	0.001018168	0.012339986	5/1052
576	BP	negative regulation of myotube differentiation	0.001018168	0.012339986	5/1052
577	BP	cell recognition	0.001023811	0.012386873	25/1052
578	BP	axonal fasciculation	0.001040338	0.012435959	6/1052
579	BP	regulation of cardiac muscle contraction by regulation of the release of sequestered calcium ion	0.001040338	0.012435959	6/1052
580	BP	negative regulation of tissue remodeling	0.001040338	0.012435959	6/1052
581	BP	sympathetic nervous system development	0.001040338	0.012435959	6/1052
582	BP	cellular response to vitamin D	0.001040338	0.012435959	6/1052
583	BP	membrane depolarization during cardiac muscle cell action potential	0.001040338	0.012435959	6/1052
584	BP	neuron projection fasciculation	0.001040338	0.012435959	6/1052
585	BP	vascular associated smooth muscle cell proliferation	0.001059248	0.012640354	13/1052
586	BP	adenylate cyclase-activating G protein-coupled receptor signaling pathway	0.001076754	0.012805482	19/1052
587	BP	negative regulation of ion transport	0.001076754	0.012805482	19/1052
588	BP	bone morphogenesis	0.00108709	0.012906415	14/1052
589	BP	activation of adenylate cyclase activity	0.001101306	0.013030872	7/1052
590	BP	face morphogenesis	0.001101306	0.013030872	7/1052
591	BP	regulation of lyase activity	0.001119185	0.01322002	12/1052
592	BP	branching involved in ureteric bud morphogenesis	0.001126199	0.013280394	10/1052
593	BP	positive regulation of fat cell differentiation	0.001147572	0.013464196	11/1052
594	BP	cellular response to retinoic acid	0.001147572	0.013464196	11/1052

595	BP	regulation of sodium ion transmembrane transport	0.001147572	0.013464196	11/1052
596	BP	regulation of calcium ion transmembrane transport	0.001163345	0.013626363	19/1052
597	BP	bone remodeling	0.001180483	0.013803944	13/1052
598	BP	spinal cord development	0.001202332	0.013951981	14/1052
599	BP	acute-phase response	0.001208849	0.013951981	9/1052
600	BP	vasodilation	0.001208849	0.013951981	9/1052
601	BP	regulation of vascular permeability	0.001208849	0.013951981	9/1052
602	BP	autonomic nervous system development	0.001208849	0.013951981	9/1052
603	BP	face development	0.001208849	0.013951981	9/1052
604	BP	actin filament-based movement	0.00121473	0.013951981	17/1052
605	BP	regulation of cellular response to transforming growth factor beta stimulus	0.00121473	0.013951981	17/1052
606	BP	regulation of cellular extravasation	0.001219125	0.013951981	8/1052
607	BP	cyclic nucleotide metabolic process	0.001219125	0.013951981	8/1052
608	BP	response to progesterone	0.001219125	0.013951981	8/1052
609	BP	negative regulation of potassium ion transport	0.001219125	0.013951981	8/1052
610	BP	negative regulation of striated muscle cell differentiation	0.001219125	0.013951981	8/1052
611	BP	negative regulation of nervous system development	0.001244577	0.014219949	18/1052
612	BP	adenylate cyclase-inhibiting G protein-coupled receptor signaling pathway	0.00125665	0.014311053	12/1052
613	BP	regulation of cyclase activity	0.00125665	0.014311053	12/1052
614	BP	negative regulation of proteolysis	0.001272501	0.014467963	34/1052
615	BP	maintenance of location in cell	0.001287234	0.014611673	24/1052
616	BP	embryonic digit morphogenesis	0.001293774	0.01463831	10/1052
617	BP	regulation of adenylate cyclase activity	0.001293774	0.01463831	10/1052
618	BP	negative regulation of muscle cell differentiation	0.001301107	0.01467371	11/1052
619	BP	regulation of cell-substrate junction organization	0.001301107	0.01467371	11/1052
620	BP	amine transport	0.001327631	0.014948692	14/1052

621	BP	regulation of receptor binding	0.001341525	0.01508081	6/1052
622	BP	embryonic forelimb morphogenesis	0.00135466	0.01520399	7/1052
623	BP	regulation of cell-matrix adhesion	0.001386433	0.015535618	16/1052
624	BP	negative regulation of myeloid leukocyte differentiation	0.00140852	0.015734425	9/1052
625	BP	regulation of respiratory system process	0.001413191	0.015734425	5/1052
626	BP	behavioral response to pain	0.001413191	0.015734425	5/1052
627	BP	glomerular mesangium development	0.001413191	0.015734425	5/1052
628	BP	regulation of MAP kinase activity	0.001419176	0.015775903	21/1052
629	BP	regulation of lipid metabolic process	0.001437802	0.015957548	33/1052
630	BP	regulation of actin filament-based movement	0.001450265	0.016070319	8/1052
631	BP	regulation of organ growth	0.001463658	0.0161674	14/1052
632	BP	chemical synaptic transmission, postsynaptic	0.001463658	0.0161674	14/1052
633	BP	regulation of protein binding	0.001476891	0.016287793	22/1052
634	BP	neural crest cell migration	0.001481101	0.016308462	10/1052
635	BP	cardiac neural crest cell migration involved in outflow tract morphogenesis	0.001559528	0.017037657	4/1052
636	BP	tonic smooth muscle contraction	0.001559528	0.017037657	4/1052
637	BP	bone trabecula formation	0.001559528	0.017037657	4/1052
638	BP	cartilage morphogenesis	0.001559528	0.017037657	4/1052
639	BP	positive regulation of platelet aggregation	0.001559528	0.017037657	4/1052
640	BP	regulation of peptidyl-tyrosine phosphorylation	0.001575473	0.017184967	27/1052
641	BP	regulation of body fluid levels	0.001588035	0.017231562	36/1052
642	BP	cell migration involved in metanephros development	0.001604428	0.017231562	3/1052
643	BP	oviduct development	0.001604428	0.017231562	3/1052
644	BP	regulation of branching involved in prostate gland morphogenesis	0.001604428	0.017231562	3/1052
645	BP	cellular response to heparin	0.001604428	0.017231562	3/1052
646	BP	gephyrin clustering involved in postsynaptic density assembly	0.001604428	0.017231562	3/1052

647	BP	regulation of endodermal cell differentiation	0.001604428	0.017231562	3/1052
648	BP	response to bile acid	0.001604428	0.017231562	3/1052
649	BP	cellular response to bile acid	0.001604428	0.017231562	3/1052
650	BP	regulation of midbrain dopaminergic neuron differentiation	0.001604428	0.017231562	3/1052
651	BP	roof of mouth development	0.001615533	0.017324176	13/1052
652	BP	cAMP-mediated signaling	0.001633877	0.017494014	9/1052
653	BP	neural retina development	0.001658878	0.017707381	11/1052
654	BP	cranial skeletal system development	0.001658878	0.017707381	11/1052
655	BP	negative regulation of immune system process	0.001692478	0.01803846	39/1052
656	BP	retina development in camera-type eye	0.001698968	0.018080024	18/1052
657	BP	regulation of systemic arterial blood pressure by renin-angiotensin	0.001704935	0.01808838	6/1052
658	BP	cell differentiation involved in metanephros development	0.001704935	0.01808838	6/1052
659	BP	negative regulation of gliogenesis	0.001714844	0.018110933	8/1052
660	BP	positive regulation of heart growth	0.001714844	0.018110933	8/1052
661	BP	semi-lunar valve development	0.001714844	0.018110933	8/1052
662	BP	myelination	0.001824416	0.019239048	17/1052
663	BP	morphogenesis of embryonic epithelium	0.001832245	0.019263405	18/1052
664	BP	hindbrain development	0.001832245	0.019263405	18/1052
665	BP	positive regulation of phagocytosis	0.001865765	0.019556917	11/1052
666	BP	cardiac septum morphogenesis	0.001865765	0.019556917	11/1052
667	BP	response to vitamin A	0.00191053	0.019814891	5/1052
668	BP	collagen-activated signaling pathway	0.00191053	0.019814891	5/1052
669	BP	ganglion development	0.00191053	0.019814891	5/1052
670	BP	dendrite self-avoidance	0.00191053	0.019814891	5/1052
671	BP	epithelial cell-cell adhesion	0.00191053	0.019814891	5/1052
672	BP	postsynaptic density assembly	0.00191053	0.019814891	5/1052

673	BP	transforming growth factor beta receptor signaling pathway	0.001913084	0.019814891	22/1052
674	BP	response to monosaccharide	0.001913084	0.019814891	22/1052
675	BP	vasoconstriction	0.001953686	0.020205453	12/1052
676	BP	negative regulation of cell adhesion	0.001972221	0.020366974	30/1052
677	BP	mesodermal cell differentiation	0.001996128	0.020522779	7/1052
678	BP	leukocyte tethering or rolling	0.001996128	0.020522779	7/1052
679	BP	regulation of renal system process	0.001996128	0.020522779	7/1052
680	BP	oligodendrocyte development	0.002016139	0.020667644	8/1052
681	BP	positive regulation of smooth muscle cell migration	0.002016139	0.020667644	8/1052
682	BP	response to mechanical stimulus	0.002037831	0.020859378	22/1052
683	BP	regulation of reproductive process	0.002047238	0.020924989	20/1052
684	BP	nephron tubule morphogenesis	0.002093184	0.021363329	11/1052
685	BP	neutrophil migration	0.002108096	0.021484112	16/1052
686	BP	response to glucose	0.002118832	0.021562048	21/1052
687	BP	neuromuscular process	0.002125343	0.021596828	18/1052
688	BP	negative regulation of neurogenesis	0.002131621	0.021603796	17/1052
689	BP	synapse maturation	0.002138408	0.021603796	6/1052
690	BP	trabecula formation	0.002138408	0.021603796	6/1052
691	BP	positive regulation of excitatory postsynaptic potential	0.002138408	0.021603796	6/1052
692	BP	response to axon injury	0.002170155	0.021821802	12/1052
693	BP	SMAD protein signal transduction	0.002170155	0.021821802	12/1052
694	BP	negative regulation of leukocyte migration	0.002171015	0.021821802	9/1052
695	BP	morphogenesis of a polarized epithelium	0.002175616	0.021821802	13/1052
696	BP	cellular response to ketone	0.002175616	0.021821802	13/1052
697	BP	positive regulation of transporter activity	0.00223041	0.022339304	15/1052
698	BP	cardiac septum development	0.002330221	0.02309895	14/1052

699	BP	positive regulation of ion transmembrane transporter activity	0.002330221	0.02309895	14/1052
700	BP	cellular extravasation	0.002342603	0.02309895	11/1052
701	BP	regulation of dendritic cell antigen processing and presentation	0.002342652	0.02309895	4/1052
702	BP	noradrenergic neuron differentiation	0.002342652	0.02309895	4/1052
703	BP	short-term memory	0.002342652	0.02309895	4/1052
704	BP	sequestering of extracellular ligand from receptor	0.002342652	0.02309895	4/1052
705	BP	vitamin D receptor signaling pathway	0.002342652	0.02309895	4/1052
706	BP	presynaptic membrane organization	0.002342652	0.02309895	4/1052
707	BP	synapse pruning	0.002342652	0.02309895	4/1052
708	BP	negative regulation of neuron migration	0.002342652	0.02309895	4/1052
709	BP	somatic stem cell population maintenance	0.00235757	0.023213252	8/1052
710	BP	peptide hormone processing	0.002394083	0.023440527	7/1052
711	BP	retinoic acid metabolic process	0.002394083	0.023440527	7/1052
712	BP	head morphogenesis	0.002394083	0.023440527	7/1052
713	BP	cell-cell signaling involved in cardiac conduction	0.002394083	0.023440527	7/1052
714	BP	retinoid metabolic process	0.00240583	0.02345735	12/1052
715	BP	membrane depolarization	0.00240583	0.02345735	12/1052
716	BP	positive regulation of kinase activity	0.002405882	0.02345735	42/1052
717	BP	granulocyte migration	0.002456837	0.023910345	18/1052
718	BP	positive regulation of growth	0.00245985	0.023910345	26/1052
719	BP	ureteric bud morphogenesis	0.002462618	0.023910345	10/1052
720	BP	regulation of transforming growth factor beta receptor signaling pathway	0.002473531	0.023982946	16/1052
721	BP	intestinal cholesterol absorption	0.002524644	0.024410721	5/1052
722	BP	craniofacial suture morphogenesis	0.002524644	0.024410721	5/1052
723	BP	integrin-mediated signaling pathway	0.002546317	0.024586222	14/1052
724	BP	cellular response to vascular endothelial growth factor stimulus	0.002615547	0.025185013	11/1052

725	BP	nephron epithelium morphogenesis	0.002615547	0.025185013	11/1052
726	BP	regulation of the force of heart contraction	0.002650061	0.025342565	6/1052
727	BP	regulation of cardiac muscle contraction by calcium ion signaling	0.002650061	0.025342565	6/1052
728	BP	central nervous system projection neuron axonogenesis	0.002650061	0.025342565	6/1052
729	BP	regulation of ryanodine-sensitive calcium-release channel activity	0.002650061	0.025342565	6/1052
730	BP	apoptotic process involved in morphogenesis	0.002650061	0.025342565	6/1052
731	BP	response to hexose	0.002732031	0.026090705	21/1052
732	BP	positive regulation of glucose transmembrane transport	0.002742691	0.026121049	8/1052
733	BP	mammary gland morphogenesis	0.002742691	0.026121049	8/1052
734	BP	regulation of vasoconstriction	0.002775383	0.02631979	10/1052
735	BP	mesonephric tubule morphogenesis	0.002775383	0.02631979	10/1052
736	BP	response to monoamine	0.00277864	0.02631979	14/1052
737	BP	response to catecholamine	0.00277864	0.02631979	14/1052
738	BP	positive regulation of striated muscle cell differentiation	0.002840001	0.026817635	9/1052
739	BP	inositol lipid-mediated signaling	0.002847485	0.026817635	20/1052
740	BP	central nervous system neuron axonogenesis	0.002850406	0.026817635	7/1052
741	BP	response to vitamin D	0.002850406	0.026817635	7/1052
742	BP	embryonic eye morphogenesis	0.002850406	0.026817635	7/1052
743	BP	positive regulation of transmembrane receptor protein serine/threonine kinase signaling pathway	0.002861018	0.02688125	15/1052
744	BP	anatomical structure maturation	0.002922066	0.027417934	25/1052
745	BP	regulation of tissue remodeling	0.00293979	0.027547216	12/1052
746	BP	substrate adhesion-dependent cell spreading	0.003028088	0.027644988	14/1052
747	BP	Notch signaling pathway	0.00303553	0.027644988	20/1052
748	BP	tumor necrosis factor production	0.00303553	0.027644988	20/1052
749	BP	regulation of tumor necrosis factor production	0.00303553	0.027644988	20/1052

750	BP	corticospinal tract morphogenesis	0.003075325	0.027644988	3/1052
751	BP	skeletal muscle thin filament assembly	0.003075325	0.027644988	3/1052
752	BP	cell migration involved in kidney development	0.003075325	0.027644988	3/1052
753	BP	atrial cardiac muscle tissue morphogenesis	0.003075325	0.027644988	3/1052
754	BP	convergent extension involved in axis elongation	0.003075325	0.027644988	3/1052
755	BP	regulation of prostatic bud formation	0.003075325	0.027644988	3/1052
756	BP	regulation of presynaptic membrane potential	0.003075325	0.027644988	3/1052
757	BP	neural crest cell migration involved in autonomic nervous system development	0.003075325	0.027644988	3/1052
758	BP	acetate ester transport	0.003075325	0.027644988	3/1052
759	BP	negative regulation of non-canonical Wnt signaling pathway	0.003075325	0.027644988	3/1052
760	BP	regulation of hepatic stellate cell activation	0.003075325	0.027644988	3/1052
761	BP	telencephalon development	0.003083523	0.027644988	25/1052
762	BP	axon extension	0.003101314	0.027644988	15/1052
763	BP	heart valve development	0.003119087	0.027644988	10/1052
764	BP	regulation of endocytosis	0.003119637	0.027644988	22/1052
765	BP	regulation of complement activation, lectin pathway	0.003128426	0.027644988	2/1052
766	BP	negative regulation of complement activation, lectin pathway	0.003128426	0.027644988	2/1052
767	BP	cerebral cortex GABAergic interneuron fate commitment	0.003128426	0.027644988	2/1052
768	BP	corticospinal neuron axon guidance	0.003128426	0.027644988	2/1052
769	BP	embryonic genitalia morphogenesis	0.003128426	0.027644988	2/1052
770	BP	positive regulation of epinephrine secretion	0.003128426	0.027644988	2/1052
771	BP	regulation of lipoprotein oxidation	0.003128426	0.027644988	2/1052
772	BP	Wnt signaling pathway involved in dorsal/ventral axis specification	0.003128426	0.027644988	2/1052
773	BP	regulation of lipoprotein lipid oxidation	0.003128426	0.027644988	2/1052
774	BP	hepatic stellate cell migration	0.003128426	0.027644988	2/1052
775	BP	regulation of hepatic stellate cell migration	0.003128426	0.027644988	2/1052

776	BP	positive regulation of hepatic stellate cell migration	0.003128426	0.027644988	2/1052
777	BP	negative regulation of vitamin D receptor signaling pathway	0.003128426	0.027644988	2/1052
778	BP	phospholipase C-activating angiotensin-activated signaling pathway	0.003128426	0.027644988	2/1052
779	BP	dorsal spinal cord interneuron axon guidance	0.003128426	0.027644988	2/1052
780	BP	retrograde trans-synaptic signaling by soluble gas	0.003128426	0.027644988	2/1052
781	BP	retrograde trans-synaptic signaling by nitric oxide	0.003128426	0.027644988	2/1052
782	BP	synaptic signaling by nitric oxide	0.003128426	0.027644988	2/1052
783	BP	trans-synaptic signaling by soluble gas	0.003128426	0.027644988	2/1052
784	BP	trans-synaptic signaling by nitric oxide	0.003128426	0.027644988	2/1052
785	BP	positive regulation of amacrine cell differentiation	0.003128426	0.027644988	2/1052
786	BP	regulation of planar cell polarity pathway involved in axis elongation	0.003128426	0.027644988	2/1052
787	BP	negative regulation of planar cell polarity pathway involved in axis elongation	0.003128426	0.027644988	2/1052
788	BP	regulation of Wnt signaling pathway involved in dorsal/ventral axis specification	0.003128426	0.027644988	2/1052
789	BP	negative regulation of Wnt signaling pathway involved in dorsal/ventral axis specification	0.003128426	0.027644988	2/1052
790	BP	negative regulation of smooth muscle cell-matrix adhesion	0.003128426	0.027644988	2/1052
791	BP	heart valve morphogenesis	0.003230526	0.028349833	9/1052
792	BP	positive regulation of phospholipase activity	0.003230526	0.028349833	9/1052
793	BP	macrophage migration	0.003230526	0.028349833	9/1052
794	BP	adult locomotory behavior	0.003238376	0.028349833	11/1052
795	BP	negative regulation of wound healing	0.003238376	0.028349833	11/1052
796	BP	renal tubule morphogenesis	0.003238376	0.028349833	11/1052
797	BP	diterpenoid metabolic process	0.003240677	0.028349833	12/1052
798	BP	defense response to Gram-negative bacterium	0.003240677	0.028349833	12/1052
799	BP	negative regulation of glial cell differentiation	0.003248241	0.028380441	6/1052
800	BP	parasympathetic nervous system development	0.003270205	0.028536623	5/1052
801	BP	positive regulation of neurogenesis	0.003308002	0.028830415	23/1052

802	BP	camera-type eye morphogenesis	0.003357965	0.029012646	15/1052
803	BP	complement activation, lectin pathway	0.003359388	0.029012646	4/1052
804	BP	collagen-activated tyrosine kinase receptor signaling pathway	0.003359388	0.029012646	4/1052
805	BP	positive regulation of insulin-like growth factor receptor signaling pathway	0.003359388	0.029012646	4/1052
806	BP	negative regulation of pathway-restricted SMAD protein phosphorylation	0.003359388	0.029012646	4/1052
807	BP	cardiac neural crest cell development involved in outflow tract morphogenesis	0.003359388	0.029012646	4/1052
808	BP	fibroblast activation	0.003359388	0.029012646	4/1052
809	BP	response to purine-containing compound	0.003363134	0.029012646	16/1052
810	BP	peptide cross-linking	0.003370471	0.029012646	7/1052
811	BP	signaling receptor ligand precursor processing	0.003370471	0.029012646	7/1052
812	BP	epithelial cell migration	0.003440163	0.029576081	33/1052
813	BP	regulation of phospholipase activity	0.003495841	0.03001779	10/1052
814	BP	hormone transport	0.003555659	0.030493929	28/1052
815	BP	regulation of phosphatidylinositol 3-kinase signaling	0.003582087	0.030613901	14/1052
816	BP	cellular response to interleukin-1	0.003582087	0.030613901	14/1052
817	BP	neuron projection extension	0.003590391	0.030613901	19/1052
818	BP	positive regulation of axonogenesis	0.003591575	0.030613901	11/1052
819	BP	regulation of cardiac muscle contraction	0.003591575	0.030613901	11/1052
820	BP	ventral spinal cord development	0.003658831	0.031025591	8/1052
821	BP	trabecula morphogenesis	0.003658831	0.031025591	8/1052
822	BP	ventricular cardiac muscle tissue development	0.003662095	0.031025591	9/1052
823	BP	regulation of catecholamine secretion	0.003662095	0.031025591	9/1052
824	BP	sodium ion homeostasis	0.003662095	0.031025591	9/1052
825	BP	regulation of synaptic plasticity	0.003702277	0.031327998	21/1052
826	BP	stem cell differentiation	0.003712812	0.031379106	22/1052
827	BP	regulation of gliogenesis	0.003775863	0.031873399	13/1052

828	BP	regulation of signaling receptor activity	0.003827891	0.032273561	19/1052
829	BP	anatomical structure homeostasis	0.003844664	0.032375875	30/1052
830	BP	sprouting angiogenesis	0.003894854	0.032747477	20/1052
831	BP	epithelial tube formation	0.003898174	0.032747477	16/1052
832	BP	epithelium migration	0.003911208	0.032778085	33/1052
833	BP	cellular response to peptide	0.003911208	0.032778085	33/1052
834	BP	response to organophosphorus	0.003923627	0.03284273	15/1052
835	BP	neurotransmitter metabolic process	0.003941468	0.032952558	6/1052
836	BP	specification of animal organ identity	0.003959792	0.033026655	7/1052
837	BP	positive regulation of cardiac muscle tissue growth	0.003959792	0.033026655	7/1052
838	BP	positive regulation of synaptic transmission	0.003976377	0.033125403	18/1052
839	BP	catecholamine secretion	0.004137576	0.034262339	9/1052
840	BP	regulation of action potential	0.004137576	0.034262339	9/1052
841	BP	tumor necrosis factor superfamily cytokine production	0.004138693	0.034262339	20/1052
842	BP	regulation of tumor necrosis factor superfamily cytokine production	0.004138693	0.034262339	20/1052
843	BP	cartilage condensation	0.004161934	0.034262339	5/1052
844	BP	regulation of antigen processing and presentation	0.004161934	0.034262339	5/1052
845	BP	wound healing, spreading of epidermal cells	0.004161934	0.034262339	5/1052
846	BP	regulation of renal sodium excretion	0.004161934	0.034262339	5/1052
847	BP	middle ear morphogenesis	0.004161934	0.034262339	5/1052
848	BP	regulation of cell growth involved in cardiac muscle cell development	0.004161934	0.034262339	5/1052
849	BP	cellular hormone metabolic process	0.004190781	0.03435285	16/1052
850	BP	endocardial cushion development	0.004197533	0.03435285	8/1052
851	BP	negative chemotaxis	0.004197533	0.03435285	8/1052
852	BP	ventricular cardiac muscle tissue morphogenesis	0.004197533	0.03435285	8/1052
853	BP	regulation of calcium ion import	0.004197533	0.03435285	8/1052

854	BP	glucose transmembrane transport	0.00421604	0.034463906	14/1052
855	BP	odontogenesis of dentin-containing tooth	0.004295422	0.03502972	12/1052
856	BP	regulation of lipase activity	0.004295422	0.03502972	12/1052
857	BP	cellular response to peptide hormone stimulus	0.004300311	0.03502972	28/1052
858	BP	positive regulation of lipase activity	0.004357251	0.035452175	10/1052
859	BP	negative regulation of leukocyte differentiation	0.004483531	0.036437173	13/1052
860	BP	negative regulation of transport	0.004493941	0.036479308	40/1052
861	BP	renal system process	0.004565527	0.037017353	14/1052
862	BP	positive regulation of supramolecular fiber organization	0.004619769	0.037135627	19/1052
863	BP	positive regulation of catecholamine secretion	0.004639417	0.037135627	4/1052
864	BP	negative regulation of osteoblast proliferation	0.004639417	0.037135627	4/1052
865	BP	regulation of cell fate specification	0.004639417	0.037135627	4/1052
866	BP	positive regulation of phagocytosis, engulfment	0.004639417	0.037135627	4/1052
867	BP	bone trabecula morphogenesis	0.004639417	0.037135627	4/1052
868	BP	leukocyte aggregation	0.004639417	0.037135627	4/1052
869	BP	GABAergic neuron differentiation	0.004639417	0.037135627	4/1052
870	BP	extracellular regulation of signal transduction	0.004639417	0.037135627	4/1052
871	BP	extracellular negative regulation of signal transduction	0.004639417	0.037135627	4/1052
872	BP	positive regulation of membrane invagination	0.004639417	0.037135627	4/1052
873	BP	morphogenesis of an epithelial sheet	0.004659907	0.037135627	9/1052
874	BP	vascular endothelial growth factor receptor signaling pathway	0.004659907	0.037135627	9/1052
875	BP	cardiac muscle cell proliferation	0.004659907	0.037135627	9/1052
876	BP	positive regulation of wound healing	0.004659907	0.037135627	9/1052
877	BP	regulation of calcium ion transmembrane transporter activity	0.004702493	0.037432277	12/1052
878	BP	positive regulation of filopodium assembly	0.004738371	0.037632044	6/1052
879	BP	positive regulation of synaptic transmission, glutamatergic	0.004738371	0.037632044	6/1052

880	BP	fibroblast migration	0.00479527	0.037954401	8/1052
881	BP	regulation of cell adhesion mediated by integrin	0.00479527	0.037954401	8/1052
882	BP	epithelial cell differentiation involved in kidney development	0.00479527	0.037954401	8/1052
883	BP	regulation of binding	0.004817853	0.03808996	33/1052
884	BP	central nervous system neuron development	0.004839208	0.038215509	11/1052
885	BP	cellular response to molecule of bacterial origin	0.004849108	0.038250418	23/1052
886	BP	inner ear morphogenesis	0.004875381	0.038414259	13/1052
887	BP	temperature homeostasis	0.004911965	0.038615348	19/1052
888	BP	phosphatidylinositol-mediated signaling	0.004911965	0.038615348	19/1052
889	BP	negative regulation of vasculature development	0.005021014	0.039428235	17/1052
890	BP	negative regulation of response to wounding	0.005139798	0.039924204	12/1052
891	BP	luteolysis	0.005158521	0.039924204	3/1052
892	BP	response to insecticide	0.005158521	0.039924204	3/1052
893	BP	anterior/posterior axon guidance	0.005158521	0.039924204	3/1052
894	BP	autocrine signaling	0.005158521	0.039924204	3/1052
895	BP	paracrine signaling	0.005158521	0.039924204	3/1052
896	BP	micturition	0.005158521	0.039924204	3/1052
897	BP	mammary gland formation	0.005158521	0.039924204	3/1052
898	BP	mesenchymal-epithelial cell signaling	0.005158521	0.039924204	3/1052
899	BP	odontoblast differentiation	0.005158521	0.039924204	3/1052
900	BP	positive regulation of microglial cell migration	0.005158521	0.039924204	3/1052
901	BP	dorsal root ganglion development	0.005158521	0.039924204	3/1052
902	BP	negative regulation of dendritic cell apoptotic process	0.005158521	0.039924204	3/1052
903	BP	smoothened signaling pathway	0.00517816	0.040031819	16/1052
904	BP	regulation of complement activation	0.005214453	0.040134616	5/1052
905	BP	lipid digestion	0.005214453	0.040134616	5/1052

906	BP	regulation of branching involved in ureteric bud morphogenesis	0.005214453	0.040134616	5/1052
907	BP	intestinal lipid absorption	0.005214453	0.040134616	5/1052
908	BP	syncytium formation by plasma membrane fusion	0.005232092	0.040137619	9/1052
909	BP	negative regulation of fat cell differentiation	0.005232092	0.040137619	9/1052
910	BP	cell-cell fusion	0.005232092	0.040137619	9/1052
911	BP	negative regulation of transforming growth factor beta receptor signaling pathway	0.005323833	0.040796574	11/1052
912	BP	negative regulation of smooth muscle cell migration	0.005368818	0.041051172	7/1052
913	BP	modulation of excitatory postsynaptic potential	0.005368818	0.041051172	7/1052
914	BP	regulation of hormone secretion	0.005385642	0.041134757	23/1052
915	BP	regulation of bone remodeling	0.0054561	0.041581911	8/1052
916	BP	neuromuscular process controlling balance	0.0054561	0.041581911	8/1052
917	BP	cell-cell junction organization	0.005529507	0.042095406	21/1052
918	BP	response to interleukin-1	0.005546466	0.04217852	16/1052
919	BP	positive regulation of macrophage activation	0.005647637	0.042807985	6/1052
920	BP	negative regulation of osteoclast differentiation	0.005647637	0.042807985	6/1052
921	BP	regulation of cardiac muscle cell membrane repolarization	0.005647637	0.042807985	6/1052
922	BP	regulation of axonogenesis	0.005723488	0.043330899	17/1052
923	BP	primary alcohol metabolic process	0.005741453	0.043330899	13/1052
924	BP	cellular response to monoamine stimulus	0.005741453	0.043330899	13/1052
925	BP	cellular response to catecholamine stimulus	0.005741453	0.043330899	13/1052
926	BP	hexose transmembrane transport	0.005756744	0.043399382	14/1052
927	BP	positive regulation of response to external stimulus	0.005768089	0.043438005	38/1052
928	BP	negative regulation of synaptic transmission	0.00595341	0.044785294	10/1052
929	BP	negative regulation of cell-matrix adhesion	0.006200026	0.046180738	7/1052
930	BP	cardiac muscle cell membrane repolarization	0.006200026	0.046180738	7/1052
931	BP	cardiac chamber formation	0.00621063	0.046180738	4/1052

932	BP	protein oxidation	0.00621063	0.046180738	4/1052
933	BP	platelet-derived growth factor receptor-beta signaling pathway	0.00621063	0.046180738	4/1052
934	BP	angiotensin-activated signaling pathway	0.00621063	0.046180738	4/1052
935	BP	neurotransmitter biosynthetic process	0.00621063	0.046180738	4/1052
936	BP	detection of mechanical stimulus involved in sensory perception of pain	0.00621063	0.046180738	4/1052
937	BP	regulation of vascular wound healing	0.00621063	0.046180738	4/1052
938	BP	neurotransmitter-gated ion channel clustering	0.00621063	0.046180738	4/1052
939	BP	regulation of actin filament bundle assembly	0.006218292	0.046180738	13/1052
940	BP	negative regulation of hemopoiesis	0.006218292	0.046180738	13/1052
941	BP	monoamine transport	0.006407551	0.047389396	11/1052
942	BP	cyclic nucleotide biosynthetic process	0.006442134	0.047389396	5/1052
943	BP	cell differentiation in hindbrain	0.006442134	0.047389396	5/1052
944	BP	ionotropic glutamate receptor signaling pathway	0.006442134	0.047389396	5/1052
945	BP	renal sodium excretion	0.006442134	0.047389396	5/1052
946	BP	cyclic purine nucleotide metabolic process	0.006442134	0.047389396	5/1052
947	BP	hepatocyte proliferation	0.006442134	0.047389396	5/1052
948	BP	epithelial cell proliferation involved in liver morphogenesis	0.006442134	0.047389396	5/1052
949	BP	ligand-gated ion channel signaling pathway	0.006442134	0.047389396	5/1052
950	BP	forebrain cell migration	0.006538319	0.048046319	9/1052
951	BP	striated muscle cell proliferation	0.006576027	0.048171294	10/1052
952	BP	tissue regeneration	0.006576027	0.048171294	10/1052
953	BP	positive regulation of response to wounding	0.006576027	0.048171294	10/1052
954	BP	regulation of muscle adaptation	0.006648685	0.048601542	12/1052
955	BP	positive regulation of smooth muscle cell proliferation	0.006648685	0.048601542	12/1052
956	BP	endothelial cell chemotaxis	0.006677952	0.048684148	6/1052
957	BP	bone growth	0.006677952	0.048684148	6/1052

958	BP	monosaccharide transmembrane transport	0.006680907	0.048684148	14/1052
959	BP	phosphatidylinositol 3-kinase signaling	0.006780563	0.04935882	16/1052
1	CC	collagen-containing extracellular matrix	2.16E-62	1.38E-59	133/1114
2	CC	contractile fiber	9.46E-19	3.02E-16	54/1114
3	CC	myofibril	3.30E-18	7.03E-16	52/1114
4	CC	sarcomere	9.29E-18	1.48E-15	49/1114
5	CC	collagen trimer	1.88E-17	2.40E-15	31/1114
6	CC	sarcolemma	1.52E-16	1.61E-14	37/1114
7	CC	I band	1.96E-16	1.79E-14	38/1114
8	CC	Z disc	3.43E-16	2.74E-14	36/1114
9	CC	synaptic membrane	4.35E-16	3.09E-14	65/1114
10	CC	endoplasmic reticulum lumen	7.53E-16	4.81E-14	58/1114
11	CC	neuronal cell body	4.38E-14	2.55E-12	72/1114
12	CC	postsynaptic membrane	1.18E-13	6.28E-12	50/1114
13	CC	platelet alpha granule	1.56E-10	7.65E-09	24/1114
14	CC	basement membrane	4.10E-10	1.87E-08	24/1114
15	CC	perikaryon	5.01E-10	2.13E-08	31/1114
16	CC	platelet alpha granule lumen	3.35E-09	1.34E-07	19/1114
17	CC	intrinsic component of synaptic membrane	6.50E-09	2.44E-07	30/1114
18	CC	complex of collagen trimers	7.55E-09	2.68E-07	11/1114
19	CC	postsynaptic specialization	1.66E-08	5.59E-07	47/1114
20	CC	cation channel complex	3.54E-08	1.13E-06	35/1114
21	CC	secretory granule lumen	6.13E-08	1.86E-06	44/1114
22	CC	cytoplasmic vesicle lumen	8.04E-08	2.34E-06	44/1114
23	CC	intrinsic component of postsynaptic membrane	9.50E-08	2.50E-06	24/1114

24	CC	vesicle lumen	9.62E-08	2.50E-06	44/1114
25	CC	ion channel complex	9.78E-08	2.50E-06	41/1114
26	CC	postsynaptic specialization membrane	1.29E-07	3.17E-06	23/1114
27	CC	integral component of synaptic membrane	2.90E-07	6.87E-06	26/1114
28	CC	postsynaptic density	3.14E-07	7.16E-06	42/1114
29	CC	asymmetric synapse	4.80E-07	1.06E-05	42/1114
30	CC	neuron to neuron synapse	5.23E-07	1.11E-05	44/1114
31	CC	elastic fiber	5.89E-07	1.19E-05	5/1114
32	CC	T-tubule	6.06E-07	1.19E-05	14/1114
33	CC	caveola	6.14E-07	1.19E-05	18/1114
34	CC	integral component of postsynaptic membrane	7.32E-07	1.38E-05	22/1114
35	CC	integral component of postsynaptic specialization membrane	1.15E-06	2.00E-05	17/1114
36	CC	fibrillar collagen trimer	1.16E-06	2.00E-05	7/1114
37	CC	banded collagen fibril	1.16E-06	2.00E-05	7/1114
38	CC	plasma membrane raft	1.37E-06	2.31E-05	21/1114
39	CC	intrinsic component of postsynaptic specialization membrane	2.02E-06	3.32E-05	17/1114
40	CC	transmembrane transporter complex	2.09E-06	3.33E-05	45/1114
41	CC	glutamatergic synapse	2.26E-06	3.52E-05	40/1114
42	CC	protein complex involved in cell adhesion	3.19E-06	4.85E-05	12/1114
43	CC	cell-cell junction	3.79E-06	5.63E-05	54/1114
44	CC	transporter complex	4.16E-06	6.04E-05	46/1114
45	CC	presynaptic membrane	5.82E-06	8.26E-05	23/1114
46	CC	presynapse	5.96E-06	8.28E-05	53/1114
47	CC	main axon	6.19E-06	8.42E-05	14/1114
48	CC	actin filament bundle	6.53E-06	8.69E-05	16/1114
49	CC	GABA-ergic synapse	7.19E-06	9.37E-05	15/1114

50	CC	potassium channel complex	9.32E-06	0.000119103	17/1114
51	CC	membrane raft	2.18E-05	0.000273116	38/1114
52	CC	microfibril	2.29E-05	0.000281225	6/1114
53	CC	membrane microdomain	2.33E-05	0.000281479	38/1114
54	CC	stress fiber	2.78E-05	0.000322947	14/1114
55	CC	contractile actin filament bundle	2.78E-05	0.000322947	14/1114
56	CC	sarcoplasm	2.84E-05	0.000324023	15/1114
57	CC	postsynaptic density membrane	3.72E-05	0.000417025	16/1114
58	CC	sarcoplasmic reticulum	3.89E-05	0.000421518	14/1114
59	CC	cell-cell contact zone	3.89E-05	0.000421518	14/1114
60	CC	insulin-like growth factor binding protein complex	4.96E-05	0.000528461	4/1114
61	CC	intrinsic component of presynaptic membrane	6.29E-05	0.000658675	14/1114
62	CC	sodium channel complex	6.63E-05	0.000682867	8/1114
63	CC	intercalated disc	7.55E-05	0.00075756	11/1114
64	CC	axon initial segment	7.59E-05	0.00075756	7/1114
65	CC	anchored component of membrane	8.95E-05	0.000880083	23/1114
66	CC	actomyosin	9.88E-05	0.000956545	14/1114
67	CC	cell-substrate junction	0.000108124	0.001031214	44/1114
68	CC	dendritic spine	0.000117385	0.001103071	23/1114
69	CC	neuron spine	0.000128252	0.001187721	23/1114
70	CC	protein complex involved in cell-matrix adhesion	0.000142128	0.001297421	4/1114
71	CC	costamere	0.000162844	0.001465592	6/1114
72	CC	anchored component of plasma membrane	0.00016527	0.001466773	12/1114
73	CC	interstitial matrix	0.00020384	0.0017843	5/1114
74	CC	dystrophin-associated glycoprotein complex	0.000239595	0.002068937	6/1114
75	CC	growth factor complex	0.000316657	0.002697916	4/1114

76	CC	cation-transporting ATPase complex	0.000342173	0.002876953	6/1114
77	CC	distal axon	0.000355445	0.00294973	30/1114
78	CC	dendrite membrane	0.000403715	0.003307356	9/1114
79	CC	voltage-gated potassium channel complex	0.000480765	0.00388872	13/1114
80	CC	focal adhesion	0.000509508	0.004069697	41/1114
81	CC	fibrinogen complex	0.000604775	0.004770999	4/1114
82	CC	anchored component of external side of plasma membrane	0.000647717	0.005047455	6/1114
83	CC	blood microparticle	0.000681578	0.005247333	19/1114
84	CC	kainate selective glutamate receptor complex	0.000702047	0.005277745	3/1114
85	CC	insulin-like growth factor ternary complex	0.000702047	0.005277745	3/1114
86	CC	platelet dense granule lumen	0.00076522	0.005685762	5/1114
87	CC	Golgi lumen	0.000779964	0.005728698	15/1114
88	CC	platelet dense granule	0.000863506	0.006199783	6/1114
89	CC	glycoprotein complex	0.000863506	0.006199783	6/1114
90	CC	network-forming collagen trimer	0.001039637	0.00730031	4/1114
91	CC	collagen network	0.001039637	0.00730031	4/1114
92	CC	node of Ranvier	0.001094374	0.007601143	5/1114
93	CC	integral component of presynaptic membrane	0.001306038	0.008973746	11/1114
94	CC	integrin complex	0.001486218	0.010103117	7/1114
95	CC	intrinsic component of external side of plasma membrane	0.001850113	0.012314814	6/1114
96	CC	ATPase dependent transmembrane transport complex	0.001850113	0.012314814	6/1114
97	CC	voltage-gated sodium channel complex	0.002050381	0.01323428	5/1114
98	CC	platelet alpha granule membrane	0.002050381	0.01323428	5/1114
99	CC	intrinsic component of presynaptic active zone membrane	0.002050381	0.01323428	5/1114
100	CC	integral component of postsynaptic density membrane	0.002105663	0.013455188	9/1114
101	CC	vacuolar lumen	0.002153797	0.013626499	20/1114

102	CC	glial cell projection	0.002186498	0.013697769	7/1114
103	CC	anchored component of synaptic membrane	0.002484174	0.015411527	4/1114
104	CC	neuron projection terminus	0.002680778	0.016471317	16/1114
105	CC	intrinsic component of postsynaptic density membrane	0.003161002	0.018263278	9/1114
106	CC	FACIT collagen trimer	0.003219247	0.018263278	3/1114
107	CC	sarcoglycan complex	0.003219247	0.018263278	3/1114
108	CC	collagen type I trimer	0.003229656	0.018263278	2/1114
109	CC	collagen type VI trimer	0.003229656	0.018263278	2/1114
110	CC	short-chain collagen trimer	0.003229656	0.018263278	2/1114
111	CC	sheet-forming collagen trimer	0.003229656	0.018263278	2/1114
112	CC	collagen sheet	0.003229656	0.018263278	2/1114
113	CC	collagen beaded filament	0.003229656	0.018263278	2/1114
114	CC	lysosomal lumen	0.003311223	0.018560277	13/1114
115	CC	adherens junction	0.004267688	0.023713499	19/1114
116	CC	actin cytoskeleton	0.004431117	0.024409343	43/1114
117	CC	axon terminus	0.004851694	0.026497712	14/1114
118	CC	filamentous actin	0.00512193	0.027503474	6/1114
119	CC	presynaptic active zone membrane	0.00512193	0.027503474	6/1114
120	CC	neuron projection membrane	0.005169937	0.027529917	9/1114
121	CC	dystroglycan complex	0.00539619	0.028263652	3/1114
122	CC	anchored component of presynaptic membrane	0.00539619	0.028263652	3/1114
123	CC	ionotropic glutamate receptor complex	0.006754039	0.035088057	7/1114
124	CC	cell body membrane	0.00720744	0.037141567	6/1114
125	CC	Schaffer collateral - CA1 synapse	0.007332427	0.037483369	10/1114
126	CC	excitatory synapse	0.007665628	0.038875684	8/1114
127	CC	sarcoplasmic reticulum membrane	0.00775394	0.039013916	7/1114

128	CC	paranodal junction	0.00827104	0.041290581	3/1114
129	CC	guanylate cyclase complex, soluble	0.009322352	0.044455098	2/1114
130	CC	anchoring collagen complex	0.009322352	0.044455098	2/1114
131	CC	potassium ion-transporting ATPase complex	0.009322352	0.044455098	2/1114
132	CC	alphav-beta3 integrin-vitronectin complex	0.009322352	0.044455098	2/1114
133	CC	anchored component of presynaptic active zone membrane	0.009322352	0.044455098	2/1114
134	CC	GLI-SUFU complex	0.009322352	0.044455098	2/1114
1	MF	extracellular matrix structural constituent	1.23E-43	1.49E-40	72/1066
2	MF	glycosaminoglycan binding	2.08E-26	1.26E-23	64/1066
3	MF	heparin binding	1.10E-22	4.44E-20	50/1066
4	MF	collagen binding	2.11E-15	6.38E-13	26/1066
5	MF	receptor ligand activity	7.43E-15	1.54E-12	75/1066
6	MF	sulfur compound binding	7.67E-15	1.54E-12	52/1066
7	MF	signaling receptor activator activity	1.60E-14	2.76E-12	75/1066
8	MF	integrin binding	1.12E-13	1.70E-11	37/1066
9	MF	extracellular matrix structural constituent conferring tensile strength	1.92E-13	2.58E-11	19/1066
10	MF	growth factor binding	4.31E-13	5.21E-11	34/1066
11	MF	growth factor activity	8.08E-10	8.74E-08	32/1066
12	MF	extracellular matrix binding	8.68E-10	8.74E-08	18/1066
13	MF	fibronectin binding	5.94E-09	5.52E-07	13/1066
14	MF	insulin-like growth factor binding	2.77E-08	2.39E-06	12/1066
15	MF	G protein-coupled receptor binding	2.48E-07	1.99E-05	40/1066
16	MF	gated channel activity	4.78E-07	3.61E-05	44/1066
17	MF	G protein-coupled peptide receptor activity	1.27E-06	9.01E-05	25/1066
18	MF	potassium channel activity	1.53E-06	9.73E-05	22/1066

19	MF	voltage-gated ion channel activity	1.73E-06	9.73E-05	30/1066
20	MF	voltage-gated channel activity	1.73E-06	9.73E-05	30/1066
21	MF	cation channel activity	1.77E-06	9.73E-05	43/1066
22	MF	extracellular matrix structural constituent conferring compression resistance	1.77E-06	9.73E-05	9/1066
23	MF	cytokine activity	2.14E-06	0.000112506	33/1066
24	MF	peptide receptor activity	2.68E-06	0.000129708	25/1066
25	MF	extracellular matrix constituent conferring elasticity	2.68E-06	0.000129708	6/1066
26	MF	peptidase regulator activity	3.72E-06	0.000172658	32/1066
27	MF	ion channel regulator activity	4.22E-06	0.000188767	23/1066
28	MF	transmembrane receptor protein tyrosine kinase activity	6.19E-06	0.000267194	14/1066
29	MF	hormone activity	6.49E-06	0.000270421	21/1066
30	MF	channel regulator activity	7.79E-06	0.000313845	23/1066
31	MF	potassium ion transmembrane transporter activity	8.69E-06	0.000338594	24/1066
32	MF	ion channel activity	9.79E-06	0.00036959	49/1066
33	MF	serine-type endopeptidase inhibitor activity	1.16E-05	0.000425377	18/1066
34	MF	platelet-derived growth factor binding	1.33E-05	0.000460671	6/1066
35	MF	structural molecule activity conferring elasticity	1.33E-05	0.000460671	6/1066
36	MF	metalloendopeptidase activity	1.92E-05	0.000642946	19/1066
37	MF	adenylate cyclase binding	2.54E-05	0.000828741	6/1066
38	MF	voltage-gated cation channel activity	2.79E-05	0.000887037	22/1066
39	MF	peptidase inhibitor activity	2.98E-05	0.000922648	26/1066
40	MF	Wnt-protein binding	3.35E-05	0.001010544	9/1066
41	MF	hormone binding	3.46E-05	0.001018086	16/1066
42	MF	transmembrane receptor protein kinase activity	4.09E-05	0.001176183	15/1066
43	MF	cytokine binding	6.17E-05	0.00173404	21/1066
44	MF	channel activity	6.77E-05	0.001857383	50/1066

45	MF	passive transmembrane transporter activity	7.12E-05	0.001912233	50/1066
46	MF	metal ion transmembrane transporter activity	8.07E-05	0.00211806	45/1066
47	MF	structural constituent of muscle	0.000109163	0.002805721	10/1066
48	MF	endopeptidase inhibitor activity	0.000116123	0.002922429	24/1066
49	MF	alpha-actinin binding	0.000135097	0.003330562	8/1066
50	MF	endopeptidase regulator activity	0.000146601	0.003541888	25/1066
51	MF	peptide hormone binding	0.000156924	0.003716953	11/1066
52	MF	actinin binding	0.000160813	0.003735817	9/1066
53	MF	neuropeptide receptor activity	0.000202047	0.004605145	10/1066
54	MF	voltage-gated potassium channel activity	0.000217139	0.004857473	15/1066
55	MF	sodium channel activity	0.000244926	0.005315874	10/1066
56	MF	metallopeptidase activity	0.000246918	0.005315874	24/1066
57	MF	frizzled binding	0.000250832	0.005315874	9/1066
58	MF	scavenger receptor activity	0.00029517	0.006147678	10/1066
59	MF	fibroblast growth factor receptor binding	0.000409069	0.008375521	7/1066
60	MF	peptide hormone receptor binding	0.000524789	0.010565756	6/1066
61	MF	insulin-like growth factor II binding	0.000648277	0.012838013	4/1066
62	MF	copper ion binding	0.000674319	0.013138338	11/1066
63	MF	oxidoreductase activity, acting on the aldehyde or oxo group of donors, oxygen as acceptor	0.000740948	0.014207393	3/1066
64	MF	outward rectifier potassium channel activity	0.00083145	0.015693616	5/1066
65	MF	extracellular ligand-gated ion channel activity	0.000926799	0.017224204	12/1066
66	MF	carbohydrate binding	0.001012017	0.018184158	29/1066
67	MF	cell adhesion mediator activity	0.001023612	0.018184158	11/1066
68	MF	postsynaptic neurotransmitter receptor activity	0.001023612	0.018184158	11/1066
69	MF	transmembrane transporter binding	0.001047962	0.018346938	17/1066

70	MF	transmitter-gated ion channel activity involved in regulation of postsynaptic membrane potential	0.001117847	0.019290849	9/1066
71	MF	muscle alpha-actinin binding	0.001188046	0.020213516	5/1066
72	MF	serine-type peptidase activity	0.00162591	0.026546683	22/1066
73	MF	3',5'-cyclic-AMP phosphodiesterase activity	0.001646266	0.026546683	5/1066
74	MF	metalloendopeptidase inhibitor activity	0.001646266	0.026546683	5/1066
75	MF	actin binding	0.001765663	0.026546683	41/1066
76	MF	kainate selective glutamate receptor activity	0.001772379	0.026546683	3/1066
77	MF	CCR1 chemokine receptor binding	0.001772379	0.026546683	3/1066
78	MF	FATZ binding	0.001772379	0.026546683	3/1066
79	MF	voltage-gated sodium channel activity involved in cardiac muscle cell action potential	0.001772379	0.026546683	3/1066
80	MF	neuroligin family protein binding	0.001772379	0.026546683	3/1066
81	MF	neurotransmitter receptor activity involved in regulation of postsynaptic membrane potential	0.001784258	0.026546683	9/1066
82	MF	sodium channel regulator activity	0.00180201	0.026546683	8/1066
83	MF	neurotransmitter receptor activity	0.001832832	0.02667543	15/1066
84	MF	cargo receptor activity	0.001878042	0.027008033	12/1066
85	MF	hormone receptor binding	0.00200875	0.028185498	7/1066
86	MF	voltage-gated sodium channel activity	0.002029916	0.028185498	6/1066
87	MF	transforming growth factor beta binding	0.002029916	0.028185498	6/1066
88	MF	serine hydrolase activity	0.00211748	0.029067222	22/1066
89	MF	transmitter-gated ion channel activity	0.002174393	0.029185181	10/1066
90	MF	transmitter-gated channel activity	0.002174393	0.029185181	10/1066
91	MF	enzyme inhibitor activity	0.002206168	0.029286271	37/1066
92	MF	serine-type endopeptidase activity	0.002650926	0.034807813	20/1066
93	MF	calmodulin binding	0.002904209	0.037723488	22/1066

94	MF	DNA-binding transcription activator activity	0.003019444	0.038803069	42/1066
95	MF	platelet-derived growth factor beta-receptor activity	0.00334983	0.041291788	2/1066
96	MF	collagen V binding	0.00334983	0.041291788	2/1066
97	MF	titin Z domain binding	0.00334983	0.041291788	2/1066
98	MF	voltage-gated ion channel activity involved in regulation of postsynaptic membrane potential	0.00334983	0.041291788	2/1066
99	MF	collagen receptor activity	0.003392145	0.041391027	3/1066
100	MF	cell-cell adhesion mediator activity	0.003566626	0.043084843	9/1066
101	MF	ionotropic glutamate receptor activity	0.003790931	0.044896513	5/1066
102	MF	ephrin receptor activity	0.003790931	0.044896513	5/1066
103	MF	laminin binding	0.003848112	0.045131258	6/1066
104	MF	proteoglycan binding	0.004072355	0.046851475	7/1066
105	MF	neuropeptide receptor binding	0.004072355	0.046851475	7/1066
106	MF	ligand-gated ion channel activity	0.004362921	0.04921903	17/1066
107	MF	ligand-gated channel activity	0.004362921	0.04921903	17/1066
108	MF	DNA-binding transcription activator activity, RNA polymerase II-specific	0.004400377	0.04921903	41/1066

Table S5. The list of KEGG pathway analysis results between the high-risk and low-risk groups

	Description	p-value	p.adjust	GeneRatio	other
1	Neuroactive ligand-receptor interaction	6.07491E-12	1.66453E-09	55/474	Environmental Information Processing
2	ECM-receptor interaction	1.6645E-10	2.28036E-08	24/474	Environmental Information Processing
3	Protein digestion and absorption	5.13846E-09	4.69313E-07	23/474	Organismal Systems
4	Focal adhesion	2.03243E-08	1.39222E-06	34/474	Cellular Processes
5	Calcium signaling pathway	1.11124E-07	6.08957E-06	32/474	Environmental Information Processing
6	cGMP-PKG signaling pathway	5.56202E-07	2.17714E-05	28/474	Environmental Information Processing
7	cAMP signaling pathway	5.0488E-07	2.17714E-05	33/474	Environmental Information Processing
8	Dilated cardiomyopathy (DCM)	7.233E-07	2.4773E-05	20/474	Human Diseases
9	Renin secretion	2.12365E-06	6.46533E-05	16/474	Organismal Systems
10	Hypertrophic cardiomyopathy (HCM)	4.81714E-06	0.00013199	18/474	Human Diseases
11	Vascular smooth muscle contraction	1.01699E-05	0.000232212	22/474	Organismal Systems
12	Arrhythmogenic right ventricular cardiomyopathy (ARVC)	9.7162E-06	0.000232212	16/474	Human Diseases
13	Wnt signaling pathway	2.54424E-05	0.000536247	24/474	Environmental Information Processing
14	AGE-RAGE signaling pathway in diabetic complications	7.97443E-05	0.001456662	17/474	Human Diseases
15	Proteoglycans in cancer	7.7988E-05	0.001456662	27/474	Human Diseases
16	TGF-beta signaling pathway	0.000126765	0.002170844	16/474	Environmental Information Processing
17	PI3K-Akt signaling pathway	0.000143093	0.002306328	39/474	Environmental Information Processing
18	Cell adhesion molecules (CAMs)	0.000165283	0.002515971	21/474	Environmental Information Processing
19	Aldosterone synthesis and secretion	0.000209707	0.0030242	16/474	Organismal Systems
20	Salivary secretion	0.000260554	0.003569587	15/474	Organismal Systems
21	Regulation of lipolysis in adipocytes	0.000342185	0.004464702	11/474	Organismal Systems
22	Adrenergic signaling in cardiomyocytes	0.000541693	0.006453216	20/474	Organismal Systems
23	Malaria	0.000534568	0.006453216	10/474	Human Diseases

24	Circadian entrainment	0.000598177	0.006829189	15/474	Organismal Systems
25	Pertussis	0.001718043	0.018829749	12/474	Human Diseases
26	Retinol metabolism	0.0019187	0.019471257	11/474	Metabolism
27	Renin-angiotensin system	0.001881151	0.019471257	6/474	Organismal Systems
28	Relaxin signaling pathway	0.00425647	0.041652603	16/474	Organismal Systems
29	Cortisol synthesis and secretion	0.004936264	0.045084546	10/474	Organismal Systems
30	Insulin secretion	0.004925198	0.045084546	12/474	Organismal Systems
31	Axon guidance	0.005715855	0.050520787	20/474	Organismal Systems
32	Complement and coagulation cascades	0.007073736	0.060568861	11/474	Organismal Systems
33	Morphine addiction	0.007758535	0.060738247	12/474	Human Diseases
34	Pancreatic secretion	0.007594829	0.060738247	13/474	Organismal Systems
35	Amoebiasis	0.007594829	0.060738247	13/474	Human Diseases
36	Drug metabolism - cytochrome P450	0.010185472	0.077522763	10/474	Metabolism
37	Hippo signaling pathway	0.010481929	0.07762293	17/474	Environmental Information Processing
38	Rap1 signaling pathway	0.014124997	0.09923716	21/474	Environmental Information Processing
39	Human papillomavirus infection	0.014080942	0.09923716	30/474	Human Diseases
40	Metabolism of xenobiotics by cytochrome P450	0.014665529	0.10045887	10/474	Metabolism
41	Pathways in cancer	0.015902736	0.106276823	44/474	Human Diseases
42	Apelin signaling pathway	0.016765231	0.109373177	15/474	Environmental Information Processing
43	MAPK signaling pathway	0.017776693	0.113274744	27/474	Environmental Information Processing
44	IL-17 signaling pathway	0.022598016	0.14072401	11/474	Organismal Systems
45	Chemical carcinogenesis	0.023938397	0.145758241	10/474	Human Diseases
46	Taurine and hypotaurine metabolism	0.024551749	0.146243028	3/474	Metabolism
47	Melanoma	0.027158671	0.158329273	9/474	Human Diseases
48	Hedgehog signaling pathway	0.028243463	0.1612231	7/474	Environmental Information Processing
49	Nicotine addiction	0.030308138	0.162831959	6/474	Human Diseases

50	Regulation of actin cytoskeleton	0.030014737	0.162831959	20/474	Cellular Processes
51	Breast cancer	0.029653478	0.162831959	15/474	Human Diseases
52	Thyroid hormone synthesis	0.031776124	0.167435729	9/474	Organismal Systems
53	Basal cell carcinoma	0.033312868	0.172221243	8/474	Human Diseases
54	PPAR signaling pathway	0.036912551	0.180607837	9/474	Organismal Systems
55	Gap junction	0.036841165	0.180607837	10/474	Cellular Processes
56	Viral protein interaction with cytokine and cytokine receptor	0.036225289	0.180607837	11/474	Environmental Information Processing
57	Phagosome	0.03832718	0.184239426	15/474	Cellular Processes
58	Oxytocin signaling pathway	0.040260474	0.190196034	15/474	Organismal Systems
59	Cytokine-cytokine receptor interaction	0.047400913	0.216464168	25/474	Environmental Information Processing
60	Purine metabolism	0.047099862	0.216464168	13/474	Metabolism
61	Amphetamine addiction	0.049194326	0.220971237	8/474	Human Diseases

Table S6. Transcription factors predicated of the prognostic gene signature

Rank	Transcription Factor	Overlapping Genes	Library	Score
1	CSRNP1	ZFP36, ATF3	ARCHS4 Coexpression, 9; GTEx Coexpression, 16	12.5
2	FOSB	ZFP36, SPARC, GPX3, ATF3	ARCHS4 Coexpression, 7; Enrichr Queries, 41; GTEx Coexpression, 9	19
3	ATOH8	ZFP36, SPARC, GPX3, NOX4, ATF3	ARCHS4 Coexpression, 41; Enrichr Queries, 19; GTEx Coexpression, 13	24.33
4	FOS	ZFP36, SPARC, GPX3, SLC1A5, ATF3	ARCHS4 Coexpression, 24; ENCODE ChIP-seq, 40; Enrichr Queries, 46; ReMap ChIP-seq, 12; GTEx Coexpression, 8	26
5	JUN	ZFP36, SPARC, GPX3, SLC1A5, CP, ATF3	Literature ChIP-seq, 11; ARCHS4 Coexpression, 3; ENCODE ChIP-seq, 1; Enrichr Queries, 129; ReMap ChIP-seq, 16; GTEx Coexpression, 5	27.5
6	TWIST2	SPARC, SLC1A5	ARCHS4 Coexpression, 35; GTEx Coexpression, 23	29
7	ETS2	ZFP36, SPARC, GPX3, ATF3	Literature ChIP-seq, 20; ARCHS4 Coexpression, 23; Enrichr Queries, 26; GTEx Coexpression, 87	39
8	EGR1	ZFP36, SPARC, GPX3, SLC1A5, ATF3	Literature ChIP-seq, 5; ARCHS4 Coexpression, 2; ENCODE ChIP-seq, 68; Enrichr Queries, 27; ReMap ChIP-seq, 135; GTEx Coexpression, 3	40
9	ZBED6	ATF3	ARCHS4 Coexpression, 40	40
10	OSR1	SPARC, NOX4	ARCHS4 Coexpression, 26; GTEx Coexpression, 57	41.5
11	FOXD1	SPARC, SLC1A5	ARCHS4 Coexpression, 60; GTEx Coexpression, 37	48.5
12	MEOX2	SPARC, GPX3, ATF3	ARCHS4 Coexpression, 27; Enrichr Queries, 67; GTEx Coexpression, 54	49.33
13	EPAS1	ZFP36, SPARC, GPX3, CP, ATF3	ARCHS4 Coexpression, 75; Enrichr Queries, 18; ReMap ChIP-seq, 48; GTEx Coexpression, 79	55
14	JUND	ZFP36, SPARC, GPX3, SLC1A5, ATF3	Literature ChIP-seq, 47; ARCHS4 Coexpression, 72; ENCODE ChIP-seq, 13; Enrichr Queries, 121; ReMap ChIP-seq, 78; GTEx Coexpression, 10	56.83
15	MYC	ZFP36, GPX3, NOX4, SLC1A5, ATF3	Literature ChIP-seq, 6; ARCHS4 Coexpression, 22; ENCODE ChIP-seq, 44; Enrichr Queries, 106; ReMap ChIP-seq, 95; GTEx Coexpression, 76	58.17
